# Supplementary material for: Effectiveness and optimal dosage of exercise training for chronic non-specific neck pain: A systematic review with a narrative synthesis
Source: PLoS One. 2020 Jun 10;15(6):e0234511. doi: 10.1371/journal.pone.0234511 (PMC7286530; doi:10.1371/journal.pone.0234511)
Supplement: S2 Appendix — (PDF) [file pone.0234511.s002.pdf]

S2 Appendix Database Searches

Database	Search Strategy
CINAHL (via NICE HDA 1981-)	1 ("neck pain" OR "chronic neck pain" OR "mechanical neck pain" OR cervicalgia OR cervicodynia OR "tension neck syndrome" OR "trapezius myalgia" OR "neck symptoms" OR "neck sprain").af<br>exp "NECK PAIN"/<br>3 exp "NECK INJURIES"/<br>4 (1 OR 2 OR 3)<br>5 ("resistance training" OR "strengthening" OR "muscular endurance" OR "strength endurance" OR "strength-endurance" OR "strength training" OR "craniocervical flexion" OR "cranio-cervical flexion" OR exercise OR "exercise training" OR "exercise program" OR "neck exercise").af<br>6 exp "THERAPEUTIC EXERCISE"/<br>7 exp EXERCISE/<br>8 (5 OR 6 OR 7)<br>9 (Pain OR disability OR "numeric pain rating scale" OR "numeric rating scale" OR "visual analog* scale" OR "visual analog* Pain scale" OR "neck disability index" OR "neck pain and disability scale").af<br>10 exp "PAIN MEASUREMENT"/<br>11 exp "OUTCOMES (HEALTH CARE)"/<br>12 exp "TREATMENT OUTCOMES"/<br>13 exp "ACTIVITIES OF DAILY LIVING"/<br>14 (9 OR 10 OR 11 OR 12 OR 13)<br>15 ("randomi?ed control* trial*" OR "control* clinical trial*" OR "randomi?ed control* clinical trial*" OR RCT).af<br>16 exp "CLINICAL TRIALS"/<br>17 (15 OR 16)<br>18 (4 AND 8 AND 14 AND 17)
MEDLINE (via NICE HDA 1946-)	1 ("neck pain" OR "chronic neck pain" OR "mechanical neck pain" OR cervicalgia OR cervicodynia OR "tension neck syndrome" OR "trapezius myalgia" OR "neck symptoms" OR "neck sprain").af<br>2 exp "NECK PAIN"/<br>3 exp "NECK INJURIES"/<br>4 ("resistance training" OR "strengthening" OR "muscular endurance" OR "strength endurance" OR "strength-endurance" OR "strength training" OR "craniocervical flexion" OR "cranio-cervical flexion" OR exercise OR "exercise training" OR "exercise program" OR "neck exercise").af<br>5 exp "EXERCISE THERAPY"/<br>6 exp EXERCISE/<br>7 (Pain OR disability OR "numeric pain rating scale" OR "numeric rating scale" OR "visual analog* scale" OR "visual analog* Pain scale" OR "neck disability index" OR "neck pain and disability scale").af<br>8 exp "PAIN MEASUREMENT"/<br>9 exp "OUTCOME ASSESSMENT (HEALTH CARE)"/<br>10 exp "TREATMENT OUTCOME"/<br>11 exp "ACTIVITIES OF DAILY LIVING"/<br>12 ("randomi?ed control* trial*" OR "control* clinical trial*" OR "randomi?ed control* clinical trial*" OR RCT).af<br>13 exp "CLINICAL TRIAL"/<br>14 (1 OR 2 OR 3)

	15 (4 OR 5 OR 6)<br>16 (7 OR 8 OR 9 OR 10 OR 11)<br>17 (12 OR 13)<br>18 (14 AND 15 AND 16 AND 17)
EMBASE (via OVID SP 1974-)	1 (neck pain or chronic neck pain or mechanical neck pain or cervicalgia or cervicodynia or tension neck syndrome or trapezius myalgia or neck symptoms or neck sprain).mp. [mp=title, abstract, heading word, drug trade name, original title, device manufacturer, drug manufacturer, device trade name, keyword, floating subheading word, candidate term word] (23038)<br>2 (resistance training or strengthening or muscular endurance or strength endurance or strength-endurance or strength training or craniocervical flexion or cranio-cervical flexion or exercise or exercise training or exercise program or neck exercise).mp. [mp=title, abstract, heading word, drug trade name, original title, device manufacturer, drug manufacturer, device trade name, keyword, floating subheading word, candidate term word] (474366)<br>3 ((Pain or disability or numeric pain rating scale or numeric rating scale or visual analog? scale or visual analog? Pain scale or neck disability index or neck pain) and disability scale).mp. [mp=title, abstract, heading word, drug trade name, original title, device manufacturer, drug manufacturer, device trade name, keyword, floating subheading word, candidate term word] (2712)<br>4 (randomi?ed control* trial* or control* clinical trial* or randomi?ed control* clinical trial* or RCT).mp. [mp=title, abstract, heading word, drug trade name, original title, device manufacturer, drug manufacturer, device trade name, keyword, floating subheading word, candidate term word] (913750)<br>5 exp neck pain<br>6 exp neck injury<br>7 exp kinesiotherapy<br>8 exp exercise<br>9 exp pain measurement<br>10 exp outcome assessment<br>11 exp treatment outcome<br>12 exp "activity of daily living assessment"<br>13 exp "clinical trial (topic)"<br>14 1 or 5 or 6<br>15 2 or 7 or 8<br>16 3 or 9 or 10 or 11 or 12<br>17 4 or 13<br>18 14 and 15 and 16 and 17
PubMed (via NICE HDA 1981-)	1 ("neck pain" OR "chronic neck pain" OR "mechanical neck pain" OR cervicalgia OR cervicodynia OR "tension neck syndrome" OR "trapezius myalgia" OR "neck symptoms" OR "neck sprain").af<br>2 ("resistance training" OR "strengthening" OR "muscular endurance" OR "strength endurance" OR "strength-endurance" OR "strength training" OR "craniocervical flexion" OR "cranio-cervical flexion" OR exercise OR "exercise training" OR "exercise program" OR "neck exercise").af<br>3 (Pain OR disability OR "numeric pain rating scale" OR "numeric rating scale" OR "visual analog* scale" OR "visual analog* Pain scale" OR "neck disability index" OR "neck pain and disability scale").af<br>4 ("randomi?ed control* trial*" OR "control* clinical trial*" OR "randomi?ed control* clinical trial*" OR RCT).af<br>5 (1 AND 2 AND 3 AND 4)

PEDro	Abstract & Title – “neck pain”<br>Therapy – Strength training<br>Problem – Pain<br>Body Part – Head or neck<br>Method – Clinical Trial<br><br>Match all search terms (AND)
OpenGrey	“neck pain”
Zetoc	Conference and Journal Search<br>All Fields “neck pain” “exercise”
Turning Research into Practice	“neck pain” or “chronic neck pain” or “mechanical neck pain” or cervicalgia or cervicodynia or “tension neck syndrome” or “trapezius myalgia” or “neck symptoms” or “neck sprain”<br><br>“resistance training” or “strengthening” or “muscular endurance” or “strength endurance” or “strength-endurance” or “strength training” or “craniocervical flexion” or “cranio-cervical flexion” or exercise or “exercise training” or “exercise program” or “neck exercise”
Index to Chiropractic Literature	“neck pain” AND “exercise”<br>ANY FIELD
Journal of Strength and Conditioning Research	“neck pain”<br>ANY FIELD
Strength and Conditioning Journal	“neck pain”<br>ANY FIELD
European Spine Journal	Neck AND exercise
Spine	“neck pain” and exercise
JOSPT	“neck pain” and exercise
Elsevier (2018 -)	“neck pain” and exercise<br>Title and abstract
Wiley (June 2018 -)	“neck pain” and exercise<br>Abstract

Springer Link (2018 -)	“neck pain” and exercise<br>Title
