# Supplementary material for: Effectiveness and optimal dosage of exercise training for chronic non-specific neck pain: A systematic review with a narrative synthesis
Source: PLoS One. 2020 Jun 10;15(6):e0234511. doi: 10.1371/journal.pone.0234511 (PMC7286530; doi:10.1371/journal.pone.0234511)
Supplement: S3 Appendix — (PDF) [file pone.0234511.s003.pdf]

S3 Appendix Data Extraction Form

Study and General Information

Review title or ID	**Effectiveness and optimal dosage of resistance training for chronic nonspecific neck pain**
Study ID *(surname of first author and publication date of FIRST report of study e.g. Smith 2001)*	
Report ID of other all reports of this study including errata or retractions<br><br>(File number. First Author DATE) (e.g 162. Ahlgren 2001)	
Date form completed *(dd/mm/yyyy)*	
Name/ID of person extracting data	
Study author contact details	
Below box will auto populate from box in bottom table upon saving, closing and reopening file OR CRTL+A/Right Click/update field<br><br>**Notes:**	

Characteristics of included studies

Study Design

	Descriptions as stated in report/paper		Location in text or source *(pg & ¶/fig/table /other)*
**Design** *(e.g. parallel, crossover, non-RCT)*	☐ - Parallel<br>☐ - Crossover<br>☐ - Factorial<br>☐ - Cluster<br>☐ - Non-Randomised<br>☐ - Other – please state		
**Blinding**	☐ - Single<br>☐ - Double<br>☐ - Triple<br>☐ - None<br>☐ - Other – please state		
**Setting** *(e.g office, NHS, private practice, industry, warehouse etc)*			
**Country**			
**Notes:**			

Participants

	Description<br>*Include comparative information for each intervention or comparison group if available*		Location in text or source *(pg & ¶/fig/table/other)*
**Population description** *(gross characteristics of sample population i.e working females, office based workers, sedentary older people, pain levels, disability etc)*			
**Age**	**Mean**		
	**SD**		
	**Range**		
**Gender (n)**	**Females**		
	**Males**		
**Total Randomized**			
**Inclusion criteria**<br>*(use bullet points)*	•		
**Exclusion criteria**<br>*(use bullet points)*	•		
**Baseline Imbalances**	None		
**CONSORT Flow Diagram**	☐ - Yes<br>☐ - No		
**Were Drop outs and loss to follow up adequately reported**	☐ - Yes<br>☐ - No (Provide Details)<br>☐ - Unclear (Provide Details)		
**Drop out/loss to follow up brief details**	<u>Drop outs</u>		
	<u>Loss to follow up</u>		

	**Description**<br>*Include comparative information for each intervention or comparison group if available*					**Location in text or source** *(pg & ¶/fig/table/other)*
Participants in each group	**Total**	**Intervention 1**	**Intervention 2**	**Intervention 3**	**Intervention 4**	
**Allocation**						

			Intervention 1	Intervention 2	Intervention 3	Intervention 4	**Location in text or source** *(pg & ¶/fig/table/other)*
**1**	**Group name**						
**2**	**Theoretical basis/Mechanism by which intervention works reported or discussed in text**		☐ - Yes<br>☐ - No<br>☐ - Unclear	☐ - Yes<br>☐ - No<br>☐ - Unclear	☐ - Yes<br>☐ - No<br>☐ - Unclear	☐ - Yes<br>☐ - No<br>☐ - Unclear	
**3**	**What Materials**<br>NAME: XXXXX<br>MAKE: XXXXXX<br>MODEL: XXXXX<br>*(Include a description of any informational materials used in the intervention, including those provided to participants or used in intervention delivery or in training of intervention providers)*						
**4**	**What Procedures** *(describe each of the procedures, activities, and/or processes used in the intervention, including and enabling or support activities)*	**Structure of Session**					
		**Description of Other Intervention**					
		**Description of Exercise Interventions from text**					
		**HEP**					
		**Other**					
	**Exercise Description** (Check all that apply)		☐ - Upper Limb Resistance Training<br>☐ - Motor Control<br>☐ - Work Capacity – Segmental Conditioning - Functional<br>☐ - Work Capacity – Segmental Conditioning – Non Functional<br>☐ - Work Capacity – Pillar Conditioning - Functional	☐ - Upper Limb Resistance Training<br>☐ - Motor Control<br>☐ - Work Capacity – Segmental Conditioning - Functional<br>☐ - Work Capacity – Segmental Conditioning – Non Functional<br>☐ - Work Capacity – Pillar Conditioning - Functional	☐ - Upper Limb Resistance Training<br>☐ - Motor Control<br>☐ - Work Capacity – Segmental Conditioning - Functional<br>☐ - Work Capacity – Segmental Conditioning – Non Functional<br>☐ - Work Capacity – Pillar Conditioning - Functional	☐ - Upper Limb Resistance Training<br>☐ - Motor Control<br>☐ - Work Capacity – Segmental Conditioning - Functional<br>☐ - Work Capacity – Segmental Conditioning – Non Functional<br>☐ - Work Capacity – Pillar Conditioning - Functional	

			Intervention 1	Intervention 2	Intervention 3	Intervention 4	Location in text or source *(pg & ¶/fig/table/other)*
			☐ - Work Capacity – Pillar Conditioning – Non Functional<br>☐ - Strength – Pillar Strength Development<br>☐ - Strength – Stiffness Development<br>☐ - Strength – Power Development<br>☐ - Other – Please state<br>☐ - NA	☐ - Work Capacity – Pillar Conditioning – Non Functional<br>☐ - Strength – Pillar Strength Development<br>☐ - Strength – Stiffness Development<br>☐ - Strength – Power Development<br>☐ - Other – Please state<br>☐ - NA	☐ - Work Capacity – Pillar Conditioning – Non Functional<br>☐ - Strength – Pillar Strength Development<br>☐ - Strength – Stiffness Development<br>☐ - Strength – Power Development<br>☐ - Other – Please state<br>☐ - NA	☐ - Work Capacity – Pillar Conditioning – Non Functional<br>☐ - Strength – Pillar Strength Development<br>☐ - Strength – Stiffness Development<br>☐ - Strength – Power Development<br>☐ - Other – Please state<br>☐ - NA	
	**Comments/Space for "Other"**						
**5**	***Who Provided*** *(for each category of intervention provider (such as psychologist, nursing assistant) describe their expertise, background, and any specific training given)*	***Professional (N)***					
		Expertise/Years' Experience					
		Interventional Training					
		Other/Comments					
**6**	**How** *(Describe the modes of delivery such as face to face or by some other mechanism, such as internet or telephone) of the intervention and whether it was provided individually or in a group)*	**Face to Face or Other**	☐ - Face to Face<br>☐ - Other – Please state	☐ - Face to Face<br>☐ - Other – Please state	☐ - Face to Face<br>☐ - Other – Please state	☐ - Face to Face<br>☐ - Other – Please state	
		**Individual or Group**	☐ - Individual<br>☐ - Group<br>☐ - Not Clear – See comments	☐ - Individual<br>☐ - Group<br>☐ - Not Clear – See comments	☐ - Individual<br>☐ - Group<br>☐ - Not Clear – See comments	☐ - Individual<br>☐ - Group<br>☐ - Not Clear – See comments	
		**HEP**	☐ - Yes<br>☐ - No<br>☐ - Not Clear – See Comments	☐ - Yes<br>☐ - No<br>☐ - Not Clear – See Comments	☐ - Yes<br>☐ - No<br>☐ - Not Clear – See Comments	☐ - Yes<br>☐ - No<br>☐ - Not Clear – See Comments	
		**Comments**					
**7**	**Where** *(Describe the type(s) of locations(s) where the intervention occurred, including any necessary infrastructure or relevant features*						

			Intervention 1	Intervention 2	Intervention 3	Intervention 4	Location in text or source *(pg & ¶/fig/table/other)*
8	**When and How Much** *(Describe the number of times the intervention was delivered and over what period of time including the number of sessions, their schedule, and their duration, intensity, and dose)*<br><br>**N.B Use Exercise Dosage table for specific sets, reps, load etc for intervention of interest**	***Frequency, duration, number of sessions***					
		Brief description of dosage for each intervention	•	•			
9	**Tailoring** *(If the intervention was planned to be personalised, titrated or adapted, then describe what, why, when and how)*	***Treatment tailored to neuromuscular deficits***	☐ - Yes (Provide Details)<br>☐ - No<br>☐ - Unclear (Provide Details)<br>☐ - NA	☐ - Yes (Provide Details)<br>☐ - No<br>☐ - Unclear (Provide Details)<br>☐ - NA	☐ - Yes (Provide Details)<br>☐ - No<br>☐ - Unclear (Provide Details)<br>☐ - NA	☐ - Yes (Provide Details)<br>☐ - No<br>☐ - Unclear (Provide Details)<br>☐ - NA	
		Individualisation of Treatment Dosage	☐ - Yes (Provide Details)<br>☐ - No<br>☐ - Unclear (Provide Details)<br>☐ - NA	☐ - Yes (Provide Details)<br>☐ - No<br>☐ - Unclear (Provide Details)<br>☐ - NA	☐ - Yes (Provide Details)<br>☐ - No<br>☐ - Unclear (Provide Details)<br>☐ - NA	☐ - Yes (Provide Details)<br>☐ - No<br>☐ - Unclear (Provide Details)<br>☐ - NA	

			Intervention 1	Intervention 2	Intervention 3	Intervention 4	**Location in text or source** *(pg & ¶/fig/table/other)*
**10**	**Modifications** *(If the intervention was modified during the study, describe the changes (what, why, when, and how)*						
**11**	**How Well Planned** *(If intervention adherence or fidelity was assessed, describe how and by whom, and if any strategies were used to maintain or improve fidelity, describe them)*	***Participant adherence to treatment***					
		Fidelity of treatment					
**12**	**How Well Actual** *(If intervention adherence or fidelity was assessed, describe the extent to which the intervention was delivered as planned)*	***Participant adherence to treatment***					
		Fidelity of treatment					
Notes:							

Exercise Dosage for the Intervention of Interest ONLY

(Extract the specific sets, reps, loads and rest for each exercise reported)

**Intervention of interest**							
**Exercise**	**Classification as described by author**	**Our Classification**	**Load**	**Reps**	**Sets**	**Rest**	**Comments**

**Intervention of interest**							
**Exercise**	**Classification as described by author**	**Our Classification**	**Load**	**Reps**	**Sets**	**Rest**	**Comments**

Outcomes

	Pain Outcome Measure		Disability Outcome Measure	Location in text or source *(pg & ¶/fig/table/other)*
**Outcome name**	•		•	
**Time points measured** *(specify whether from start or end of intervention)*	•		•	
**Time points reported**	•		•	
**Was a primary outcome measure identified?**	☐ - Yes (Provide Details)<br>☐ - No<br>☐ - Unclear			
**Was a primary end identified?**	☐ - Yes (Provide Details)<br>☐ - No<br>☐ - Unclear			
**Imputation of missing data** *(e.g. assumptions made for ITT analysis)*	☐ - ITT (Provide Details)<br>☐ - None<br>☐ - Unclear<br>☐ - Other			
**Was a priori power calculation reported**	☐ - Yes<br>☐ - No<br>☐ - Unclear<br>☐ - Other (Please state)			
Notes:				

Data and Analysis – Outcome 1

Copy and paste the appropriate table for each outcome, including additional tables for each time point and subgroup as required.

**Outcome**		
**Description as stated in report/paper**		**Location in text or source** *(pg & ¶/fig/table/other)*
**Summary of Results for Intervention of Interest**		
**Between group differences** *(report important stats including p values)*		
**Plain English Summary**		
**Was Data consistent between reports**	☐ Yes<br>☐ No (Provide comments)<br>☐ Unclear (Provide Comments)<br>☐ NA	
**Reanalysis required?**	☐ Yes<br>☐ No (Provide comments)<br>☐ Unclear (Provide Comments)<br>☐ NA	
**Reanalysis possible?**	☐ Yes<br>☐ No (Provide comments)<br>☐ Unclear (Provide Comments)<br>☐ NA	

COMPLETE PLEASE

Data for Meta-Analysis					
**n.b document if other measure of variance used instead of SD*	**Intervention 1**<br>N (Mean ± Standard Deviation)	**Intervention 2**<br>N (Mean ± Standard Deviation)	**Intervention 3**<br>N (Mean ± Standard Deviation)	**Intervention 4**<br>N (Mean ± Standard Deviation)	
**Group Name**					
**Baseline**					
**Follow Up 1** *(Timeframe)*					
**Follow Up 2** *(Timeframe)*					
**Follow Up 3** *(Timeframe)*					
**Follow Up 4** *(Timeframe)*					
**Any other values reported** *(mean difference, CI etc)*					
**Statistical methods used**					
Notes:					

Outcome		
**Description as stated in report/paper**		**Location in text or source** *(pg & ¶/fig/table/other)*
**Summary of Results for Intervention of Interest**		
**Between group differences** *(report important stats including p values)*		
**Plain English Summary**		

**Was Data consistent between reports**	☐ Yes<br>☐ No (Provide comments)<br>☐ Unclear (Provide Comments)<br>☐ NA		
**Reanalysis required?**	☐ Yes<br>☐ No (Provide comments)<br>☐ Unclear (Provide Comments)<br>☐ NA		
**Reanalysis possible?**	☐ Yes<br>☐ No (Provide comments)<br>☐ Unclear (Provide Comments)<br>☐ NA		

COMPLETE PLEASE

Data for Meta-Analysis					
**n.b document if other measure of variance used instead of SD*	**Intervention 1**<br>N (Mean ± Standard Deviation)	**Intervention 2**<br>N (Mean ± Standard Deviation)	**Intervention 3**<br>N (Mean ± Standard Deviation)	**Intervention 4**<br>N (Mean ± Standard Deviation)	
**Group Name**					
**Baseline**					
**Follow Up 1** *(Timeframe)*					
**Follow Up 2** *(Timeframe)*					
**Follow Up 3** *(Timeframe)*					
**Follow Up 4** *(Timeframe)*					
**Any other values reported** *(mean difference, CI etc)*					
**Statistical methods used**					
Notes:					

Risk of Bias Assessment

(See Handbook Chapter 8. Additional domains may be added for non-randomised studies.)

Domain	***Risk of Bias***				**Support for judgement** *(include direct quotes where available with explanatory comments)*	**Location in text or source** *(pg & ¶/fig/table/other)*
	Low	***High***	***Unclear***	***NA***		
**Random sequence generation** *(selection bias)*	☐	☐	☐	☐		
**Allocation concealment** *(selection bias)*	☐	☐	☐	☐		
**Blinding of participants and personnel** *(performance bias)*	☐	☐	☐	☐		
(if separate judgement by outcome(s) required)	☐	☐	☐	☐	Outcome group:	
**Blinding of outcome assessment** *(detection bias)*	☐	☐	☐	☐	Outcome group:	
(if separate judgement by outcome(s) required)	☐	☐	☐	☐	Outcome group:	
**Incomplete outcome data** *(attrition bias)*	☐	☐	☐	☐		
(if separate judgement by outcome(s) required)	☐	☐	☐	☐	Outcome group:	
**Selective outcome reporting?** (Short Term follow ups) *(reporting bias)*	☐	☐	☐	☐		
**Selective outcome reporting?** (Long Term follow ups) *(reporting bias)*	☐	☐	☐	☐		
**Other bias i.e**<br>• Baseline imbalances<br>• Funding<br>• Fraud<br>• Primary end point<br>• Poor compliance<br>• Primary Outcome Measure<br>• Treatment fidelity assessed or reported<br>• Description of interventions sufficient to replicate?	☐	☐	☐	☐		
**Notes**						

Other information

	Description as stated in report/paper	Location in text or source *(pg & ¶/fig/table/other)*
**References to other relevant studies that need to be screened for eligibility**		
**Correspondence required for further study information** *(from whom, what and when)*		
**Notes:**		
