# Supplementary material for: Effectiveness and optimal dosage of exercise training for chronic non-specific neck pain: A systematic review with a narrative synthesis
Source: PLoS One. 2020 Jun 10;15(6):e0234511. doi: 10.1371/journal.pone.0234511 (PMC7286530; doi:10.1371/journal.pone.0234511)
Supplement: S4 Appendix — (PDF) [file pone.0234511.s004.pdf]

S4 Appendix Risk of Bias

Table 1. Criteria used to determine risk of bias for each domain

Domain	***Risk of Bias***		
	Low	***High***	***Unclear***
**Random sequence generation** (*selection bias*)<br>***Group similarity at baseline (Selection Bias)***	*Random component described in the sequence generation*	*Non- random sequence reported e.g dob, date of admission, preference etc*	*Not reported or reported poorly*
**Allocation concealment** *(selection bias)*	*If participants and person assigning enrolling participants could not foresee assignment*	*If participants or person enrolling participants could work out assignment*	*Not reported or reported poorly*
**Blinding of participants and personnel** *(performance bias)*	*Reported patients were blinded to intervention ONCE allocated and method done so robustly (i.e exercise vs exercise and dosage only difference and documented that participants weren't told dosage was different*	*Participants were aware of different interventions and what they were receiving was different to other groups*<br><br>*Interventions obviously different (i.e exercise vs no treatment OR exercise vs manual therapy)*	*Not reported whether there was any attempt to blind participants from intervention*<br><br>*Exercise vs exercise and blinding not reported*
**Blinding of outcome assessment** *(detection bias)*	*If outcome assessor blinded*	*If outcome assessor aware of treatment allocation*	*If it is not reported or reported poorly*
**Incomplete outcome data** *(attrition bias)*	*No missing outcome data or missing data equal in all groups*<br><br>*ITT analysis used if there are dropouts with description of how it was completed*	*Dropouts not reported adequately 20% dropouts for short term, >30% long term*<br><br>*ITT Not used*	*Unclear whether there was any dropouts*<br><br>*ITT reported but not documented how*
**Selective outcome reporting?** (Short Term follow ups) *(reporting bias)*	*All data presented was recording in protocol*	*Outcome measures used that were not in the protocol*<br><br>*Measures missing that were stated in the protocol*	*If protocol not available*
**Selective outcome reporting?** (Long Term follow ups) *(reporting bias)*	*All data presented was recording in protocol*	*Outcome measures used that were not in the protocol*<br><br>*Measures missing that were stated in the protocol*	*If protocol not available*
**Other bias i.e**<br>• **Baseline Imbalances**<br>• **Funding**<br>• **Fraud**<br>• **Poor compliance**<br>• **Treatment fidelity**	*No baseline imbalances*<br><br>*Attempts taken to monitor how participants were completing HEP throughout the intervention*<br><br>*Adherence reported and high*<br>*No other sources of bias*	*Baselines imbalances*<br><br>*No attempt made to monitor how participants were completing interventions*<br><br>*Adherence reported and low*<br>*Any other sources of bias*	*Adherence not reported*
