# Supplementary material for: Effectiveness and optimal dosage of exercise training for chronic non-specific neck pain: A systematic review with a narrative synthesis
Source: PLoS One. 2020 Jun 10;15(6):e0234511. doi: 10.1371/journal.pone.0234511 (PMC7286530; doi:10.1371/journal.pone.0234511)
Supplement: S5 Appendix — (PDF) [file pone.0234511.s005.pdf]

S5 Appendix GRADE

Criteria by which domains were assessed

Risk of Bias:

Assessed on an outcome level across studies

Imprecision:

Outcomes could be downgraded twice for insufficient sample size and large confidence interval widths.(Guyatt, Oxman et al. 2011) Adequate sample size for pain was calculated using a minimum clinically important difference (MCID) of 10/100 VAS,(Gross, Paquin et al. 2016) alpha = 0.05, and beta = 0.2. The largest standard deviation value was taken from the included studies to generate the largest sample size. The same process was completed for disability using an MCID of 5/50 for NDI.(MacDermid, Walton et al. 2009) Outcomes were marked down for imprecision if the combined sample size <237 disability, (σ=13.68(Ylinen, Hakkinen et al. 2007)) and <248 pain (σ=28(Waling, Jarvholm et al. 2002)). Pooled estimates where the confidence interval overlaps no effect but also included important benefits at the upper or lower limits were downgraded.(Guyatt, Oxman et al. 2011) Where meta-analysis was not possible the confidence intervals of all studies were considered and downgrading was performed if >50% demonstrated no effect and an important benefit at the upper or lower limits.(Murad, Mustafa et al. 2017)

Inconsistency:

Confidence would be downgraded for outcomes where there was *unexplainable* inconsistency in results.(Guyatt, Oxman et al. 2011)

Indirectness:

Where there were concerns over the directness over the population, intervention or outcome measures in answering the research question confidence in the results would be downgraded.(Guyatt, Oxman et al. 2011)

Table 1. Pain: GRADE

RET	Term	RCTS	Population	ROB	Inconsistency	Indirectness	Imprecision	Publication Bias	GRADE	Effectiveness
**Motor Control**										
	Immediate Term	(3 RCTs)	110	serious [a]	not serious [b]	not serious	very serious [c,d]	none	VERY LOW	✗
	Short Term	(8 RCTs)	353	serious [a]	not serious [b]	not serious	not serious	none	MODERATE	✗
	Intermediate Term	(1 RCT)	54	serious [a]	not serious [b]	not serious	serious [c]	none	LOW	✗
**Pillar**										
	Short Term	(5 RCTs)	263	serious [a]	not serious [b]	not serious	not serious	none	MODERATE	✗
	Intermediate Term	(1 RCT)	109	serious [a]	not serious	not serious	serious [c]	none	LOW	✔
**Segmental**										
	Immediate Term	(1 RCT)	48	not serious	not serious	not serious	serious [c]	none	MODERATE	✗
	Short Term	(2 RCTs)	160	serious [a]	not serious [b]	not serious	serious [c]	none	LOW	✗
**Upper Limb**										
	Short Term	(2 RCTs)	190	serious [a]	not serious	not serious	serious [c]	none	LOW	✔
	Intermediate Term	(1 RCT)	87	serious [a]	not serious	not serious	serious [c]	none	LOW	✗
	Long Term	(1 RCT)	126	serious [a]	not serious	not serious	serious [c]	none	LOW	✗
**Motor Control + Pillar**										
	Short Term	(1 RCT)	50	serious [a]	not serious	not serious	serious [c]	none	LOW	✔
**Motor Control + Segmental**										
	Short Term	(2 RCTs)	142	serious [a]	not serious [b]	not serious	serious [c]	none	LOW	✔
**Motor Control + Segmental + Pillar**										

RET	Term	RCTS	Population	ROB	Inconsistency	Indirectness	Imprecision	Publication Bias	GRADE	Effectiveness
	Short Term	(1 RCT)	24	serious [a]	not serious	not serious	serious [c]	none	LOW	✔
**Motor Control + Segmental + Another Intervention**										
	Short Term	(2 RCTs)	278	serious [a]	not serious	not serious	not serious	none	MODERATE	✔
	Intermediate Term	(1 RCT)	218	serious [a]	not serious	not serious	serious [c]	none	LOW	✗
**Pillar + Another Intervention**										
	Short Term	(2 RCTs)	124	very serious [a,e]	not serious	not serious	serious [c]	none	VERY LOW	✗
**Pillar + Upper Limb**										
	Intermediate Term	(1 study)	85	serious [a]	not serious	non serious	serious [c, d]	none	VERY LOW	✗
**Pillar + Upper Limb + Another Intervention**										
	Intermediate Term	(1 RCT)	116	serious [a]	not serious	serious [f]	serious [c]	none	VERY LOW	✗
	Long Term	(1 RCT)	116	serious [a]	not serious	serious [f]	serious [c]	none	VERY LOW	✗
**Segmental + Upper Limb**										
	Short Term	(1 RCT)	52	serious [a]	not serious	not serious	serious [c]	none	LOW	✗
	Intermediate Term	(1 RCT)	43	serious [a]	not serious	not serious	serious [c]	none	LOW	✗
	Long Term	(1 RCT)	41	serious [a]	not serious	not serious	serious [c]	none	LOW	✗
**Segmental + Upper Limb + Another Intervention**										
	Short Term	(1 RCT)	0 [g]	serious [a]	not serious	not serious	serious [c]	none	LOW	✗
	Intermediate Term	(2 RCTs)	0 [g]	serious [a]	not serious	serious [f]	serious [c]	none	VERY LOW	✗
	Long Term	(2 RCTs)	0 [g]	serious [a]	not serious	serious [f]	serious [c]	none	VERY LOW	✗
**Upper Limb + Segmental + Motor Control + Another Intervention**										

RET	Term	RCTS	Population	ROB	Inconsistency	Indirectness	Imprecision	Publication Bias	GRADE	Effectiveness
	Short Term	(1 RCT)	25	serious [a]	not serious	not serious	serious [c]	none	LOW	✗
**XX + Upper Limb**										
	Short Term	(1 RCT)	393	serious [a]	not serious	serious [h]	not serious	none	LOW	✗
	Intermediate Term	(1 RCT)	393	serious [a]	not serious	serious [h]	not serious	none	LOW	✗
	Long Term	(1 RCT)	393	serious [a]	not serious	serious [h]	not serious	none	LOW	✗

[a] patient reported outcome measures used and participants were not blinded
[b] clinical heterogeneity possible confounder for inconsistent results therefore not downgraded
[c] sample size < 248
[d] >50% of data has 95% CI's demonstrating both MCID and no effect
[e] poor randomisation reporting and methods
[f] multi component interventions therefore difficult to allocate effects to RET
[g] sample size not reported
[h] intervention poorly reported to allocatee effects to RET
✗ not effective reducing pain
✔ effective reducing pain

Table 2. Disability: GRADE

RET	Term	RCTS	Population	ROB	Inconsistency	Indirectness	Imprecision	Publication Bias	GRADE	Effectiveness
**Motor Control**										
	Immediate Term	(1 RCT)	28	not serious	not serious [a]	not serious	serious [b]	none	MODERATE	✗
	Short Term	(8 RCTs)	353	serious [c]	not serious [a]	not serious	not serious	none	MODERATE	✗

RET	Term	RCTS	Population	ROB	Inconsistency	Indirectness	Imprecision	Publication Bias	GRADE	Effectiveness
	Intermediate Term	(1 RCT)	54	serious [c]	not serious [a]	not serious	serious [b]	none	LOW	✗
**Pillar**										
	Short Term	(5 RCTs)	263	serious [c]	not serious [a]	not serious	not serious	none	MODERATE	✗
	Intermediate Term	(1 RCT)	109	serious [c]	not serious	not serious	serious [b]	none	LOW	✔
**Segmental**										
	Short Term	(2 RCTs)	160	serious [c]	not serious [a]	not serious	serious [b]	none	LOW	✗
**Motor Control + Pillar**										
	Short Term	(1 RCTs)	50	serious [c]	not serious [a]	not serious	serious [b]	none	LOW	✔
**Motor Control + Segmental**										
	Short Term	(2 RCTs)	142	serious [c]	not serious [a]	not serious	serious [b]	none	LOW	✗
**Motor Control + Segmental + Pillar**										
	Short Term	(1 RCT)	24	serious [c]	not serious	not serious	serious [b]	none	LOW	✔
**Motor Control + Segmental + Another Intervention**										
	Short Term	(2 RCTs)	278	serious [c]	not serious	not serious	not serious	none	MODERATE	✔
	Intermediate Term	(1 RCT)	218	serious [c]	not serious	not serious	serious [b]	none	LOW	✗
**Pillar + Another Intervention**										
	Short Term	(2 RCTs)	124	very serious [c,d]	not serious	not serious	serious [b]	none	VERY LOW	✗
**Pillar + Upper Limb + Another Intervention**										

RET	Term	RCTS	Population	ROB	Inconsistency	Indirectness	Imprecision	Publication Bias	GRADE	Effectiveness
	Short Term	(1 RCT)	116	serious [c]	not serious	serious [e]	serious [b]	none	VERY LOW	✗
	Intermediate Term	(1 RCT)	116	serious [c]	not serious	serious [e]	serious [b]	none	VERY LOW	✗
	Long Term	(1 RCT)	116	serious [c]	not serious	serious [e]	serious [b]	none	VERY LOW	✗
**Segmental + Upper Limb**										
	Short Term	(1 RCT)	52	serious [c]	not serious	not serious	serious	none	LOW	✗
	Intermediate Term	(1 RCT)	43	serious [c]	not serious	not serious	serious	none	LOW	✗
	Long Term	(1 RCT)	41	serious [c]	not serious	not serious	serious	none	LOW	✗
**Segmental + Upper Limb + Another Intervention**										
	Short Term	(2 RCTs)	0 [f]	serious [c]	not serious	serious [e]	serious [b]	none	VERY LOW	✗
	Intermediate Term	(2 RCTs)	0 [f]	serious [c]	not serious	serious [e]	serious [b]	none	VERY LOW	✗
	Long Term	(2 RCTs)	0 [f]	serious [c]	not serious	serious [e]	serious [b]	none	VERY LOW	✗
**Upper Limb + Segmental + Motor Control + Another Intervention**										
	Short Term	(1 RCT)	25	serious [c]	not serious	not serious	serious [b]	none	LOW	✗
**XX + Upper Limb**										
	Short Term	(1 RCT)	393	serious [c]	not serious	serious [g]	not serious	none	LOW	✗
	Intermediate Term	(1 RCT)	393	serious [c]	not serious	serious [g]	not serious	none	LOW	✗
	Long Term	(1 RCT)	393	serious [c]	not serious	serious [g]	not serious	none	LOW	✗

RET	Term	RCTS	Population	ROB	Inconsistency	Indirectness	Imprecision	Publication Bias	GRADE	Effectiveness

[a] clinical heterogeneity possible confounder for inconsistent results therefore not downgraded
[b] sample size < 237
[c] patient reported outcome measures used and participants were not blinded
[d] poor randomisation reporting and methods
[e] multi component interventions therefore difficult to allocate effects to RET
[f] sample size not reported
[g] intervention poorly reported to allocation effects to RET
✗not effective reducing disability
✔ effective reducing disability

References:

Gross, A. R., J. P. Paquin, G. Dupont, S. Blanchette, P. Lalonde, T. Cristie, N. Graham, T. M. Kay, S. J. Burnie, G. Gelley, C. H. Goldsmith, M. Forget, P. L. Santaguida, A. J. Yee, G. G. Radisic, J. L. Hoving, G. Bronfort and G. Cervical Overview (2016). "Exercises for mechanical neck disorders: A Cochrane review update." Man Ther **24**: 25-45.

Guyatt, G. H., A. D. Oxman, R. Kunz, J. Brozek, P. Alonso-Coello, D. Rind, P. J. Devereaux, V. M. Montori, B. Freyschuss, G. Vist, R. Jaeschke, J. W. Williams, Jr., M. H. Murad, D. Sinclair, Y. Falck-Ytter, J. Meerpohl, C. Whittington, K. Thorlund, J. Andrews and H. J. Schunemann (2011). "GRADE guidelines 6. Rating the quality of evidence--imprecision." J Clin Epidemiol **64**(12): 1283-1293.

Guyatt, G. H., A. D. Oxman, R. Kunz, J. Woodcock, J. Brozek, M. Helfand, P. Alonso-Coello, Y. Falck-Ytter, R. Jaeschke, G. Vist, E. A. Akl, P. N. Post, S. Norris, J. Meerpohl, V. K. Shukla, M. Nasser, H. J. Schunemann and G. W. Group (2011). "GRADE guidelines: 8. Rating the quality of evidence--indirectness." J Clin Epidemiol **64**(12): 1303-1310.

Guyatt, G. H., A. D. Oxman, R. Kunz, J. Woodcock, J. Brozek, M. Helfand, P. Alonso-Coello, P. Glasziou, R. Jaeschke, E. A. Akl, S. Norris, G. Vist, P. Dahm, V. K. Shukla, J. Higgins, Y. Falck-Ytter, H. J. Schunemann and G. W. Group (2011). "GRADE guidelines: 7. Rating the quality of evidence--inconsistency." J Clin Epidemiol **64**(12): 1294-1302.

MacDermid, J. C., D. M. Walton, S. Avery, A. Blanchard, E. Etruw, C. McAlpine and C. H. Goldsmith (2009). "Measurement properties of the neck disability index: a systematic review." J Orthop Sports Phys Ther **39**(5): 400-417.

Murad, M. H., R. A. Mustafa, H. J. Schunemann, S. Sultan and N. Santesso (2017). "Rating the certainty in evidence in the absence of a single estimate of effect." Evid Based Med **22**(3): 85-87.

Waling, K., B. Jarvholm and G. Sundelin (2002). "Effects of training on female trapezius Myalgia: An intervention study with a 3-year follow-up period." Spine (Phila Pa 1976) **27**(8): 789-796.

Ylinen, J., A. Hakkinen, M. Nykanen, H. Kautiainen and E. P. Takala (2007). "Neck muscle training in the treatment of chronic neck pain: a three-year follow-up study." Eura Medicophys **43**(2): 161-169.
