# Supplementary material for: Effectiveness and optimal dosage of exercise training for chronic non-specific neck pain: A systematic review with a narrative synthesis
Source: PLoS One. 2020 Jun 10;15(6):e0234511. doi: 10.1371/journal.pone.0234511 (PMC7286530; doi:10.1371/journal.pone.0234511)
Supplement: S6 Appendix — (PDF) [file pone.0234511.s006.pdf]

S6 Appendix Excluded citations with reasons for ineligibility

Duplicates	(Alpayci 2017, Andersen 2012, Andersen 2013, Ang 2009, Arja 2008, Beltran-Alacreu 2015, Brage 2015, Bronfort 2001, Bronfort 2001, Bronfort 2001, Chiu 2005, Chiu 2005, Evans 2002, Evans 2012, Falla D, Lindstrom R et al. 2014, Falla 2013, Galindez-Ibarbengoetxea, Setuain et al. 2018, Gallego Izquierdo 2016, Jordan 1998, JOSPT 2003, Karlsson 2014, Lansinger 2007, Lansinger 2007, Leininger 2016, Lin 2018, Maiers 2014, Martel 2011, Monticone 2017, O'Leary 2007, O'Leary 2012, Rasotto 2015, Rendant 2011, Rolving 2014, Salo 2012, Scheel 2003, Sionnadh 2013, Thompson 2016, Vihstadt 2014, Viljanen 2003, von Trott 2009, Waling 2002, Waling 2002, Yildiz, Turgut et al. 2018, Ylinen 2006, Zaproudina 2007)
Non-English	(Arami 2012, Kawarada 2016, Mohammadi 2014, Taheri 2012)
Not RCT	(Beneka 2014, Bregeon 2003, Chung 2012, George 2008, Lawrence 2005, Lluch 2013, Nadler 2003, Nazari, Bobos et al. 2018, O Leary 2003, Oda 2003, Park 2006, Personnal Today 2004, Reinikka 2009, Scheel 2003, Stenchever 2003, Yelland 2003)
Protocols (all protocols were hand search for full study texts)	(Bronfort 2005, Erlandsson and Carlsson 2018, Fimland 2015, **Hsieh 2012***, Iqbal 2018, Kashfi, Karimi et al. 2019, Silva 2018, Sjøgaard 2013, Witt 2008, Yildiz 2018, Ylinen 2010)
Study not completed	(Giménez-Costa 2018)
Unable to access	(Alternative and Complementary Therapies 2003, Cho, Lee et al. 2019, Fariba 2016, Ferrari 2006, Ghaderi 2017, Gore, Sepic et al. 1987, Ha, Kwon et al. 2011, Highland, Dreisinger et al. 1992, Kaminski 2011, Petrofsky 2017, Riveiro 2003, Rothstein 2001, Van Dillen, McDonnell et al. 2007, Westaway 2003, Wilson 2001, Ylinen 2004, Καρμίρης 2012)
Wrong Intervention	(Beltran-Alacreu 2015, Bertozzi 2015, Boyoung 2016, Brage 2015, Cen 2003, Cramer 2013, de Araujo Cazotti 2018, Dunleavy 2016, Farooq 2018, Fathollahnejad, Letafatkar et al. 2019, Ghafouri 2014, Gialanella 2017, Giombini 2013, Hagberg 2000, Hakkinen 2008, Hildreth 2015, Iversen, Vasseljen et al. 2018, Journal of Alternative & Complementary Medicine 2016, Karlsson 2015, Karlsson 2014, Lansinger 2013, Lansinger 2007, Lauche 2016, Lauche 2017, Lluch 2014, Lluch 2014, Lundqvist 2014, Marco 2016, Martel 2011, Park 2016, Rajalaxmi 2018, Ravi 2016, Rendant 2011, Salo 2012, Sarig Bahat 2015, Senthil 2016, Shih 2008, Taimela 2000, Telci 2012, Thompson 2018, Treleaven 2018, Vonk 2009, Woby 2015, Ylinen 2006, Zaproudina 2007)
Wrong Outcome Measures	(O'Leary 2007, Salo 2010, Ylinen 2005)
Wrong Population	(Abdel-aziem 2016, Ali 2014, Alpayci 2017, **Andersen 2014†, Andersen 2012†, Andersen 2013†, Andersen 2008†, Andersen 2009†, Andersen 2012†, Andersen 2014†, Andersen 2010†, Andersen 2008†, Andersen 2008†, Andersen 2011†**, Ang 2009, Berg, Berggren et al. 1994, Bid 2014, **Blangsted 2008†**, Bronfort 2005, Bronfort 1998, Dalager 2015, Dalager 2017, Dellve 2011, Dusunceli 2009, El-Abd 2017, Evans 2002, Evans 2012, Falla 2006, Falla 2007, Falla 2008, Galindez-Ibarbengoetxea 2017, Galindez-Ibarbengoetxea 2018, Ganesh 2015, Gram 2014, Griffiths 2009, Groeneweg 2017, Helewa 2007, Hoving 2006, Hoving 2002, Iqbal, Rajan et al. 2013, Irfan, Sharif et al. 2018, Jakobsen 2015, Jamal 2016, Jay 2013, Jensen 2015, **Jensen 2014†**, Jull 2009, Jull 2007, Kaka 2018, Kietrys 2007, Kim 2018, Kjellman 2002, Klemetti 1997, Lange 2013, Leininger 2016, Levoska and Keinanen-Kiukaanniemi 1993, Lin 2018, Ma 2011, Maiers 2014, Manca 2007, McLean 2007, McLean 2013, Mortensen 2014, Murray 2017, Myhre 2014, Nezamuddin 2013, Nezamuddin 2013, Nielsen 2010, O'Leary 2012, Oldervoll 2001, OsteloM.; 2016, Paoloni 2013, Pedersen 2013, **Pedersen 2013†**, Pereira, Comans et al. 2019, Pillastrini 2009, Raja 2015, Raju, Apparao et al. 2019, Rasotto 2015, Rasotto 2015, Ratzon 2016, **Savolainen 2004†**, Shariat 2017, Sharmila 2014, Shenoy 2010, Sjögren 2005, Skelly 2016, Søgaard 2012, Spine 2009, Suni 2017, Tsauo 2004, Vasseljen 1995, Vihstadt 2014, von Trott 2009, Wang, Olson et al. 2003, Wani 2013, **Zebis 2011†, Zebis 2014†**)

*clarification sought for full study data, but authors did not respond; **†**authors contacted to clarify whether 100% of participants had symptoms >3 months, but authors did not respond

Full References:

Abdel-aziem, A. A. D., Amira Hussin (2016). "Efficacy of deep neck flexor exercise for neck pain: a randomized controlled study." Turkish Journal of Physical Medicine & Rehabilitation / Turkiye Fiziksel Tip ve Rehabilitasyon Dergisi **62**(2): 107-115.

Ali, A. S.-U.-R., Syed; Sibtain, Fozia (2014). "The efficacy of Sustained Natural Apophyseal Glides with and without Isometric Exercise Training in Non-specific Neck Pain." Pakistan journal of medical sciences **30**(4): 872-874.

Alpayci, M. İ., Server (2017). "Isometric Exercise for the Cervical Extensors Can Help Restore Physiological Lordosis and Reduce Neck Pain." American journal of physical medicine & rehabilitation **96, NUMB 9**: 621-626.

Alpayci, M. İ., Server (2017). "Isometric Exercise for the Cervical Extensors Can Help Restore Physiological Lordosis and Reduce Neck Pain: A Randomized Controlled Trial." American Journal of Physical Medicine & Rehabilitation **36**(5): 621-626.

Alternative and Complementary Therapies (2003). "News You Can Use: More exercise recommended to relieve chronic pain in the neck." Alternative & Complementary Therapies **9**(4): 156-156.

Andersen, C. A., Lars; Zebis, Mette; Sjøgaard, Gisela (2014†). "Effect of Scapular Function Training on Chronic Pain in the Neck/Shoulder Region: A Randomized Controlled Trial." Journal of Occupational Rehabilitation **24**(2): 316-324.

Andersen, C. H. A., L. L.; Gram, B.; Pedersen, M. T.; Mortensen, O. S.; Zebis, M. K.; Sjogaard, G. (2012). "Influence of frequency and duration of strength training for effective management of neck and shoulder pain: a randomised controlled trial [with consumer summary]." British Journal of Sports Medicine 2012 Nov;46(14):1004-1010.

Andersen, C. H. A., L. L.; Gram, B.; Pedersen, M. T.; Mortensen, O. S.; Zebis, M. K.; Sjøgaard, G.; Andersen, Christoffer H.; Andersen, Lars L.; Gram, Bibi; Pedersen, Mogens Theisen; Mortensen, Ole Steen; Zebis, Mette Kreutzfeldt; Sjøgaard, Gisela (2012†). "Influence of frequency and duration of strength training for effective management of neck and shoulder pain: a randomised controlled trial." British Journal of Sports Medicine **46**(14): 1004-1010.

Andersen, C. H. A., L. L.; Pedersen, M. T.; Mortensen, P.; Karstad, K.; Mortensen, O. S.; Zebis, M. K.; Sjogaard, G. (2013). "Dose-response of strengthening exercise for treatment of severe neck pain in women [with consumer summary]." Journal of Strength & Conditioning Research 2013 Dec;27(12):3322-3328.

Andersen, C. H. A., Lars L.; Pedersen, Mogens T.; Mortensen, Peter; Karstad, Kristina; Mortensen, Ole S.; Zebis, Mette K.; SjÃ¸gaard, Gisela (2013†). "DOSE-RESPONSE OF STRENGTHENING EXERCISE FOR TREATMENT OF SEVERE NECK PAIN IN WOMEN." Journal of Strength & Conditioning Research (Lippincott Williams & Wilkins) **27**(12): 3322-3328.

Andersen, L. L. A., C. H.; Sundstrup, E.; Jakobsen, M. D.; Mortensen, O. S.; Zebis, M. K. (2012†). "Central adaptation of pain perception in response to rehabilitation of musculoskeletal pain: Randomized controlled trial." Pain Physician **15**(5): 385-393.

Andersen, L. L. A., Christoffer H.; Skotte, Jørgen H.; Suetta, Charlotte; Søgaard, Karen; Saltin, Bengt; Sjøgaard, Gisela (2014†). "High-Intensity Strength Training Improves Function of Chronically Painful Muscles: Case-Control and RCT Studies." BioMed Research International **2014**: 187324-187324.

Andersen, L. L. A., Christoffer H.; Zebis, Mette K.; Nielsen, Pernille K.; Søgaard, Karen; Sjøgaard, Gisela (2008†). "Effect of physical training on function of chronically painful muscles: a randomized controlled trial." Journal of applied physiology (Bethesda, Md. : 1985) **105**(6): 1796-1801.

Andersen, L. L. A., Jesper L.; Suetta, Charlotte; Kjaer, Michael; Søgaard, Karen; Sjøgaard, Gisela (2009†). "Effect of contrasting physical exercise interventions on rapid force capacity of chronically painful muscles." Journal of applied physiology (Bethesda, Md. : 1985) **107**(5): 1413-1419.

Andersen, L. L. C., K. B.; Holtermann, A.; Poulsen, O. M.; Sjøgaard, G.; Pedersen, M. T.; Hansen, E. A. (2010†). "Effect of physical exercise interventions on musculoskeletal pain in all body regions among office workers: a one-year randomized controlled trial." Manual Therapy **15**(1): 100-104.

Andersen, L. L. J., M. B.; Blangsted, A. K.; Pedersen, M. T.; Hansen, E. A.; Sjøgaard, G. (2008†). "A randomized controlled intervention trial to relieve and prevent neck/shoulder pain." Medicine & Science in Sports & Exercise **40**(6): 983-990.

Andersen, L. L. K., M.; Sogaard, K.; Hansen, L.; Kryger, A. I.; Sjogaard, G. (2008†). "Effect of two contrasting types of physical exercise on chronic neck muscle pain." Arthritis Care and Research **59**(1): 84-91.

Andersen, L. L. S., C. A.; Mortensen, O. S.; Poulsen, O. M.; Hannerz, H.; Zebis, M. K. (2011†). "Effectiveness of small daily amounts of progressive resistance training for frequent neck/shoulder pain: Randomised controlled trial." Pain (03043959) **152**(2): 440-446.

Ang, B. O. M., A.; Harms-Ringdahl, K. (2009). "Neck/shoulder exercise for neck pain in air force helicopter pilots: a randomized controlled trial." Spine (03622436) **34**(16).

Ang, B. O. M., A.; Harms-Ringdahl, K. (2009). "Neck/shoulder exercise for neck pain in air force helicopter pilots: a randomized controlled trial [with consumer summary]." Spine 2009 Jul 15;34(16):E544-E551.

Arami, J. R., A.; Khalkhali Zaavieh, M.; Rahnama, L. (2012). "The effect of two exercise therapy programs (proprioceptive and endurance training) to treat patients with chronic non-specific neck pain." Journal of Babol University of Medical Sciences **14**(1): 78-84.

Arja, H. k. H., Kautiainen; Pekka, Hannonen; Jari, Ylinen (2008). "Strength training and stretching versus stretching only in the treatment of patients with chronic neck pain: a randomized one-year follow-up study." EvidenceUpdates **22**(7): 592.

Beltran-Alacreu, H. L.-d.-U.-V., I.; Fernandez-Carnero, J.; La Touche, R. (2015). "Manual Therapy, Therapeutic Patient Education, and Therapeutic Exercise, an Effective Multimodal Treatment of Nonspecific Chronic Neck Pain: A Randomized Controlled Trial." American journal of physical medicine & rehabilitation / Association of Academic Physiatrists **94**(10 Supplement 1): 887-897.
Beltran-Alacreu, H. L.-d.-U.-V., Ibai; Fernández-Carnero, Josué; La Touche, Roy (2015). "Manual Therapy, Therapeutic Patient Education, and Therapeutic Exercise, an Effective Multimodal Treatment of Nonspecific Chronic Neck Pain." American journal of physical medicine & rehabilitation **94**: 887-897.
Beneka, A. M., P.; Gioftsidou, A. (2014). "Neck Pain and Office Workers : An Exercise Program for the Workplace." Acsms Health and Fitness Journal **18, NUMB 3**: 18-24.
Berg, H. E., G. Berggren and P. A. Tesch (1994). "Dynamic neck strength training effect on pain and function." Archives of Physical Medicine & Rehabilitation **75**.
Bertozzi, L. V., Jorge H.; Capra, Francesco; Reci, Marsida; Pillastrini, Paolo (2015). "Effect of an Exercise Programme for the Prevention of Back and Neck Pain in Poultry Slaughterhouse Workers." Occupational Therapy International **22**(1): 36-42.
Bid, D. R., A. Thangamani; Bhatt, Jahnvi A.; Rathod, Prerna N.; Tandel, Krupali V.; Tandel, Soniya S. (2014). "The effectiveness of Mechanical Cervical Traction on Patients with Unilateral Mechanical Neck Pain." Indian Journal of Physiotherapy & Occupational Therapy **8**(3): 97-103.
Blangsted, A. K. S., Karen; Hansen, Ernst A.; Hannerz, Harald; Sjøgaard, Gisela (2008†). "One-year randomized controlled trial with different physical-activity programs to reduce musculoskeletal symptoms in the neck and shoulders among office workers." Scandinavian journal of work, environment & health **34**(1): 55-65.
Boyoung, I. M. Y., K. I. M.; Yijung, Chung; Sujin, Hwang (2016). "Effects of scapular stabilization exercise on neck posture and muscle activation in individuals with neck pain and forward head posture." Journal of Physical Therapy Science **28**(3): 951-955.
Brage, K. R., I.; Falla, D.; Søgaard, K.; Juul-Kristensen, B. (2015). "Pain education combined with neck- and aerobic training is more effective at relieving chronic neck pain than pain education alone--A preliminary randomized controlled trial." Manual therapy **20**(5): 686-693.
Brage, K. R., I.; Falla, D.; Søgaard, K.; Juul-Kristensen, B. (2015). "Pain education combined with neck- and aerobic training is more effective at relieving chronic neck pain than pain education alone – A preliminary randomized controlled trial." Manual Therapy **20**(5): 686-693.
Bregeon, F. (2003). "Dynamic neck muscle training or relaxation does not improve chronic neck pain." Australian Journal of Physiotherapy **49**(4): 276-276.
Bronfort, G. (2005). "Chiropractic and Exercise for Seniors With Neck Pain." Clinical Trials.
Bronfort, G. (2005). "Manipulation, Exercise, and Self-Care for Neck Pain." Clinical Trials.
Bronfort, G. E., R.; Nelson, B.; Aker, P. D.; Goldsmith, C. H.; Vernon, H. (1998). A randomized clinical trial of spinal manipulation and exercise for chronic neck pain : A report on neck performance outcomes after 11 weeks and long-term effects on patient-rated outcomes. Spinal manipulation, Vancouver, Canada, Foundation for Chiropractic Education and Research, 1998.
Bronfort, G. E., R.; Nelson, B.; Aker, P. D.; Goldsmith, C. H.; Vernon, H. (2001). "A Randomized Clinical Trial of Exercise and Spinal Manipulation for Patients With Chronic Neck Pain." Spine **26, PART 7**: 788-797.
Bronfort, G. E., R.; Nelson, B.; Aker, P. D.; Goldsmith, C. H.; Vernon, H. (2001). "A randomized clinical trial of exercise and spinal manipulation for patients with chronic neck pain [with consumer summary]." Spine 2001 Apr 1;26(7):788-797.
Bronfort, G. E., R.; Nelson, B.; Aker, P. D.; Goldsmith, C. H.; Vernon, H. (2001). "A randomized clinical trial of exercise and spinal manipulation for patients with chronic neck pain...including commentary by Swenson RS." Spine (03622436) **26**(7): 788-799.
Cen, S. Y. L., S. F.; Sletten, E. G.; McLaine, A. (2003). "The effect of traditional Chinese therapeutic massage on individuals with neck pain." Clinical Acupuncture & Oriental Medicine **4**(2-3): 88-93.
Chiu, T. T. W. H.-C., C. W. Y.; Cheing, G. (2005). "A randomized clinical trial of TENS and exercise for patients with chronic neck pain." Clinical Rehabilitation **19**(8): 850-860.
Chiu, T. T. W. L., T.; Hedley, A. J. (2005). "A randomized controlled trial on the efficacy of exercise for patients with chronic neck pain." Spine (03622436) **30**(1).
Cho, J., E. Lee and S. Lee (2019). "Upper cervical and upper thoracic spine mobilization versus deep cervical flexors exercise in individuals with forward head posture: A randomized clinical trial investigating their effectiveness." Journal of Back & Musculoskeletal Rehabilitation **32**(4): 595-602.
Chung, S. H. (2012). "Effects of Exercise on Deep Cervical Flexors in Patients with Chronic Neck Pain." Journal of Physical Therapy Science **24, NUMB 7**: 629-632.
Cramer, H. L., R.; Hohmann, C.; Lüdtke, R.; Haller, H.; Michalsen, A.; Langhorst, J.; Dobos, G. (2013). "Randomized-controlled Trial Comparing Yoga and Home-based Exercise for Chronic Neck Pain." Clinical Journal of Pain **29**(3): 216-223.
Dalager, T. B., T. G. V.; Pedersen, M. T.; Boyle, E.; Andersen, L. L.; Sjogaard, G. (2015). "Does training frequency and supervision affect compliance, performance and muscular health? A cluster randomized controlled trial." Manual Therapy **20**(5): 657-665.
Dalager, T. J., Just Bendix; Sjøgaard, Gisela (2017). "Intelligent Physical Exercise Training in a Workplace Setting Improves Muscle Strength and Musculoskeletal Pain: A Randomized Controlled Trial." BioMed Research International: 1-9.
de Araujo Cazotti, L. J., A.; Roger-Silva, D.; Ribeiro, L. H. C.; Natour, J. (2018). "Effectiveness of the Pilates Method in the Treatment of Chronic Mechanical Neck Pain: A Randomized Controlled Trial." Archives of Physical Medicine and Rehabilitation.
Dellve, L. A., L.; Jonsson, A.; Sandsjo, L.; Forsman, M.; Lindegard, A.; Ahlstrand, C.; Kadefors, R.; Hagberg, M. (2011). "Myofeedback training and intensive muscular strength training to decrease pain and improve work ability among female workers on long-term sick leave with neck pain: A randomized controlled trial." International Archives of Occupational and Environmental Health **84**(3): 335-346.
Dunleavy, K. K., K.; Goldberg, A.; Malek, M. H.; Talley, S. A.; Tutag-Lehr, V.; Hildreth, J. (2016). "Comparative effectiveness of Pilates and yoga group exercise interventions for chronic mechanical neck pain: quasi-randomised parallel controlled study." Physiotherapy (United Kingdom) **102**(3): 236-242.
Dusunceli, Y. O., C.; Atamaz, F.; Hepguler, S.; Durmaz, B. (2009). "Efficacy of neck stabilization exercises for neck pain: a randomized controlled study." Journal of Rehabilitation Medicine (Stiftelsen Rehabiliteringsinformation) **41**(8): 626-631.
El-Abd, A. M. I., Abeer R.; El-Hafez, Haytham M. (2017). "Efficacy of kinesio taping versus postural correction exercises on pain intensity and axioscapular muscles activation in mechanical neck dysfunction: a randomized blinded clinical trial." Journal of Sports Medicine & Physical Fitness **57**(10): 1311-1317.
Erlandsson, L.-K. and J. Y. Carlsson (2018). "Effects of Qigong and Exercise Therapy Among Patients With Chronic Neck Pain." Clinical Trials.
Evans, R. B., G.; Nelson, B.; Goldsmith, C. H. (2002). "Two-year follow-up of a randomized clinical trial of spinal manipulation and two types of exercise for patients with chronic neck pain." Spine (03622436) **27**(21): 2383-2389.
Evans, R. B., G.; Nelson, B.; Goldsmith, C. H. (2002). "Two-year follow-up of a randomized clinical trial of spinal manipulation and two types of exercise for patients with chronic neck pain [with consumer summary]." Spine 2002 Nov 1;27(21):2383-2389.
Evans, R. B., G.; Schulz, C.; Maiers, M.; Bracha, Y.; Svendsen, K.; Grimm, R.; Garvey, T.; Transfeldt, E. (2012). "Supervised exercise with and without spinal manipulation performs similarly and better than home exercise for chronic neck pain: a randomized controlled trial." Spine (03622436) **37**(11): 903-914.
Evans, R. B., G.; Schulz, C.; Maiers, M.; Bracha, Y.; Svendsen, K.; Grimm, R.; Garvey, T.; Transfeldt, E. (2012). "Supervised exercise with and without spinal manipulation performs similarly and better than home exercise for chronic neck pain: a randomized controlled trial [with consumer summary]." Spine 2012 May 15;37(11):903-914.
Falla D, Lindstrom R, Rechter L, Boudreau S and P. F (2014). "Effectiveness of an 8-week exercise programme on pain and specificity of neck muscle activity in patients with chronic neck pain : A randomized controlled study." **17**(10).
Falla, D. J., G.; Hodges, P. (2008). "Training the cervical muscles with prescribed motor tasks does not change muscle activation during a functional activity." Manual Therapy **13**(6): 507-512.
Falla, D. J., G.; Hodges, P.; Vicenzino, B. (2006). "An endurance-strength training regime is effective in reducing myoelectric manifestations of cervical flexor muscle fatigue in females with chronic neck pain." Clinical Neurophysiology **117**(4): 828-837.
Falla, D. J., G.; Russell, T.; Vicenzino, B.; Hodges, P. (2007). "Effect of neck exercise on sitting posture in patients with chronic neck pain." Physical Therapy **87**(4): 408-417.
Falla, D. L., R.; Rechter, L.; Boudreau, S.; Petzke, F. (2013). "Effectiveness of an 8?week exercise programme on pain and specificity of neck muscle activity in patients with chronic neck pain : A randomized controlled study." European Journal of Pain **17, ISSU 10**: 1517-1528.
Fariba, G. M. A., Jafarabadi; Khodabakhsh, Javanshir (2016). "The clinical and EMG assessment of the effects of stabilization exercise on nonspecific chronic neck pain: A randomized controlled trial." Journal of back and musculoskeletal rehabilitation **30**(2): 211.
Farooq, M. N. M.-B., M. A.; Gilani, S. A.; Ashfaq, M.; Mahmood, Q. (2018). "The effects of neck mobilization in patients with chronic neck pain: A randomized controlled trial." Journal of Bodywork and Movement Therapies **22**(1): 24-31.
Fathollahnejad, K., A. Letafatkar and M. Hadadnezhad (2019). "The effect of manual therapy and stabilizing exercises on forward head and rounded shoulder postures: a six-week intervention with a one-month follow-up study." BMC Musculoskeletal Disorders **20**(1).
Ferrari, R. F., E.; Birbaumer, N. (2006). "Individual characteristics and results of biofeedback training and operant treatment in patients with chronic pain." Psicoterapia Cognitiva e Comportamentale **12**(2): 161-179.
Fimland, M. S. (2015). "Effects of Resistance Training on Pain, Function and Work Ability in Patients With Moderate to Severe Neck Pain." Clinical Trials.
Galindez-Ibarbengoetxea, X., I. Setuain, R. Ramírez-Velez, L. L. Andersen, M. González-Izal, A. Jauregi and M. Izquierdo (2018). "Immediate Effects of Osteopathic Treatment Versus Therapeutic Exercise on Patients With Chronic Cervical Pain." Alternative Therapies in Health & Medicine **24**(3): 24-32.
Galindez-Ibarbengoetxea, X. S., Igor; Ramírez-Velez, Robinson; Andersen, Lars L.; González-Izal, Miriam; Jauregi, Andoni; Izquierdo, Mikel (2017). "Immediate Effects of Osteopathic Treatment Versus Therapeutic Exercise on Patients With Chronic Cervical Pain." Alternative Therapies in Health & Medicine **23**(7): 238-246.
Galindez-Ibarbengoetxea, X. S., Igor; Ramírez-Velez, Robinson; Andersen, Lars L.; González-Izal, Miriam; Jauregi, Andoni; Izquierdo, Mikel (2018). "Short-term effects of manipulative treatment versus a therapeutic home exercise protocol for chronic cervical pain: A randomized clinical trial." Journal of Back & Musculoskeletal Rehabilitation **31**(1): 133-145.

Gallego Izquierdo, T. P.-M., Daniel; Lluch Girbés, Enrique; Plaza-Manzano, Gustavo; Rodríguez Caldentey, Ricardo; Mayor Melús, Rodrigo; Blanco Mariscal, Diego; Falla, Deborah (2016). "COMPARISON OF CRANIO-CERVICAL FLEXION TRAINING VERSUS CERVICAL PROPRIOCEPTION TRAINING IN PATIENTS WITH CHRONIC NECK PAIN: A RANDOMIZED CONTROLLED CLINICAL TRIAL." Journal of Rehabilitation Medicine (Stiftelsen Rehabiliteringsinformation) **48**(1): 48-55.

Ganesh, G. S. M., Patitapaban; Pattnaik, Monalisa; Mishra, Chittaranjan (2015). "Effectiveness of mobilization therapy and exercises in mechanical neck pain." Physiotherapy Theory & Practice **31**(2): 99-106.

George, D. L. (2008). "Physical Exercise for Chronic Neck Pain." The Medscape Journal of Medicine **10**(4): 85.

Ghaderi, F. J., Mohammad Asghari; Javanshir, Khodabakhsh (2017). "The clinical and EMG assessment of the effects of stabilization exercise on nonspecific chronic neck pain: A randomized controlled trial." Journal of Back & Musculoskeletal Rehabilitation **30**(2): 211-219.

Ghafouri, N. G., Bijar; Fowler, Christopher J.; Larsson, Britt; Turkina, Maria V.; Karlsson, Linn; Gerdle, Björn (2014). "Effects of Two Different Specific Neck Exercise Interventions on Palmitoylethanolamide and Stearoylethanolamide Concentrations in the Interstitium of the Trapezius Muscle in Women with Chronic Neck Shoulder Pain." Pain Medicine **15**(8): 1379-1389.

Gialanella, B. E., Teresa; Faustini, Sabrina; Baratti, Doriana; Bernocchi, Palmira; Comini, Laura; Scalvini, Simonetta (2017). "Home-Based Telemedicine in Patients with Chronic Neck Pain." American Journal of Physical Medicine & Rehabilitation **96**(5): 327-332.

Giménez-Costa, M. (2018). "Effect of Therapeutic Exercise on the Activation of the Neck Extensors in People With Chronic Neck Pain." Clinical Trials.

Giombini, A. D. C., A.; Quaranta, F.; Giannini, S.; Di Cagno, A.; Mazzola, C.; Pigozzi, F.; Saraceni, V. M. (2013). "Neck balance system in the treatment of chronic mechanical neck pain: A prospective randomized controlled study." European Journal of Physical and Rehabilitation Medicine **49**(3): 283-290.

Gore, D., S. Sepic, G. Gardner and M. P. Murray (1987). "Neck pain: a long-term follow-up of 205 patients." Spine **12**(1).

Gram, B. A., Christoffer; Zebis, Mette K.; Bredahl, Thomas; Pedersen, Mogens T.; Mortensen, Ole S.; Jensen, Rigmor H.; Andersen, Lars L.; Sjøgaard, Gisela (2014). "Effect of training supervision on effectiveness of strength training for reducing neck/shoulder pain and headache in office workers: cluster randomized controlled trial." BioMed Research International **2014**: 693013-693013.

Griffiths, C. D., K.; Waterfield, J.; Sim, J. (2009). "Effectiveness of specific neck stabilization exercises or a general neck exercise program for chronic neck disorders: a randomized controlled trial." Journal of Rheumatology **36**(2): 390-397.

Groeneweg, R. v. A., Luite; Kropman, Hans; Leopold, Huco; Mulder, Jan; Smits-Engelsman, Bouwien C. M.; Ostelo, Raymond W. J. G.; Oostendorp, Rob A. B.; van Tulder, Maurits W. (2017). "Manual therapy compared with physical therapy in patients with non-specific neck pain: a randomized controlled trial." Chiropractic & Manual Therapies **25**: 1-12.

Ha, S. M., O. Kwon, F. Yi Ch, H. Jeon and W. H. Lee (2011). "Effects of passive correction of scapular position on pain, proprioception, and range of motion in neck-pain patients with bilateral scapular downward-rotation syndrome." Manual Therapy **16**(6).

Hagberg, M. H.-R., K.; Nisell, R.; Hjelm, E. W. (2000). "Rehabilitation of neck-shoulder pain in women industrial workers: a randomized trial comparing isometric shoulder endurance training with isometric shoulder strength training." Archives of Physical Medicine & Rehabilitation **81**(8): 1051-1058.

Hakkinen, A. K., H.; Hannonen, P.; Ylinen, J. (2008). "Strength training and stretching versus stretching only in the treatment of patients with chronic neck pain: a randomized one-year follow-up study [with consumer summary]." Clinical Rehabilitation 2008 Jul;22(7):592-600.

Helewa, A. G., C. H.; Smythe, H. A.; Lee, P.; Obright, K.; Stitt, L. (2007). "Effect of therapeutic exercise and sleeping neck support on patients with chronic neck pain: a randomized clinical trial." Journal of Rheumatology **34**(1): 151-158.

Highland, T. R., T. Dreisinger, L. Vie and G. S. Russell (1992). "Changes in isometric strength and range of motion of the isolated cervical spine after eight weeks of clinical rehabilitation." Spine **17**.

Hildreth, K. D. K. K. A. G. M. H. M. S. A. T. V. T.-L. J. (2015). "Comparative effectiveness of Pilates and yoga group exercise interventions for chronic mechanical neck pain: quasi-randomised parallel controlled study." Physiotherapy **102**(3): 236.

Hoving, J. L. d. V., H. C.; Koes, B. W.; Mameren, H.; Deville, W. L.; van der Windt, D. A.; Assendelft, W. J.; Pool, J. J.; Scholten, R. J.; Korthals-de Bos, I. B.; Bouter, L. M. (2006). "Manual therapy, physical therapy, or continued care by the general practitioner for patients with neck pain: long-term results from a pragmatic randomized clinical trial." The Clinical Journal of Pain 2006 May;22(4):370-377.

Hoving, J. L. K., Bart W.; de Vet, Henrica C. W.; van der Windt, Danielle A. W. M.; Assendelft, Willem J. J.; van Mameren, Henk; Devillé, Walter L. J. M.; Pool, Jan J. M.; Scholten, Rob J. P. M.; Bouter, Lex M. (2002). "Manual therapy, physical therapy, or continued care by a general practitioner for patients with neck pain. A randomized, controlled trial." Annals of internal medicine **136**(10): 713-722.

Hsieh, L.-F. (2012*). "Effect of Combined Neck Strength Exerciser Plus Physiotherapy to Treatment Chronic Neck Pain." Clinical Trials.

Iqbal, Z. A. (2018). "Efficacy of Deep Cervical Flexor Muscles Training on Neck Pain, Functional Disability and Muscle Endurance in School Teachers." Clinical Trials.

Iqbal, Z. A., R. Rajan, S. Khan and A. H. Alghadir (2013). "Effect of deep cervical flexor muscles training using pressure biofeedback on pain and disability of school teachers with neck pain." J Phys Ther Sci **25**.

Irfan, A., F. Sharif, F. Shaheen and A. Afzal (2018). "Effects of dynamic muscle strengthening and isometric muscle strengthening in treatment of mechanical neck pain; a randomized clinical trial." Rawal Medical Journal 2018 Jan;43(4):705-707.

Iversen, V. M., O. Vasseljen, P. J. Mork and M. S. Fimland (2018). "Resistance training vs general physical exercise in multidisciplinary rehabilitation of chronic neck pain: A randomized controlled trial." Journal of rehabilitation medicine **50**(8): 743-750.

Jakobsen, M. D. S., E.; Brandt, M.; Jay, K.; Aagaard, P.; Andersen, L. L. (2015). "Effect of workplace-versus home-based physical exercise on musculoskeletal pain among healthcare workers: A cluster randomized controlled trial." Scandinavian Journal of Work, Environment and Health **41**(2): 153-163.

Jamal, A. N. F., Brian M.; Pullenayegum, Eleanor (2016). "The Use of Neck Support Pillows and Postural Exercises in the Management of Chronic Neck Pain." Journal of Rheumatology **43**(10): 1871-1873.

Jay, K. S., Mc; Andersen, Christoffer H.; Ebbesen, Frederik S.; Christiansen, David H.; Skotte, Jørgen; Zebis, Mette K.; Andersen, Lars L. (2013). "Effect of brief daily resistance training on rapid force development in painful neck and shoulder muscles: randomized controlled trial." Clinical physiology and functional imaging **33**(5): 386-392.

Jensen, L. A., L. L.; Schrøder, H. D.; Frandsen, U.; Sjøgaard, G. (2015). "Neuronal Nitric Oxide Synthase Is Dislocated in Type I Fibers of Myalgic Muscle but Can Recover with Physical Exercise Training." BioMed Research International **2015**: 1-11.

Jensen, N. R. D. H. C. L. L. A. J. S. J. Y. O. K. J. C. V. N. C. (2014†). "Effect of strength training in addition to general exercise in patients on sick leave due to non-specific neck pain. A randomized clinical trial." European journal of physical and rehabilitation medicine **50**(6): 617.

Jordan, A. B., T.; Nielsen, H.; Hansen, F. R.; Host, D.; Winkel, A. (1998). "Intensive training, physiotherapy, or manipulation for patients with chronic neck pain: A prospective, single-blinded, randomized clinical trial." Spine **23**(3): 311-319.

JOSPT (2003). "May 2003 Abstracts." Journal of Orthopaedic & Sports Physical Therapy **33**(5): 287-294.

Journal of Alternative & Complementary Medicine (2016). "The International Congress on Integrative Medicine and Health (ICIMH)...Green Valley Ranch Resort, Las Vegas, Nevada, USA May 17–20, 2016." Journal of Alternative & Complementary Medicine **22**(6).

Jull, G. A. F., D.; Vicenzino, B.; Hodges, P. W. (2009). "The effect of therapeutic exercise on activation of the deep cervical flexor muscles in people with chronic neck pain." Manual Therapy **14**(6): 696-701.

Jull, G. F., D.; Treleaven, J.; Hodges, P.; Vicenzino, B. (2007). "Retraining cervical joint position sense: The effect of two exercise regimes." Journal of Orthopaedic Research **25**(3): 404-412.

Kaka, B. O., Omoyemi O.; Adeniyi, Ade F.; Maharaj, Sonill S.; Ogunlade, Samuel O.; Bello, Bashir (2018). "Effectiveness of neck stabilisation and dynamic exercises on pain intensity, depression and anxiety among patients with non-specific neck pain: a randomised controlled trial." Scandinavian journal of pain **18**(2): 321-331.

Kaminski, C. (2011). "Effect of active and passive movement versus pure active exercise in non-specific neck pain." Manuelle Therapie **15**(3): 117-123.

Karlsson, L. G., B.; Ghafouri, B.; Bckryd, E.; Olausson, P.; Ghafouri, N.; Larsson, B. (2015). "Intramuscular pain modulatory substances before and after exercise in women with chronic neck pain." European Journal of Pain **19, NUMB 8**: 1075-1085.

Karlsson, L. T., E. P.; Gerdle, B.; Larsson, B. (2014). "Evaluation of pain and function after two home exercise programs in a clinical trial on women with chronic neck pain -- with special emphasises on completers and responders." BMC Musculoskeletal Disorders 2014 Jan 8;15(6):Epub.

Karlsson, L. T., Esa-Pekka; Gerdle, Björn; Larsson, Britt (2014). "Evaluation of pain and function after two home exercise programs in a clinical trial on women with chronic neck pain - with special emphasises on completers and responders." BMC Musculoskeletal Disorders **15**(1): 6-6.

Kashfi, P., N. Karimi, A. Peolsson and L. Rahnama (2019). "The effects of deep neck muscle-specific training versus general exercises on deep neck muscle thickness, pain and disability in patients with chronic non-specific neck pain: protocol for a randomized clinical trial (RCT)." BMC Musculoskeletal Disorders **20**(1).

Kawarada, K. (2016). "Effect of Cranio-cervical Flexion Self-Exercise Using a Special Device on Subjective and Objective Outcomes of Neck Pain." Rigakuryoho kagaku **31, NUMB 1**: 107-110.

Kietrys, D. M. G., J. S.; Verno, V. (2007). "Effects of at-work exercises on computer operators." Work **28**(1): 67-75.

Kim, S. D. (2018). "Effects of yogic exercise on nonspecific neck pain in university students." Complementary Therapies in Clinical Practice **31**: 338-342.

Kjellman, G. Ö., B. (2002). "A randomized clinical trial comparing general exercise, McKenzie treatment and a control group in patients with neck pain." Journal of Rehabilitation Medicine (Taylor & Francis Ltd) **34**(4): 183-190.

Klemetti, M. S., N.; Sarvimaki, A.; Bjorvell, H. (1997). "Tension neck and evaluation of a physical training course among office workers in a bank corporation." Journal of advanced nursing **26**(5): 962-967.

Lange, B. T., P.; Myburgh, C.; Sjøgaard, G. (2013). "Effect of Targeted Strength, Endurance, and Coordination Exercise on Neck and Shoulder Pain Among Fighter Pilots: A Randomized-controlled Trial." Clinical Journal of Pain **29**(1): 50-59.

Lansinger, B. C., Jane Y.; Kreuter, Margareta; Taft, Charles (2013). "Health-related quality of life in persons with long-term neck pain after treatment with qigong and exercise therapy respectively." European Journal of Physiotherapy **15**(3): 111-117.
Lansinger, B. L., E.; Persson, L. C.; Carlsson, J. Y. (2007). "Qigong and Exercise Therapy in Patients With Long-term Neck Pain : A Prospective Randomized Trial." Spine **32, NUMB 22**: 2415-2422.
Lansinger, B. L., E.; Persson, L.; Carlsson, J. Y. (2007). "Qigong and exercise therapy in patients with long-term neck pain: a prospective randomized trial [with consumer summary]." Spine 2007 Oct 15;32(22):2415-2422.
Lansinger, V. L., E.; Persson, L. C.; Carlsson, J. Y. (2007). "Qigong and experience therapy in patients with long-term neck pain: a prospective randomized trial." Spine (03622436) **32**(22): 2415-2422.
Lauche, R. S., Christoph; Fehr, Johannes; Cramer, Holger; Cheng, Ying Wu; Wayne, Peter M.; Rampp, Thomas; Langhorst, Jost; Dobos, Gustav (2016). "The Effects of Tai Chi and Neck Exercises in the Treatment of Chronic Nonspecific Neck Pain: A Randomized Controlled Trial." Journal of Pain **17**(9): 1013-1027.
Lauche, R. W., Peter M.; Fehr, Johannes; Stumpe, Christoph; Dobos, Gustav; Cramer, Holger (2017). "Does Postural Awareness Contribute to Exercise-Induced Improvements in Neck Pain Intensity? A Secondary Analysis of a Randomized Controlled Trial Evaluating Tai Chi and Neck Exercises." Spine (03622436) **42**(16): 1195-1200.
Lawrence, D. J. D., K. (2005). "Manual therapy or pulsed shortwave diathermy provide no additional benefit over advice and exercise in the treatment of neck disorders." Focus on Alternative & Complementary Therapies **10**(4): 307-308.
Leininger, B. M., C.; Evans, R.; Tosteson, T.; Tosteson, A. N. A.; Bronfort, G. (2016). "Cost-effectiveness of spinal manipulative therapy, supervised exercise, and home exercise for older adults with chronic neck pain [with consumer summary]." The Spine Journal 2016 Nov;16(11):1292-1304.
Leininger, B. M., Christine; Evans, Roni; Tosteson, Tor; Tosteson, Anna N. A.; Bronfort, Gert (2016). "Cost-effectiveness of spinal manipulative therapy, supervised exercise, and home exercise for older adults with chronic neck pain." Spine Journal **16**(11): 1292-1304.
Levoska, S. and S. Keinanen-Kiukaanniemi (1993). "Active or passive physiotherapy for occupational cervicobrachial disorders? A comparison of two treatment methods with a 1-year follow-up." Archives of Physical Medicine & Rehabilitation **74**.
Lin, I. H. C., K. H.; Liou, T. H.; Tsou, C. M.; Huang, Y. C. (2018). "The effect of progressive shoulder-neck exercise on cervical muscle functions of middle-aged and senior patients with chronic neck pain: a randomized controlled trial." European Journal of Physical and Rehabilitation Medicine 2018 Feb;54(1):13-21.
Lin, I. H. C., K. H.; Liou, T. H.; Tsou, C. M.; Huang, Y. C. (2018). "Progressive shoulder-neck exercise on cervical muscle functions in middle-aged and senior patients with chronic neck pain." European journal of physical and rehabilitation medicine **54, NUMB 1**: 13-21.
Lluch, E. A., M. D.; Coloma, P. S.; Palma, F.; Rey, A.; Falla, D. (2013). "Effects of deep cervical flexor training on pressure pain thresholds over myofascial trigger points in patients with chronic neck pain." J Manipulative Physiol Ther **36**(9): 604-611.
Lluch, E. A., Maria Dolores; Calvente Quesada, Otilia; Martínez Noguera, Estibaliz; Peiró Puchades, Marta; Pérez Rodríguez, José A.; Falla, Deborah (2014). "IMMEDIATE EFFECTS OF A CTIVE VERSUS PASSIVE SCAPULAR CORRECTION ON PAIN AND PRESSURE PAIN THRESHOLD IN PATIENTS WITH CHRONIC NECK PAIN." Journal of Manipulative & Physiological Therapeutics **37**(9): 660-666.
Lluch, E. S., Jochen; Gizzi, Leonardo; Petzke, Frank; Seegar, Dagmar; Falla, Deborah (2014). "Immediate effects of active cranio-cervical flexion exercise versus passive mobilisation of the upper cervical spine on pain and performance on the cranio-cervical flexion test." Manual Therapy **19**(1): 25-31.
Lundqvist, L.-O. Z., Christina; Richter, Hans O. (2014). "Effects of Feldenkrais method on chronic neck/scapular pain in people with visual impairment: a randomized controlled trial with one-year follow-up." Archives of physical medicine and rehabilitation **95**(9): 1656-1661.
Ma, C. S., Grace P.; Yan, Tiebin; Wu, Shaoling; Lin, Caina; Li, Lijuan (2011). "Comparing Biofeedback With Active Exercise and Passive Treatment for the Management of Work-Related Neck and Shoulder Pain: A Randomized Controlled Trial." Archives of Physical Medicine & Rehabilitation **92**(6): 849-858.
Maiers, M. B., G.; Evans, R.; Hartvigsen, J.; Svendsen, K.; Bracha, Y.; Schulz, C.; Schulz, K.; Grimm, R. (2014). "Spinal manipulative therapy and exercise for seniors with chronic neck pain." Spine Journal **14**(9): 1879-1889.
Maiers, M. B., G.; Evans, R.; Hartvigsen, J.; Svendsen, K.; Bracha, Y.; Schulz, C.; Schulz, K.; Grimm, R. (2014). "Spinal manipulative therapy and exercise for seniors with chronic neck pain [with consumer summary]." The Spine Journal 2014 Sep;14(9):1879-1889.
Manca, A. D., J. C.; Torgerson, D. J.; Klaber Moffett, J. A.; Mooney, M. P.; Jackson, D. A.; Eaton, S. (2007). "Randomized trial of two physiotherapy interventions for primary care back and neck pain patients: cost effectiveness analysis [with consumer summary]." Rheumatology 2007 Sep;46(9):1495-1501.
Marco, M. E., Ambrosini; Barbara, Rocca; Daniele, Cazzaniga; Valentina, Liquori; Alessandra, Pedrocchi; Howard, Vernon (2016). "Group-based multimodal exercises integrated with cognitive-behavioural therapy improve disability, pain and quality of life of subjects with chronic neck pain: A randomized controlled trial with one-year follow-up." Clinical rehabilitation **31**(6): 742.
Martel, J. D., C.; Dubois, J. D.; Descarreaux, M. (2011). "A randomised controlled trial of preventive spinal manipulation with and without a home exercise program for patients with chronic neck pain [randomized controlled trial]." BMC Musculoskel Disord **12**(1): Online access only 36 p.
Martel, J. D., Claude; Dubois, Jean-Daniel; Descarreaux, Martin (2011). "A randomised controlled trial of preventive spinal manipulation with and without a home exercise program for patients with chronic neck pain." BMC Musculoskeletal Disorders **12**(1): 41-41.
McLean, S. M. (2007). Conservative management of non-specific neck pain : effectiveness of treatment, predictors of treatment outcome and upper limb disability, University of Hull ;.
McLean, S. M. K. M., Jennifer A.; Sharp, Donald M.; Gardiner, Eric (2013). "A randomised controlled trial comparing graded exercise treatment and usual physiotherapy for patients with non-specific neck pain (the GET UP neck pain trial)." Manual therapy **18**(3): 199-205.
Mohammadi, M. R., Asghar; Zavieh, Minoo Khalkhali; Tabatabaie, Seyed Mahdi; Rezaie, Mahdi (2014). "The Effect of Shoulder Resistance Training on Isometric Strength of the Neck Extensor Muscles." Qom University of Medical Sciences Journal **8**(4): 10-12.
Monticone, M. A., Emilia; Rocca, Barbara; Cazzaniga, Daniele; Liquori, Valentina; Pedrocchi, Alessandra; Vernon, Howard (2017). "Group-based multimodal exercises integrated with cognitive-behavioural therapy improve disability, pain and quality of life of subjects with chronic neck pain: a randomized controlled trial with one-year follow-up." Clinical Rehabilitation **31**(6): 742-752.
Mortensen, P. L., Anders I.; Zebis, Mette K.; Pedersen, Mogens T.; Sjøgaard, Gisela; Andersen, Lars L. (2014). "Lasting effects of workplace strength training for neck/shoulder/arm pain among laboratory technicians: natural experiment with 3-year follow-up." BioMed research international **2014**: 845851.
Murray, M. L., Britt; Nørnberg, Bo Riebeling; Søgaard, Karen; Sjøgaard, Gisela (2017). "Self-administered physical exercise training as treatment of neck and shoulder pain among military helicopter pilots and crew: a randomized controlled trial." BMC Musculoskeletal Disorders **18**: 1-11.
Myhre, K. M., Gunn Hege; Leivseth, Gunnar; Keller, Anne; Bautz-Holter, Erik; Sandvik, Leiv; Lau, Bjørn; Røe, Cecilie (2014). "The Effect of Work-Focused Rehabilitation Among Patients With Neck and Back Pain: A Randomized Controlled Trial." Spine **39**(24): 1999-2006.
Nadler, S. F. Y., J.; Nadler, Scott F. (2003). "Isometric training to treat chronic neck pain...Ylinen J, Takala EP, Nykänen M et al. Active neck muscle training in the treatment of chronic neck pain in women: a randomized controlled trial. JAMA. 2003;289:2509-2516." JAMA: Journal of the American Medical Association **290**(8): 1027-1028.
Nazari, G., P. Bobos, E. Billis and J. C. MacDermid (2018). "Cervical flexor muscle training reduces pain, anxiety, and depression levels in patients with chronic neck pain by a clinically important amount: A prospective cohort study." Physiotherapy Research International **23**(3): 1-6.
Nezamuddin, M. A., S.; Khan, S. A.; Equebal, A. (2013). "Efficacy of pressure-biofeedback guided deep cervical flexor training on neck pain and muscle performance in visual display terminal operators." Journal of Musculoskeletal Research 2013 Sep;16(3):1350011.
Nezamuddin, M. K., Sohrab A.; Hameed, Unaise A.; Anwer, Shahnawaz; Equebal, Ameed (2013). "Efficacy of Pressure Biofeedback Guided Deep Cervical Flexor Training on Forward Head Posture in Visual Display Terminal Operators." Indian Journal of Physiotherapy & Occupational Therapy **7**(4): 141-146.
Nielsen, P. K. A., L. L.; Olsen, H. B.; Rosendal, L.; Sjøgaard, G.; Søgaard, K.; Nielsen, Pernille Kofoed; Andersen, Lars L.; Olsen, Henrik B.; Rosendal, Lars; Sjøgaard, Gisela; Søgaard, Karen (2010). "Effect of physical training on pain sensitivity and trapezius muscle morphology." Muscle & Nerve **41**(6): 836-844.
O'Leary, S. F., D.; Hodges, P. W.; Jull, G.; Vicenzino, B. (2007). "Specific therapeutic exercise of the neck induces immediate local hypoalgesia [with consumer summary]." The Journal of Pain 2007 Nov;8(11):832-839.
O'Leary, S. J., G.; Kim, M.; Vicenzino, B. (2007). "Specificity in retraining craniocervical flexor muscle performance." Journal of Orthopaedic & Sports Physical Therapy **37**(1): 3-9.
O'Leary, S. J., Gwendolen; Kim, Mehwa; Uthaikhup, Sureeporn; Vicenzino, Bill (2012). "Training mode-dependent changes in motor performance in neck pain." Archives of physical medicine and rehabilitation **93**(7): 1225-1233.
O'Leary, S. J., Gwendolen; Kim, Mehwa; Uthaikhup, Sureeporn; Vicenzino, Bill (2012). "Training Mode–Dependent Changes in Motor Performance in Neck Pain." Archives of Physical Medicine & Rehabilitation **93**(7): 1225-1233.
O Leary, S. F., D.; Jull, G. (2003). "Recent Advances In Therapeutic Exercise For The Neck : Implications For Patients With Head And Neck Pain." Australian Endodontic Journal **29, PART 3**: 138-142.
Oda, K. Y. (2003). "Posterior; approach; to; the; degenerative; cervical; spine." European; Spine; Journal **12**(2).
Oldervoll, L. M. R., M.; Zwart, J. A.; Svebak, S. (2001). "Comparison of two physical exercise programs for the early intervention of pain in the neck, shoulders and lower back in female hospital staff." Journal of Rehabilitation Medicine **33**(4): 156-161.
OsteloM.;, J. M. v. D. G. M. R. E. B. A. B. O. W. J. G. (2016). "Cost-effectiveness; of; manual; therapy; versus; physiotherapy; in; patients; with; sub-acute; and; chronic; neck; pain:; a; randomised; controlled; trial." European; Spine; Journal **25**(7).
Paoloni, M. T., E.; Cacchio, A.; Tattoli, M.; Melis, L.; Ronconi, R.; Santilli, V. (2013). "Patient-oriented rehabilitation in the management of chronic mechanical neck pain: A randomized controlled trial." European Journal of Physical and Rehabilitation Medicine **49**(3): 273-281.
Park, D. S. L., G. C. (2006). "PR_197 : The Effect of Strengthening Exercise of Deep Neck Flexors on Chronic Neck Pain." Archives of Physical Medicine and Rehabilitation **87, NUMB 11**: e38.

Park, K. N. K., S. H. (2016). "The effects of functional postural training versus cervicoscapular muscle training in violinists with chronic neck pain." Manual Therapy **25**: e99.
Pedersen, M. T. A., Christoffer H.; Zebis, Mette K.; Sjøgaard, Gisela; Andersen, Lars L. (2013). "Implementation of specific strength training among industrial laboratory technicians: long-term effects on back, neck and upper extremity pain." BMC Musculoskeletal Disorders **14**(1): 287-287.
Pedersen, M. T. A., Lars L.; Jørgensen, Marie B.; Sogaard, Karen; Sjøgaard, Gisela (2013†). "EFFECT OF SPECIFIC RESISTANCE TRAINING ON MUSCULOSKELETAL PAIN SYMPTOMS: DOSE-RESPONSE RELATIONSHIP." Journal of Strength & Conditioning Research (Lippincott Williams & Wilkins) **27**(1): 229-235.
Pereira, M., T. Comans, G. Sjogaard, L. Straker, M. Melloh, S. O'Leary, X. Chen and V. Johnston (2019). "The impact of workplace ergonomics and neck-specific exercise versus ergonomics and health promotion interventions on office worker productivity: a cluster-randomized trial." Scandinavian Journal of Work, Environment & Health 2019 Jan;45(1):42-52.
Personnal Today (2004). "Hospitals to trial alternative to neck physiotherapy." Occupational Health **56**(5): 7-7.
Petrofsky, J. S. L., Michael; Alshammari, Faris; Khowailed, Iman Akef; Haneul, Lee (2017). "Use of low level of continuous heat and Ibuprofen as an adjunct to physical therapy improves pain relief, range of motion and the compliance for home exercise in patients with nonspecific neck pain: A randomized controlled trial." Journal of Back & Musculoskeletal Rehabilitation **30**(4): 889-896.
Pillastrini, P. M., Raffaele; Bertozzi, Lucia; Costi, Stefania; Curti, Stefania; Mattioli, Stefano; Violante, Francesco Saverio (2009). "Effectiveness of an at-work exercise program in the prevention and management of neck and low back complaints in nursery school teachers." Industrial health **47**(4): 349-354.
Raja, R. K., K.; Anandh, V. (2015). "The Effects of Thoracic Thrust Manipulation and Neck Flexibility Exercises for the Management of the Patients with Mechanical Neck Pain." Indian Journal of Physiotherapy & Occupational Therapy **9**(4): 168-172.
Rajalaxmi, V. J., A.; Sudhakar, S.; Mohan Kumar, G. (2018). "To Analyse the Effectiveness of Yoga, Pilates and Tai Chi Exercise for Chronic Mechanical Neck Pain- A Randomized Controlled Trial." Biomedicine **38, NUMB 1**: 147-151.
Raju, A. S., P. Apparao, G. Swamy, P. Chaturvadi and R. G. Mounika (2019). "A Comparative Study on Deep Cervical Flexors Training and Neck Stabilization Exercises in Subjects with Chronic Neck Pain." Indian Journal of Physiotherapy & Occupational Therapy **13**(2): 1-5.
Rasotto, C. B., M.; Sieverdes, J. C.; Gobbo, S.; Alberton, C. L.; Neunhaeuserer, D.; Maso, S.; Zaccaria, M.; Ermolao, A. (2015). "A Tailored Workplace Exercise Program for Women at Risk for Neck and Upper Limb Musculoskeletal Disorders: A Randomized Controlled Trial." Journal of Occupational and Environmental Medicine **57**(2): 178-183.
Rasotto, C. B., M.; Simonetti, A.; Maso, S.; Bartolucci, G. B.; Ermolao, A.; Zaccaria, M. (2015). "Tailored exercise program reduces symptoms of upper limb work-related musculoskeletal disorders in a group of metalworkers: A randomized controlled trial." Manual Therapy **20**(1): 56-62.
Rasotto, C. B., Marco; Sieverdes, John C.; Gobbo, Stefano; Alberton, Cristine L.; Neunhaeuserer, Daniel; Maso, Stefano; Zaccaria, Marco; Ermolao, Andrea (2015). "A Tailored Workplace Exercise Program for Women at Risk for Neck and Upper Limb Musculoskeletal Disorders." Journal of Occupational & Environmental Medicine **57**(2): 178-183.
Ratzon, N. Z. B.-N., Netta Abraham; Froom, Paul (2016). "The effect of a structured personalized ergonomic intervention program for hospital nurses with reported musculoskeletal pain: An assigned randomized control trial." Work **54**(2): 367-377.
Ravi, C. N. D., Bid Dibyendunarayan; A, Thangamani Ramalingam (2016). "Effectiveness of Proprioceptive Exercises in Chronic Nonspecific Neck Pain Patients." Indian Journal of Physiotherapy & Occupational Therapy **10**(4): 143-148.
Reinikka, K. J. E. F., E.; MacLeod, A. (2009). "Physiotherapists: facilitating partnerships for sustainable community exercise programmes for people with chronic conditions." Physiotherapy Canada **61**: 24-24.
Rendant, D. P., D.; Ludtke, R.; Reisshauer, A.; Mietzner, A.; Willich, S. N.; Witt, C. M. (2011). "Qigong versus exercise versus no therapy for patients with chronic neck pain: a randomized controlled trial [with consumer summary]." Spine 2011 Mar 15;36(6):419-427.
Rendant, D. P., D.; Lüdtke, R.; Reisshauer, A.; Mietzner, A.; Willich, S. N.; Witt, C. M. (2011). "Qigong versus exercise versus no therapy for patients with chronic neck pain: a randomized controlled trial." Spine (03622436) **36**(6): 419-427.
Riveiro, D. V. (2003). "Chronic neck pain and exercise." Rehabilitacion **37, PART 6**: 333-338.
Rolving, N. C., D. H.; Andersen, L. L.; Skotte, J.; Ylinen, J.; Jensen, O. K.; Nielsen, C. V.; Jensen, C. (2014). "Effect of strength training in addition to general exercise in the rehabilitation of patients with non-specific neck pain. A randomized clinical trial." European Journal of Physical & Rehabilitation Medicine **50**(6): 617-626.
Rothstein, J. M. (2001). "PT 2001: the Annual Conference and Exposition of the APTA abstracts of papers accepted for presentation." Physical Therapy **81**(5).
Salo, P. K. H., A. H.; Kautiainen, H.; Ylinen, J. J.; Salo, Petri K.; Häkkinen, Arja H.; Kautiainen, Hannu; Ylinen, Jari J. (2010). "Effect of neck strength training on health-related quality of life in females with chronic neck pain: a randomized controlled 1-year follow-up study." Health & Quality of Life Outcomes **8**: 48-48.
Salo, P. Y.-K., N.; Hakkinen, A.; Kautiainen, H.; Malkia, E.; Ylinen, J. (2012). "Effects of long-term home-based exercise on health-related quality of life in patients with chronic neck pain: a randomized study with a 1-year follow-up [with consumer summary]." Disability and Rehabilitation 2012;34(23):1971-1977.
Salo, P. Y.-K., Niina; Häkkinen, Arja; Kautiainen, Hannu; Mälki, Esko; Ylinen, Jari (2012). "Effects of long-term home-based exercise on health-related quality of life in patients with chronic neck pain: A randomized study with a 1-year follow-up." Disability & Rehabilitation **34**(23): 1971-1977.
Sarig Bahat, H. T., Hiroshi; Chen, Xiaoqi; Bet-Or, Yaheli; Treleaven, Julia (2015). "Cervical kinematic training with and without interactive VR training for chronic neck pain - a randomized clinical trial." Manual therapy **20**(1): 68-78.
Savolainen, A. A., Jari; Nummila, Hanna; Nissinen, Maunu (2004†). "Active or passive treatment for neck-shoulder pain in occupational health care? A randomized controlled trial." Occupational medicine (Oxford, England) **54**(6): 422-424.
Scheel, I. B. (2003). "Controlled endurance or strength training of the neck muscles decreases pain and disability in women with chronic neck pain." Australian Journal of Physiotherapy **49**(3): 221-221.
Scheel, I. B. (2003). "Controlled endurance or strength training of the neck muscles decreases pain and disability in women with chronic neck pain: Commentary." Australian Journal of Physiotherapy **49**(3): 221.
Senthil, P. S., S.; Radhakrishnan, R. (2016). "Isolated Activation of Deep Cervical Flexor Muscles to Improve the Functional Outcome of Subjects with Cervical Dysfunction." Indian Journal of Physiotherapy & Occupational Therapy **10**(2): 121-124.
Shariat, A. L., Eddie T. C.; Kargarfard, Mehdi; Tamrin, Shamsul B. M.; Danaee, Mahmoud (2017). "The application of a feasible exercise training program in the office setting." Work **56**(3): 421-428.
Sharmila, B. (2014). "Isometric Muscle Energy Technique and Non-Specific Neck Pain in Secondary School Teachers -- Results of an Experimental Study." Indian Journal of Physiotherapy & Occupational Therapy **8**(2): 58-62.
Shenoy, S. S., J.; Sandhu, J. S. (2010). "Effectiveness of strengthening exercises in the management of forward head posture among computer professionals." Indian Journal of Physiotherapy & Occupational Therapy **4**(3): 37-41.
Shih, Y. L., K.; Lin, H.; Kao, Y. (2008). "P017 Effect of exercise training on dynamic posture performance of the head, neck, and shoulder in subjects with chronic neck pain." Gait and Posture **28, SUPP 2**: S59.
Silva, A. G. (2018). "Exercise and Pain Neuroscience Education for Patients With Neck Pain: Impact on Pain and Disability." Clinical Trials.
Sionnadh, M. M. J., A. Klaber Moffett; Donald, M. Sharp; Eric, Gardiner (2013). "A randomised controlled trial comparing graded exercise treatment andÂ usualÂ physiotherapy for patients with non-specific neck pain (the GET UP neck pain trial)." Manual therapy **18**(3): 199.
Sjøgaard, G. (2013). "Exercise as Treatment of Neck Pain Among Helicopter Pilots and Crew Members." Clinical Trials.
Sjögren, T. N., Kari J.; Järvenpää, Salme K.; Ojanen, Markku T.; Vanharanta, Heikki; Mälkiä, Esko A. (2005). "Effects of a workplace physical exercise intervention on the intensity of headache and neck and shoulder symptoms and upper extremity muscular strength of office workers: a cluster randomized controlled cross-over trial." Pain (03043959) **116**(1/2): 119-128.
Skelly, D. L. (2016). "Sub-clinical Neck Symptoms, Disability, Posture, and Muscle Function in Computer Users, and the Effect of Education versus Education and Deep Cervical Flexor Exercise." Sub-clinical Neck Symptoms, Disability, Posture & Muscle Function in Computer Users & the Effect of Education versus Education & Deep Cervical Flexor Exercise: 1-1.
Søgaard, K. B., A. K.; Nielsen, P. K.; Hansen, L.; Andersen, L. L.; Vedsted, P.; Sjøgaard, G. (2012). "Changed activation, oxygenation, and pain response of chronically painful muscles to repetitive work after training interventions: a randomized controlled trial." European Journal of Applied Physiology **112**(1): 173-181.
Spine (2009). "Online Article Abstracts." Spine **34**(16): A11-A13.
Stenchever, M. A. (2003). "Neck muscle training for chronic neck pain." ACOG Clinical Review **8**(9): 15-15.
Suni, J. H. R., M.; Tokola, K.; Manttari, A.; Vasankari, T. (2017). "Effectiveness of a standardised exercise programme for recurrent neck and low back pain: a multicentre, randomised, two-arm, parallel group trial across 34 fitness clubs in Finland [with consumer summary]." BMJ Open Sport & Exercise Medicine 2017 Apr;3(1):e000233.
Taheri, H. M., R.; Minasian, V.; Karimi, A. (2012). "The effects of an eight-week selected therapeutic exercises course and self-treatment by pamphlet programs on the rate of chronic neck pain and disability among computer users." Journal of Isfahan Medical School **29**(169).
Taimela, S. T., E.; Asklöf, T.; Seppälä, K.; Parviainen, S. (2000). "Active treatment of chronic neck pain: a prospective randomized intervention." Spine (03622436) **25**(8): 1021-1027.
Telci, E. A. K., A. (2012). "Effects of three different conservative treatments on pain, disability, quality of life, and mood in patients with cervical spondylosis." Rheumatology International **32**(4): 1033-1040.
Thompson, D. P. O., J. A.; Woby, S. R. (2016). "Does adding cognitive-behavioural physiotherapy to exercise improve outcome in patients with chronic neck pain? A randomised controlled trial." Physiotherapy **102**(2): 170-177.
Thompson, D. P. W., Steve R. (2018). "The processes underpinning reductions in disability among people with chronic neck pain. A preliminary comparison between two distinct types of physiotherapy intervention." Disability & Rehabilitation **40**(7): 779-783.
Treleaven, H. S. B. C. C. H. S. (2018). "Remote; kinematic; training; for; patients; with; chronic; neck; pain:; a; randomised; controlled; trial." European; Spine; Journal **27**(6).
Tsauo, J. L., H.; Hsu, J.; Chen, C. (2004). "Physical exercise and health education for neck and shoulder complaints among sedentary workers." Journal of Rehabilitation Medicine 2004 Nov;36(6):253-257.

Van Dillen, L. R., M. McDonnell, T. Susco and S. A. Sahrmann (2007). "The immediate effect of passive scapular elevation on symptoms with active neck rotation in patients with neck pain." Clinical Journal of Pain **23**(8).
Vasseljen, O., Jr.; Johansen, B. M.; Westgaard, R. H. (1995). "The effect of pain reduction on perceived tension and EMG-recorded trapezius muscle activity in workers with shoulder and neck pain." Scandinavian Journal of Rehabilitation Medicine 1995 Dec;27(4):243-252.
Vihstadt, C. M., M.; Westrom, K.; Bronfort, G.; Evans, R.; Hartvigsen, J.; Schulz, C. (2014). "Short term treatment versus long term management of neck and back disability in older adults utilizing spinal manipulative therapy and supervised exercise: A parallel-group randomized clinical trial evaluating relative effectiveness and harms." Chiropractic and Manual Therapies **22**(1): 26.
Vihstadt, C. M., M.; Westrom, K.; Bronfort, G.; Evans, R.; Hartvigsen, J.; Schulz, C. (2014). "Short term treatment versus long term management of neck and back disability in older adults utilizing spinal manipulative therapy and supervised exercise: A parallel-group randomized clinical trial evaluating relative effectiveness and harms [randomized controlled trial]." Chiropr & Manual Ther **22**(26): Online access only 15 p.
Viljanen, M. M., A.; Uitti, J.; Rinne, M.; Palmroos, P.; Laippala, P. (2003). "Effectiveness of dynamic muscle training, relaxation training, or ordinary activity for chronic neck pain: randomised controlled trial [with consumer summary]." BMJ 2003 Aug 30;327(7413):475-479.
von Trott, P. W., A. M.; Ludtke, R.; Reishauer, A.; Willich, S. N.; Witt, C. M. (2009). "Qigong and exercise therapy for elderly patients with chronic neck pain (QIBANE): a randomized controlled study [with consumer summary]." The Journal of Pain 2009 May;10(5):501-508.
von Trott, P. W., A. M.; Lüdtke, R.; Reishauer, A.; Willich, S. N.; Witt, C. M. (2009). "Qigong and exercise therapy for elderly patients with chronic neck pain (QIBANE): a randomized controlled study." Journal of Pain **10**(5): 501-508.
Vonk, F. V., A. P.; Twisk, J. W.; Köke, A. J.; Luiten, M. W.; Koes, B. W. (2009). "Effectiveness of a behaviour graded activity program versus conventional exercise for chronic neck pain patients." European Journal of Pain **13**(5): 533-541.
Waling, K. J., B.; Sundelin, G. (2002). "Effects of training on female trapezius myalgia: An intervention study with a 3-year follow-up period." Spine **27**(8): 789-796.
Waling, K. J., B.; Sundelin, G. (2002). "Effects of training on female trapezius myalgia: an intervention study with a 3-year follow-up period [with consumer summary]." Spine 2002 Apr 15;27(8):789-796.
Wang, W. T., S. Olson, A. Campbell, W. Hanten and P. B. Gleeson (2003). "Effectiveness of physical therapy for patients with neck pain: an individualized approach using a clinical decision-making algorithm." American Journal of Physical Medicine & Rehabilitation **82**(3).
Wani, S. R., Neha; Jethwa, Juhi; Mohammed, Rafi (2013). "Comparative efficacy of cervical retraction exercises (McKenzie) with and without using pressure biofeedback in cervical spondylosis." International Journal of Therapy & Rehabilitation **20**(10): 501-508.
Westaway, M. (2003). "Manual therapy, physical therapy, or care by a general practitioner for neck pain." Clinical Journal of Sport Medicine **13**(3): 197.
Wilson, S. (2001). "Spinal manipulation and exercise for chronic neck pain: Are they more effective when delivered alone or in combination?" Australian Journal of Physiotherapy **47**(4): 300.
Witt, C. M. (2008). "Qigong and Exercise for Neck Pain in Adults (QENA)." Clinical Trials.
Woby, D. P. T. J. A. O. S. R. (2015). "Does adding cognitive-behavioural physiotherapy to exercise improve outcome in patients with chronic neck pain? A randomised controlled trial." Physiotherapy **102**(2): 170.
Yelland, M. (2003). "Both endurance training and strength training reduced disability and pain in chronic nonspecific neck pain in women." ACP Journal Club **139**(3): 75-75.
Yildiz, T. I. (2018). "Scapular Stabilization Exercise on Neck Pain." Clinical Trials.
Yildiz, T. I., E. Turgut and I. Duzgun (2018). "Neck and Scapula-Focused Exercise Training on Patients With Nonspecific Neck Pain: A Randomized Controlled Trial." Journal of Sport Rehabilitation **27**(5): 403-412.
Ylinen, J. (2010). "Effect of Neck Strength Training on Health-related Quality of Life in Females With Neck Pain." Clinical Trials.
Ylinen, J. J. H., A. H.; Takala, E. P.; Nykanen, M. J.; Kautiainen, H. J.; Malkia, E. A.; Pohjolainen, T. H.; Karppi, S. L.; Airaksinen, O. V. (2006). "Effects of neck muscle training in women with chronic neck pain: one-year follow-up study [with consumer summary]." Journal of Strength & Conditioning Research 2006 Feb;20(1):6-13.
Ylinen, J. J. T., E. P.; Nykänen, M. J.; Kautiainen, H. J.; Häkkinen, A. H.; Airaksinen, O. V. P. (2006). "Effects of twelve-month strength training subsequent to twelve-month stretching exercise in treatment of chronic neck pain." Journal of Strength & Conditioning Research **20**(2): 304-308.
Ylinen, J. T., E. P.; Nykanen, M.; Hakkinen, A.; Kautiainen, H.; Malkia, E.; Pohjolainen, T.; Karppi, S. L.; Airaksinen, O. (2004). "Exercise of neck and shoulder muscles as a relief for the chronic neck pain." Duodecim; laaketieteellinen aikakauskirja **120**(16): 1958-1967.
Ylinen, J. T., Esa-Pekka; Kautiainen, Hannu; Nykänen, Matti; Häkkinen, Arja; Pohjolainen, Timo; Karppi, Sirkka-Liisa; Airaksinen, Olavi (2005). "Effect of long-term neck muscle training on pressure pain threshold: a randomized controlled trial." European journal of pain (London, England) **9**(6): 673-681.
Zaproudina, N. H., O. O. P.; Airaksinen, O. (2007). "Effectiveness of traditional bone setting in chronic neck pain: randomized clinical trial." Journal of Manipulative & Physiological Therapeutics **30**(6): 432-437.
Zaproudina, N. H., O. O.; Airaksinen, O. (2007). "Effectiveness of traditional bone setting in chronic neck pain: randomized clinical trial [with consumer summary]." Journal of Manipulative and Physiological Therapeutics 2007 Jul-Aug;30(6):432-437.
Zebis, M. K. A., Christoffer H.; Sundstrup, Emil; Pedersen, Mogens T.; Sjøgaard, Gisela; Andersen, Lars L. (2014†). "Time-wise change in neck pain in response to rehabilitation with specific resistance training: implications for exercise prescription." PloS one **9**(4): e93867.
Zebis, M. K. A., Lars L.; Pedersen, Mogens T.; Mortensen, Peter; Andersen, Christoffer H.; Pedersen, Mette M.; Boysen, Marianne; Roessler, Kirsten K.; Hannerz, Harald; Mortensen, Ole S.; Sjøgaard, Gisela (2011†). "Implementation of neck/shoulder exercises for pain relief among industrial workers: a randomized controlled trial." BMC Musculoskeletal Disorders **12**(1): 205-205.
Καρμίρης, Α. Γ., Ασημένια (2012). "Comparison of the effectiveness of two exercise programs for the rehabilitation of problems due to cervical syndrome." Physiotherapy Issues / Themata Fisikotherapeias **8**(3): 53-66.
