# Supplementary material for: Effectiveness and optimal dosage of exercise training for chronic non-specific neck pain: A systematic review with a narrative synthesis
Source: PLoS One. 2020 Jun 10;15(6):e0234511. doi: 10.1371/journal.pone.0234511 (PMC7286530; doi:10.1371/journal.pone.0234511)
Supplement: S7 Appendix — (PDF) [file pone.0234511.s007.pdf]

S7 Appendix Table of Characteristics and Intervention Data

**Author., Date**<br><br><u>Country</u><br>*Setting*	**Study Characteristics**	**Participant Information**	**Intervention Information**<br><br>Name - (n) - ***<u>Treatment Category</u>***<br>*Intervention Description*	**Outcome Measures and Reported Results**
Chung et al., 2018 [13]<br><br><u>South Korea</u><br><br>*University Rehabilitation Centre*<br><br>Authors contacted. No Response<br><br>Funding: Not reported	Single Blind Parallel RCT<br><br><u>Power Calculations:</u> *Yes – 58 in each group required to account for dropouts*<br><br><u>Adherence:</u> *Not reported*<br><br><u>Treatment Fidelity:</u><br>*Participants had supervised sessions. Not reported whether the individuals delivering the interventions were audited*	*<u>Description:</u>*<br>*General Population*<br><br><u>Age (mean ± SD):</u><br>*Gp1 34.27 ± 8.74; Gp2. 37.37 ± 10.24*<br><br><u>Sample Size (% F):</u><br>*50 (not reported)*<br><br><u>Inclusion Criteria:</u><br>• > 3 months neck pain<br>• Palpable cervical joint tenderness<br>24 mmHg or less on CCF Test	***1.*** Craniocervical Exercise Group - (25) - ***<u>Motor Control Exercises</u>***<br><br>***<u>Materials:</u>*** *Biofeedback Pressure Unit, Stabilizer Pressure Biofeedback, Chattanooga, Hixson TN, USA* ***<u>Who:</u>*** *2 physiotherapists with more than 5 years MSK experience. All physiotherapists were trained in the intervention protocol* ***<u>How:</u>*** *One to one supervised session's* ***<u>Where:</u>*** University Rehabilitation Centre<br>***<u>What:</u>*** *(1) Warm up – neck stretches (2) Craniocervical flexion in supine (3) Cool down – neck stretches*<br>***<u>When and How Much:</u>*** *Duration – 8 weeks; Frequency – three per week; Intensity – (2) 10x10 second holds, 3-5 seconds rest* ***<u>Tailoring & Modifications:</u>*** *Exercise dosage individualised based on CCF Test performance; Modifications – craniocervical flexion level increased by 2mmHg once 10x10 achieved without abnormal movement patterns*<br><br>2. Neck Isometric Exercise Group - (25) - ***<u>Pillar Exercises</u>***<br><br>***<u>Materials:</u>*** *None reported* ***<u>Who:</u>*** *2 physiotherapists with more than 5 years MSK experience. All physiotherapists were trained in the intervention protocol* ***<u>How:</u>*** *One to one supervised session's* ***<u>Where:</u>*** University Rehabilitation Centre<br>***<u>What:</u>*** *Week 1 - (1) Warm up – neck stretches (2) Cervical isometrics in supine* (3) Cool down – neck stretches; Weeks 2-8 – (1) (4) Cervical isometric flexion/extension/rotation/lateral flexion in sitting using hand as resistance (3)*<br>***<u>When and How Much:</u>*** *Duration – 8 weeks; Frequency – three per week; Intensity – (2)(4) 10-15x10 second holds, 15 seconds rest*<br>***<u>Tailoring & Modifications:</u>*** *Unclear whether exercise dosage individualised; Modifications – not reported*	<u>Pain:</u><br>*VAS*<br><br><u>Disability:</u><br>*NDI*<br><br>*Cervical lordosis Primary Outcome Measure*<br><br><u>Follow Up Periods</u><br>*4 weeks, 8 weeks*<br><br>*Primary End Point not reported*<br><br><u>Summary Results</u><br><u>Pain:</u><br>*Significant difference in means in favour of Gp1 (p= 0.031)*<br><br><u>Disability:</u><br>*Significant difference in means in favour of Gp1 (p= 0.016)*
Jordan et al., 1998 [14]<br><br><u>Denmark</u><br><br>*Hospital*<br><br>Unable to contact authors due to incorrect contact details.<br><br>Funding: Received‖	Single Blind Parallel RCT<br><br><u>Power Calculations:</u> *Not reported*<br><br><u>Adherence:</u> *Not reported*<br><br><u>Treatment Fidelity:</u><br>*Participants had supervised sessions. Not reported whether the individuals delivering the interventions were audited*	*<u>Description:</u>*<br>*Office Based Workers*<br><br><u>Age (mean ± SD):</u><br>*36.8 (range - 20 – 58)*<br><br><u>Sample Size (% F):</u><br>*119 (74%)*<br><br><u>Inclusion Criteria:</u><br>• Aged 20 – 60<br>• > 3 months neck pain<br>• lives close to hospital	***1.*** Intensive Training Group - (40) - ***<u>Segmental + Upper Limb Resistance Training Exercises + Another Intervention</u>***<br><br>***<u>Materials:</u>*** *Stationary bike*; Neck exercise unit, Follo, Norway; Hand-held weights*; Lat pull down machine** ***<u>Who:</u>*** *Physiotherapist* ***<u>How:</u>*** *Supervised group Class + HEP* ***<u>Where:</u>*** *Not reported + Home*<br>***<u>What:</u>*** *(1) Single 1.5-hour neck school (2) Cervical flexion using neck exercise unit (3) Cervical extension/lateral flexion (4) Shoulder, scapular, chest exercises using hand-held weights*, lat pull down (5) Cool down – Static Bike; HEP (6) 5 strengthening exercises for neck and shoulders* (7) 3 stretching exercises*<br>***<u>When and How Much:</u>*** *Duration – 6 weeks; Frequency – Supervised sessions - twice per week, HEP – not reported; Intensity – Supervised sessions – (2) 1x12 @ 30% MVC (3) 3x12 @ 30% MVC (4)(6) not reported* ***<u>Tailoring & Modifications:</u>*** *Exercise dosage individualised for cervical exercise only; Modifications – not reported*<br><br>***2.*** Physiotherapy Treatment Group - (39) -***<u>Passive Physiotherapy + Another Intervention</u>***<br><br>***<u>Materials:</u>*** *None reported* ***<u>Who:</u>*** *Physiotherapist* ***<u>How:</u>*** *One to one supervised session's + HEP* ***<u>Where:</u>*** *Not reported + Home*<br>***<u>What:</u>*** *(1) Single 1.5-hour neck school (2) Passive physiotherapy; HEP (3) 5 strengthening exercises for neck and shoulders* (4) 3 stretching exercises*<br>***<u>When and How Much:</u>*** *Duration – 6 weeks; Frequency – Supervised sessions - twice per week, HEP – not reported; Intensity – (3)(4) not reported* ***<u>Tailoring & Modifications:</u>*** *Unclear whether exercise dosage individualised; Modifications – not reported*<br><br>3. Manipulative Treatment Group - (40) - ***<u>Manipulation + Another Intervention</u>***<br><br>***<u>Materials:</u>*** *None reported* ***<u>Who:</u>*** *Chiropractor* ***<u>How:</u>*** *One to one supervised session's + HEP* ***<u>Where:</u>*** *Not reported + Home*<br>***<u>What:</u>*** *(1) Single 1.5-hour neck school (2) Manipulation; HEP (3) 5 strengthening exercises for neck and shoulders* (4) 3 stretching exercises*<br>***<u>When and How Much:</u>*** *Duration – 6 weeks; Frequency – Supervised sessions - twice per week, HEP – not reported; Intensity – (3)(4) not reported* ***<u>Tailoring & Modifications:</u>*** *Unclear whether exercise dosage individualised. Manipulation targeted at palpable dysfunctions; Modifications – 2 participants excluded as no palpable dysfunction on examination*	<u>Pain:</u><br>*11 Point Box Scale*<br><br><u>Disability:</u><br>*Self-Reporting Disability*<br><br>*Patient Perceived Effect and Physicians Global Assessment Primary Outcome Measures*<br><br><u>Follow Up Periods</u><br>*6 weeks, 6 months, 14 months*<br><br>*Primary End Point not reported*<br><br><u>Summary Results#</u><br><u>Pain:</u><br>*No difference in median values between groups (p > 0.05) at any time point*<br><br><u>Disability:</u><br>*No difference in median values between groups (p > 0.05) at any time point*

**Author., Date**<br><br>Country<br>*Setting*	**Study Characteristics**	**Participant Information**	**Intervention Information**<br><br>Name - (n) - ***Treatment Category***<br>*Intervention Description*	**Outcome Measures and Reported Results**
Waling et al., 2002 [49]<br><br>Sweden<br><br>*Not reported*<br><br>Authors replied to queries and no longer possess study data<br><br>Funding: Received‖	Parallel RCT<br><br>Power Calculations: *Reported as completed but calculations and target sample size not provided*<br><br>Adherence: *Measured by session attendance – Gp1. 85% attendance; Gp2. 82% attendance; Gp3. 80% attendance; Gp4. 77%*<br><br>Treatment Fidelity: *Participants had supervised sessions. Not reported whether the individuals delivering the interventions were audited*	*Description:*<br>*Office Based Workers*<br><br>Age (mean ± SD):<br>*38.2/37.9* ± 5.8<br><br>Sample Size (% F):<br>*126 (100%)*<br><br>Inclusion Criteria:<br>• > 45 years old<br>• Fully employed<br>• Pain in neck or upper trapezius region, decreased ROM, 1 or more trigger points on palpation of trapezius<br>• > 1 year neck pain<br>• Pain requires extra effort or break at work	***1.*** Strength Group - (34) - ***Upper Limb Resistance Training Exercises***<br><br>***Materials:*** *Airmachine** ***Who:*** *Physiotherapist* ***How:*** *Supervised group class* ***Where:*** *Fitness Centre*<br>***What:*** *(1) Warm up – no details (2) Concentric phase only of Row, triceps press, shoulder press, lat pull down*<br>***When and How Much:*** *Duration – 10 weeks; Frequency – three per week; Intensity – Weeks 1-4 (2) 2x12RM; Weeks 5-10 (2) 3x12RM*<br>***Tailoring & Modifications:*** *Exercise dosage individualised using RM; Modifications – Load increased once 3 sets performed comfortably*<br><br>***2.*** Endurance Group - (34) - ***Aerobic Exercise + Upper Limb Resistance Training Exercise***<br><br>***Materials:*** *Arm ergometer*; Rubber expanders** ***Who:*** *Physiotherapist* ***How:*** *Supervised group class* ***Where:*** *Unclear*<br>***What:*** *(1) Warm up – no details (2) Arm ergometer (3) Upper limb exercises using rubber expanders*<br>***When and How Much:*** *Duration – 10 weeks; Frequency – three per week; Intensity – (2) 110-120 BPM (3) 3-4x30-35RM* ***Tailoring & Modifications:*** *Exercise dosage individualised using RM and Heart Rate; Modifications – not reported*<br><br>***3.*** Co-ordination Group - (31) - ***Body Awareness Training***<br><br>***Materials:*** *None reported* ***Who:*** *Physiotherapist* ***How:*** *Supervised group class* ***Where:*** *Unclear*<br>***What:*** *(1) Warm up – no details (2) Body awareness training*<br>***When and How Much:*** *Duration – 10 weeks; Frequency – three per week; Intensity – NA* ***Tailoring & Modifications:*** *NA*<br><br>4. Control Group - (27) - ***Education and Stress Reduction***<br><br>***Materials:*** *Course led booklet on stress management* ***Who:*** *Occupational Nurse* ***How:*** *Supervised group class* ***Where:*** *Unclear*<br>***What:*** *(1) Stress management education*<br>***When and How Much:*** *Duration – 10 weeks; Frequency – once per week; Intensity – NA* ***Tailoring & Modifications:*** *NA*	Pain:<br>*VAS at Present*<br>*VAS in General*<br>*VAS at Worst*<br>*(Primary Outcome Measures)*<br><br>Disability:<br>*None Reported*<br><br>Follow Up Periods<br>*10-12 weeks, 8 months, 17 months, 3 years*<br><br>*Primary End Point not reported*<br><br>Summary Results<br>Pain:<br>*Significant difference in means between Gp1 and Gp4 @ 10-12 weeks (p < 0.0.5). No other difference between groups at any time point (p > 0.05)*
Bobos et al., 2016 [51]<br><br>Greece<br><br>*Not reported*<br><br>Authors replied to queries and provided raw data<br><br>Funding: None received	Single Blind Parallel RCT<br><br>Power Calculations: *Yes – 21 in each group required*<br><br>Adherence: *Not reported*<br><br>Treatment Fidelity: *Participants had supervised sessions. Not reported whether the individuals delivering the interventions were audited*	Description:<br>*General Population*<br><br>Age (mean ± SD):<br>*39.45 ± 12.67*<br><br>Sample Size (% F):<br>*67 (72%)*<br><br>Inclusion Criteria:<br>• Aged 18 – 65<br>• > 3 months neck pain<br>• NDI ≥ 5/50<br>• At least 1 active or latent MTrP in neck muscles	***1.*** Deep Neck Flexor Group - (23) - ***Motor Control + Segmental Exercises + Another Intervention***<br><br>***Materials:*** *Home exercise programme leaflet; DVD with standard exercises and ergonomic advice; air pressure biofeedback unit*; towel/elastic belt** ***Who:*** *Physiotherapist – trained in intervention protocol* ***How:*** *One to one supervised session + HEP* ***Where:*** *Not reported*<br>***What:*** (1) *Craniocervical Flexion in Supine with feedback unit; (2) Cervical flexion/extension in supine/prone (Craniocervical neutral not maintained throughout movement); (3) Nodding from prone position; (4) Nodding in standing with head against wall; (5) Standard exercise leaflet with stretches and isometric exercises*<br>***When and How Much:*** *Duration – 7 weeks; Frequency Supervised sessions – twice per week, HEP – not reported; Intensity – not reported*<br>***Tailoring & Modifications:*** *Unclear – not reported*<br><br>***2.*** Superficial Muscle Group - (22) - ***General Neck Exercise + Another Intervention***<br><br>***Materials:*** *Home exercise programme leaflet; DVD with standard exercises and ergonomic advice; air pressure biofeedback unit*; towel/elastic belt*; elastic band** ***Who:*** *Physiotherapist – trained in intervention protocol* ***How:*** *One to one supervised session + HEP* ***Where:*** *Not reported*<br>***What:*** (1) *Posterior head movement from sitting position; (2) Posterior head movement from supine position; (3) Movement in all directions in prone; (4) Cat-camel; (5) Standard exercise leaflet with stretches and isometric exercises*<br>***When and How Much:*** *Duration – 7 weeks; Frequency Supervised sessions – twice per week, HEP – not reported; Intensity – not reported*<br>***Tailoring & Modifications:*** *Unclear – not reported*<br><br>3. Control Group - (22) – ***Another Intervention***<br><br>***Materials:*** *Home exercise programme leaflet; DVD with standard exercises and ergonomic advice; towel/elastic belt** ***Who:*** *NA* ***How:*** *HEP Only* ***Where:*** *Not reported*<br>***What:*** (1) *Standard exercise leaflet with stretches and isometric exercises*<br>***When and How Much:*** *Duration – 7 weeks; Frequency HEP – not reported; Intensity – not reported* ***Tailoring & Modifications:*** *Unclear – not reported*	Pain:<br>*NRS Now*<br>*NRS at Best*<br>*NRS at Worst*<br><br>Disability:<br>*NDI†*<br><br>Follow Up Period<br>*7 weeks‡*<br><br>Summary Results<br>Pain:<br>*Significant difference in means between Gp1 and Gp2/3 (p < 0.05)*<br><br>Disability:<br>*Significant difference in means between Gp1 and Gp2/3 (p < 0.05)*

**Author., Date**<br><br>Country<br>*Setting*	**Study Characteristics**	**Participant Information**	**Intervention Information**<br><br>Name - (n) - ***Treatment Category***<br>*Intervention Description*	**Outcome Measures and Reported Results**
Ylinen et al., 2007 [54]<br><br>Finland<br><br>*Rehabilitation Centre*<br><br>Authors replied to queries<br><br>Funding: Received‖	Single Blind Parallel RCT<br><br>Power Calculations: *Yes – 60 in each group required to account for dropouts*<br><br>Adherence: *Measured with an exercise diary. Mean completion of HEP per week @ 12 months - Gp1. 1.7; Gp2. 2.0; Gp3. 2.0. % completing no exercise @ 3 years – Gp1. 40%; Gp2. 35%; Gp3. NA. % completing regular neck exercise @ 3 years. Gp1. 17%; Gp2. 25%; Gp3. NA. % completing regular shoulder exercise @ 3 years. Gp 1. 27%; Gp2. 32%; Gp3. NA*<br><br>Treatment Fidelity: *Participants had supervised sessions initially to learn exercise programme. Follow ups at 2 months and 6 months were used to check exercise programme. Not reported whether the individuals delivering the interventions were audited*	*Description:*<br>*Office Based Workers*<br><br>Age (mean ± SD):<br>*46 ± 6*<br><br>Sample Size (% F):<br>*180 (100%)*<br><br>Inclusion Criteria:<br>• Female<br>• Aged 25 – 53<br>• ≥ 6 months neck pain<br>• Permanently employed<br>• Motivated to continue work<br>• Motivated to rehabilitation	***1.*** Strength Group - (60) – ***Pillar + Upper Limb Resistance Training Exercises + Another Intervention***<br><br>***Materials:*** *Theraband with head attachment, Hygiene Corp, Akron, Ohio, USA; Dumbbells*; Written instructions on exercises* ***Who:*** *Experienced Physiotherapist* ***How:*** *Supervised group class + HEP* ***Where:*** *Rehabilitation Centre + Home*<br>***What:*** *(1) Cervical isometric flexion/extension/oblique flexion using theraband (maintain cervical neutral while moving trunk against resistance of theraband) (2) Dumbbell shrugs, shoulder press, curls, bent over rows, flys, pull overs (3) Lower body exercise (4) Stretching (5) Aerobic exercise (6) Manual therapy (7) 12-day neck school*<br>***When and How Much:*** *Duration – Supervised sessions – 2 weeks, HEP – 12 months; Frequency – Supervised sessions – six per week, HEP – three per week; Intensity – (1) 1x15 @ 80% maximal isometric strength (2) 1x15RM* ***Tailoring & Modifications:*** *Exercise dosage individualised using RM and % maximal isometric strength; Modifications – load modified based on measurement testing at 2 months, 6 months and 1 year. Participants advised to increase load if they could achieve 15 reps easily for upper body exercises*<br><br>***2.*** Endurance Group - (60) - ***Segmental + Upper Limb Resistance Training Exercises + Another Intervention***<br><br>***Materials:*** *2kg Dumbbells*; Written instructions on exercises* ***Who:*** *Experienced Physiotherapist* ***How:*** *Supervised group class + HEP* ***Where:*** *Rehabilitation Centre + Home*<br>***What:*** *(1) Cervical flexion in supine (Craniocervical neutral not maintained throughout movement) (2) Dumbbell shrugs, shoulder press, curls, bent over rows, flys, pull overs (3) Lower body exercise (4) Stretching (5) Aerobic exercise (6) Manual therapy (7) 12-day neck school*<br>***When and How Much:*** *Duration – Supervised sessions – 2 weeks, HEP – 12 months; Frequency – Supervised sessions – six per week, HEP – three per week; Intensity – (1) 3x20 @ weight of head (2) 3x20 @ 2kg* ***Tailoring & Modifications:*** *Exercise dosage not individualised as fixed at 2kg for all participants; Modifications – not reported*<br><br>3. Control Group - (60) – ***Stretching***§<br><br>***Materials:*** *Written instructions on exercises* ***Who:*** *Experienced Physiotherapist* ***How:*** *Supervised group class + HEP* ***Where:*** *Rehabilitation Centre + Home*<br>***What:*** *(1) Stretching*<br>1. ***When and How Much:*** *Duration – Supervised sessions – 3 days, HEP – 12 months; Frequency – HEP – three per week; Intensity – NA* ***Tailoring & Modifications:*** *NA*	Pain:<br>*VAS†*<br><br>Disability:<br>*Neck and Shoulder Pain and Disability Index†*<br>*NDI†*<br><br>Follow Up Periods<br>*2 months, 6 months, 12 months, 3 years*<br><br>*Primary End Point not reported*<br><br>Summary Results¶<br>Pain:<br>*No difference between Gp1 and Gp2 (p > 0.05) at any time point*<br><br>Disability:<br>*No difference in median values between Gp1 and Gp2 (p > 0.05) at any time point*
Chiu et al., 2005 [57]<br><br>Hong Kong<br><br>*Hospital*<br><br>Authors contacted. No Response. Data taken from (1)<br><br>Funding: Received‖	Single Blind Parallel RCT<br><br>Power Calculations: *Yes – 60 in each group required*<br><br>Adherence: *Not reported*<br><br>Treatment Fidelity: *Participants had supervised sessions. Not reported whether the individuals delivering the interventions were audited*	*Description:*<br>*General Population*<br><br>Age (mean ± SD):<br>*Gp1. 44.31 ± 9.77; Gp2. 42.70 ± 9.77; Gp3. 43.28 ± 9.69*<br><br>Sample Size (% F):<br>*218 (68%)*<br><br>Inclusion Criteria:<br>• Aged 20 – 70<br>• > 3 months neck pain	1. Exercise Group - (67) - ***Motor Control + Segmental Exercises + Another Intervention***<br><br>***Materials:*** *Biofeedback Unit, Stabilizer, Chattanooga, South Pacific, Australia; Multi Cervical Rehabilitation Unit, Hanoun Medical Inc, Ontario; Infrared Lamp, Hanovia Model 10, UK* ***Who:*** *Experienced Physiotherapist* ***How:*** *One to one supervised session's* ***Where:*** *Hospital*<br>***What:*** *(1) 20 mins infrared and neck care advice; (2) Craniocervical flexion in supine (3) Cervical flexion and extension warm up (4) Cervical flexion and extension using multi cervical rehabilitation unit*<br>***When and How Much:*** *Duration – 6 weeks; Frequency – twice per week; Intensity – (2) 22 – 30 mmHg, 10 second holds, 15 seconds rest, 10 mins (3) 1x15 @ 20% peak isometric strength (4) 3x8 – 12 @ 30% peak isometric strength,5 mins rest between sets* ***Tailoring & Modifications:*** *Exercise dosage individualised using % of peak isometric strength and highest possible level without abnormal movement patterns when performing craniocervical flexion; Modifications – cervical flexion and extension load increased by 5% peak isometric strength once able to complete 12 reps*<br><br>***2.*** TENs Group - (73) - ***TENs + Another Intervention***<br><br>***Materials:*** *TENs Unit, ITO, Model 130z; Infrared Lamp, Hanovia Model 10, UK* ***Who:*** *Experienced Physiotherapist* ***How:*** *One to one supervised session's* ***Where:*** *Hospital*<br>***What:*** *(1) 20 mins infrared and neck care advice; (2) 30 mins TENs*<br>***When and How Much:*** *Duration – 6 weeks; Frequency – twice per week* ***Tailoring & Modifications:*** *NA*<br><br>3. Control Group - (78) - ***Another Intervention***<br><br>***Materials:*** *Infrared Lamp, Hanovia Model 10, UK* ***Who:*** *Experienced Physiotherapist* ***How:*** *One to one supervised session's* ***Where:*** *Hospital*<br>***What:*** *(1) 20 mins infrared and neck care advice*<br>***When and How Much:*** *Duration – 6 weeks; Frequency – twice per week* ***Tailoring & Modifications:*** *NA*	Pain:<br>*NRS†*<br><br>Disability:<br>*Northwick Park Neck Pain Questionnaire†*<br><br>Follow Up Period<br>*6 weeks, 6 months*<br><br>*Primary End Point not reported*<br><br>Summary Results<br>Pain:<br>*No difference in mean change between groups at 6 weeks (p = 0.119) or 6 months (p = 0.122)*<br><br>Disability:<br>*Significant difference in mean change between Gp3 and Gp1/2 at 6 weeks (p= 0.016) and 6 months (p = 0.046)*

**Author., Date**<br><br>Country<br>*Setting*	**Study Characteristics**	**Participant Information**	**Intervention Information**<br><br>Name - (n) - ***Treatment Category***<br>*Intervention Description*	**Outcome Measures and Reported Results**
Javanshir et al., 2015 [59]<br><br>Iran<br><br>*University Physiotherapy Clinic*<br><br>Authors replied to queries<br><br>Funding: Not reported	Single Blind Parallel RCT<br><br>Power Calculations: *Yes – 30 in each group required*<br><br>Adherence: *Not reported*<br><br>Treatment Fidelity:<br>*Participants had supervised sessions. Not reported whether the individuals delivering the interventions were audited*	*Description:*<br>*General Population*<br><br>*Age (mean ± SD):*<br>*38 ± 4*<br><br>Sample Size (% F):<br>*60 (67%)*<br><br>Inclusion Criteria:<br>• > 3 months neck pain<br>Palpable cervical musculature tenderness	***1.*** Craniocervical Flexion Group - (30) - ***Motor Control Exercises***<br><br>***Materials:*** *Pressure Biofeedback Unit** ***Who:*** *Experienced physiotherapist* ***How:*** *One to one supervised session's + HEP* ***Where:*** *Physiotherapy Clinic + Home*<br>***What:*** *(1) Craniocervical flexion in supine*<br>***When and How Much:*** *Duration – 10 weeks; Frequency – Supervised sessions – three per week, HEP – Three per day; Intensity – (1) 10x10 second holds, 10 seconds rest* ***Tailoring & Modifications:*** *Exercise dosage individualised based on CCF Test performance; Modifications – craniocervical flexion level increased by 2mmHg once 10x10 achieved without abnormal movement patterns*<br><br>2. Cervical Flexion Group - (30) - ***Segmental Exercises***<br><br>***Materials:*** *Weighted sandbags** ***Who:*** *Experienced physiotherapist* ***How:*** *One to one supervised session's + HEP* ***Where:*** *Physiotherapy Clinic + Home*<br>***What:*** *(1) Cervical flexion in supine with craniocervical neutral maintained*<br>***When and How Much:*** *Duration – 10 weeks; Frequency – Supervised sessions – three per week, HEP – Three per day; Intensity – (1) Stage 1 (Weeks 0-2) 1x12-15RM, Stage 2 (Weeks 3-10) 3x15 @ original 12RM, 1 min rest between sets* ***Tailoring & Modifications:*** *Exercise dosage individualised using RM; Modifications – regressions and progressions used by performing exercise in sitting or adding 0.5kg weight respectively*	Pain:<br>*NRS*<br><br>Disability:<br>*NDI*<br><br>*Longus coli and sternocleidomastoid cross-sectional area Primary Outcome Measure*<br><br>Follow Up Periods<br>*10 weeks‡*<br><br>Summary Results<br>Pain:<br>*No difference in mean change between groups (p > 0.05)*<br><br>Disability:<br>*No difference in mean change between groups (p > 0.05)*
O'Leary et al., 2007 [60]<br><br>Australia<br><br>*Laboratory*<br><br>Authors replied to queries<br><br>Funding: Received‖	Single Blind Independent-group, repeated measures<br><br>Power Calculations: *Not reported*<br><br>Adherence: *NA- single session*<br><br>Treatment Fidelity:<br>*Participants had a single supervised session. Not reported whether the individuals delivering the interventions were audited*	*Description:*<br>*General Population*<br><br>*Age (mean ± SD):*<br>*Gp1. 41.1 ± 9.5; Gp2. 41.2 ± 11*<br><br>Sample Size (% F):<br>*48 (100%)*<br><br>Inclusion Criteria:<br>• > 3 months neck pain<br>• NDI > 5/50	***1.*** Craniocervical Flexion Co-ordination Group - (24) - ***Motor Control Exercises***<br><br>***Materials:*** *Biofeedback Pressure Unit, Stabilizer Pressure Biofeedback, Chattanooga, Hixson TN, USA* ***Who:*** *Not reported* ***How:*** *Single one to one supervised session (familiarisation session 48 hours prior)* ***Where:*** *Laboratory*<br>***What:*** *(1) Craniocervical flexion in supine*<br>***When and How Much:*** *Duration – single session; Frequency – NA; Intensity – (1) 10x10 second holds, 10 seconds rest* ***Tailoring & Modifications:*** *Exercise dosage individualised based on CCF Test performance; Modifications – NA*<br><br>2. Cervical Flexion Endurance Group - (24) - ***Segmental Exercises***<br><br>***Materials:*** *Biofeedback Pressure Unit, Stabilizer Pressure Biofeedback, Chattanooga, Hixson TN, USA* ***Who:*** *Not reported* ***How:*** *Single one to one supervised session (familiarisation session 48 hours prior)* ***Where:*** *Laboratory*<br>***What:*** *(1) Cervical flexion in supine with craniocervical neutral maintained*<br>***When and How Much:*** *Duration – single session; Frequency – NA; Intensity – (1) 3x10 @ 12RM, 3 second holds, 2 seconds rest between reps, 30 seconds rest between sets* ***Tailoring & Modifications:*** *Exercise dosage individualised based on RM; Modifications – NA*	Pain:<br>*VAS at Rest*<br>*VAS on Active Movements*<br><br>Disability:<br>*None Reported*<br><br>*Primary Outcome Measure not reported*<br><br>Follow Up Periods<br>*Immediately after 1st treatment session‡*<br><br>Summary Results<br>Pain:<br>*No difference in mean change between groups (p > 0.05)*<br><br>Disability:<br>*No difference in mean change between groups (p > 0.05)*

Author., Date<br><br>Country<br>*Setting*	Study Characteristics	Participant Information	Intervention Information<br><br>Name - (n) - ***Treatment Category***<br>*Intervention Description*	Outcome Measures and Reported Results
Rudolfsson et al., 2014 [61]<br><br>Sweden<br><br>*Laboratory*<br><br>Authors replied to queries. Further paper under process including all self-reported outcomes up to 15 months post baseline. Results not yet available.<br><br>Funding: Received‖	Single Blind Parallel RCT<br><br>Power Calculations: *Yes – 30 in each group required*<br><br>Adherence: *Measured by session attendance - Gp1. 5 dropouts; Gp2. 7 dropouts; Gp3. 2 dropouts*<br><br>Treatment Fidelity: *Participants had supervised sessions. Not reported whether the individuals delivering the interventions were audited*	*Description:*<br>*General Population*<br><br>Age (mean ± SD):<br>*Gp1. 50.7* ± 8.6; Gp2. *51.6* ± 9.0; Gp3. *51.2* ± 9.0<br><br>Sample Size (% F):<br>*128 (100%)*<br><br>Inclusion Criteria:<br>• Female<br>• Aged 25 – 65<br>• > 3 months neck pain<br>• > 9 normalized points of the first 19 items in the disability arm shoulder hand questionnaire	***1.*** Neck Co-ordination Exercise Group - (36) - ***Co-ordination Exercises***<br><br>***Materials:*** *Bespoke device for neck co-ordination training** ***Who:*** *Physiotherapists experienced in neck rehabilitation. All physiotherapists were trained in the intervention protocol for 8 hours* ***How:*** *One to one supervised session* ***Where:*** *Laboratory*<br>***What:*** *(1) Neck Co-ordination exercises*<br>***When and How Much:*** *Duration – 11 weeks; Frequency – twice per week; Intensity – NA* ***Tailoring & Modifications:*** *Unclear whether exercise dosage individualised; Modifications – not reported*<br><br>***2.*** Strength Training Group - (36) – ***Pillar + Upper Limb Resistance Training Exercises***<br><br>***Materials:*** *Cable pulley with head harness*; Dumbbell** ***Who:*** *Physiotherapists experienced in neck rehabilitation. All physiotherapists were trained in the intervention protocol for 8 hours* ***How:*** *One to one supervised session* ***Where:*** *Laboratory*<br>***What:*** *(1) Cervical isometric flexion/lateral flexion/rotation using pulley system (maintain cervical neutral while moving trunk against resistance of pulley system) (2) Shoulder press, chest press and seated row*<br>***When and How Much:*** *Duration – 11 weeks; Frequency – twice per week; Intensity – Sessions 1-3 (1) 1x15, 3 second holds, 11-13 BORG Scale (2) 2x15, 11-13 BORG Scale; Session 4 – 1RM testing; Session 5-11 (1) 1x15 @ 60% 1RM measured at session 4, RPE 13 (2) 2X12 @ 60% 1RM measured at session 4; Session 11 – 1RM testing; Session 12 onwards (1) 1x8 @ 75% 1RM measured at session 11 (2) 2x8 @ 75% 1RM measured at session 11* ***Tailoring & Modifications:*** *Exercise dosage individualised using % of RM measured at session 4 and 11, BORG Scale used sessions 1-3; Modifications – load increased throughout weeks relative to RM measurements*<br><br>***3.*** Massage Group - (36) – ***Massage***<br><br>***Materials:*** *None reported* ***Who:*** *Certified massage therapist with several years' experience. Massage therapists were trained in the intervention protocol for 8 hours* ***How:*** *One to one supervised session* ***Where:*** *Laboratory*<br>***What:*** *(1) Massage*<br>***When and How Much:*** *Duration – 11 weeks; Frequency – twice per week; Intensity – NA* ***Tailoring & Modifications:*** *NA*	Pain:<br>*NRS (Secondary Outcome Measure)*<br><br>Disability:<br>*Disabilities of the arm, shoulder and hand questionnaire; NDI (Secondary Outcome Measure)*<br><br>*Postural Sway and Goal Directed Arm Movement Tests Primary Outcome Measures*<br><br>Follow Up Periods<br>*3 months, 9 months‡; 15 months*<br><br>Summary Results<br>*Between group analysis not yet completed by study authors*
Yildiz et al., 2017 [62]<br><br>Turkey<br><br>*University*<br><br>Authors replied to queries<br><br>Funding: Not reported	Single Blind Parallel RCT<br><br>Power Calculations: *Reports sample adequately powered but calculations and target sample size not provided*<br><br>Adherence: *Measured with an exercise diary. Results not reported*<br><br>Treatment Fidelity: *Participants had supervised sessions. Not reported whether the individuals delivering the interventions were audited*	*Description:*<br>*General Population*<br><br>Age (mean ± SD):<br>*Gp1. 27.8* ± 8; Gp 2. *32.8* ± 7.4<br><br>Sample Size (% F):<br>*30 (NOT REPORTED)*<br><br>Inclusion Criteria:<br>• ≥ 6 months neck pain<br>• NDI 10 – 40/50<br>• Scapular dyskinesis rated by Kiblers rating system<br>•	***1.*** Intervention Group - (15) - ***Upper Limb Resistance Training + Segmental + Motor Control Exercises + Another Intervention***<br><br>***Materials:*** *Theraband, Hygiene Corp, Akron, Ohio, USA* ***Who:*** *Therapist* ***How:*** *One to one supervised session's + HEP* ***Where:*** *University + Home*<br>***What:*** *(1) Push up plus in an oblique position, scapular retraction with theraband, lateral pull down with theraband (2) Cervical flexion in supine with craniocervical neutral maintained (3) Cervical retraction against resistance (4) Craniocervical flexion in supine (5) Cervical stretches (6) Manual therapy*<br>***When and How Much:*** *Duration – 6 weeks; Frequency – Supervised sessions - once per week, HEP – twice per day; Intensity – (1) 3x10 (2) 2x10 (3) 2x10 @ 6/10 BORG Scale (4) 10x10 second holds* ***Tailoring & Modifications:*** *(2)(3)(4) exercise dosage individualised, unclear whether (1) exercise dosage individualised; Modifications – push up plus progress to prone position once able*<br><br>***2.*** Control Group - (15) - ***Segmental + Motor Control Exercises + Another Intervention***<br><br>***Materials:*** *Theraband, Hygiene Corp, Akron, Ohio, USA* ***Who:*** *Therapist* ***How:*** *One to one supervised session's + HEP* ***Where:*** *University + Home*<br>***What:*** *(1) Cervical flexion in supine with craniocervical neutral maintained (2) Cervical retraction against resistance (3) Craniocervical flexion in supine (4) Cervical stretches (5) Manual therapy*<br>***When and How Much:*** *Duration – 6 weeks; Frequency – Supervised sessions - once per week, HEP – twice per day; Intensity – (1) 2x10 (2) 2x10 @ 6/10 BORG Scale (3) 10x10 second holds* ***Tailoring & Modifications:*** *Exercise dosage individualised using BORG Scale and CCF performance; Modifications – not reported*	Pain:<br>*VAS†*<br><br>Disability:<br>*NDI*<br><br>Follow Up Periods<br>*1 week, 2 weeks, 3 weeks, 4 weeks, 5 weeks, 6 weeks‡*<br><br>Summary Results<br>Pain:<br>*No difference in means between groups (p = 0.917)*<br><br>Disability:<br>*No difference in means between groups (p= 0.1)*

**Author., Date**<br><br>Country<br>*Setting*	**Study Characteristics**	**Participant Information**	**Intervention Information**<br><br>Name - (n) - ***Treatment Category***<br>*Intervention Description*	**Outcome Measures and Reported Results**
Borisut et al., 2013 [63]<br><br>Thailand<br><br>*Office Workplaces*<br><br>Authors replied to queries<br><br>Funding: Not reported	Parallel RCT<br><br>Power Calculations: *Reported as completed but calculations and target sample size not provided*<br><br>Adherence: Measured by an *exercise diary – "over 80% in all groups"*<br><br>Treatment Fidelity: *Single session to teach exercises only*	Description:<br>*Female Office Based Workers*<br><br>Age (mean ± SD):<br>*Gp1. 32.72 ± 3.11; Gp2. 30.40 ± 3.54; Gp3. 30.16 ± 2.96; Gp4. 29.32 ± 3.11*<br><br>Sample Size (% F):<br>*100 (100%)*<br><br>Inclusion Criteria:<br><br>• Aged 20 – 35<br>• Work related neck pain<br>• > 6 months neck pain<br>• Works at a computer for 4 hours per day<br>• Pain > 3/10 VAS	***1.*** Strength – Endurance Group - (25) - ***Segmental Exercises***<br><br>***Materials:*** *None reported* ***Who:*** *Not reported* ***How:*** *HEP Only, single supervised session to teach exercise* ***Where:*** *Home*<br>***What:*** (1) *Cervical Flexion/Extension in Supine/Prone (Craniocervical neutral not maintained throughout movement)*<br>***When and How Much:*** *Duration – 12 weeks; Frequency – once per day; Intensity – Stage 1 (Weeks 1 – 4) 1x12 – 15RM, 1 min rest between sets; Stage 2 (Weeks 5 – 12) 3x15 @ original 12RM, 1 min rest between sets* ***Tailoring & Modifications:*** *Exercise dosage individualised using RM; Modifications - not reported*<br><br>***2.*** Craniocervical flexion Group - (25) - ***Motor Control Exercises***<br><br>***Materials:*** *Air filled pressure sensor** ***Who:*** *Not reported* ***How:*** *HEP Only, single supervised session to teach exercise* ***Where:*** *Home*<br>***What:*** (1) *Craniocervical flexion in supine*<br>***When and How Much:*** *Duration – 12 weeks; Frequency – once per day; Intensity – 22 – 30 mmHg, 15x10 second holds, 10 seconds rest*<br>***Tailoring & Modifications:*** *Unclear whether exercise dosage individualised using preselected level between 22 – 30 mmHg; Modifications - not reported*<br><br>***3.*** Combination Group - (25)- ***Motor Control + Segmental Exercises***<br><br>***Materials:*** *Air filled pressure sensor** ***Who:*** *Not reported* ***How:*** *HEP Only, single supervised session to teach exercise* ***Where:*** *Home*<br>***What:*** *(1) Cervical flexion/extension in supine/prone (Craniocervical neutral not maintained throughout movement); (2) Craniocervical flexion in supine*<br>***When and How Much:*** *Duration – 12 weeks; Frequency – once per day; Intensity – (1) - Stage 1 (Weeks 1 – 4) 1x12 – 15RM, 1 min rest between sets; Stage 2 (Weeks 5 – 12) 3x15 @ original 12RM, 1 min rest between sets; (2) 22 – 30 mmHg, 15x10 second holds, 10 seconds rest* ***Tailoring & Modifications:*** *Unclear whether exercise dosage individualised using preselected level between 22 – 30 mmHg; Modifications - not reported*<br><br>*4.* Control Group - (25) - ***No Treatment***	Pain:<br>*VAS*<br><br>Disability:<br>*NDI*<br><br>*Primary Outcome Measure not reported*<br><br>Follow Up Period<br>*12 weeks‡*<br><br>Summary Results<br>Pain:<br>*Significant difference in means between Gp4 and Gp1/2/3 (p < 0.001). Significant difference between Gp3 and Gp2/1 (p < 0.001). No difference between Gp2 and Gp1 (p > 0.05)*<br><br>Disability:<br>*Significant difference in means between Gp4 and Gp1/2/3 (p < 0.05). No other differences found (p > 0.05)*
Falla et al., 2013 [64]<br><br>Denmark<br><br>*Not reported*<br><br>Authors not contacted. No clarification required.<br><br>Funding: Received‖	Single Blind Parallel RCT<br><br>Power Calculations: *Yes – 23 in each group required*<br><br>Adherence: Not reported<br><br>Treatment Fidelity:<br>*Participants had supervised sessions. Not reported whether the individuals delivering the interventions were audited*	*Description:*<br>*General Population*<br><br>Age (mean ± SD):<br>*Gp1. 39.1 ± 8.7; Gp2. 38.6 ± 9.0*<br><br>Sample Size (% F):<br>*46 (100%)*<br><br>Inclusion Criteria:<br>• Aged 18 - 50<br>• Female<br>> 1-year neck pain	2. Exercise Group - (23) - ***Motor Control + Segmental Exercises***<br><br>***Materials:*** *Biofeedback Pressure Unit, Stabilizer Pressure Biofeedback, Chattanooga, Hixson TN, USA* ***Who:*** *Physiotherapist* ***How:*** *One to one supervised session's + HEP* ***Where:*** *Unclear + Home*<br>***What:*** *Stage 1 (1) Craniocervical flexion in supine; (2) Craniocervical extension/flexion/rotation in prone propped on elbows; Stage 2 (3) Cervical flexion with craniocervical flexion in supine; (4) Cervical extension with craniocervical neutral in 4-point kneeling*<br>***When and How Much:*** *Duration – 8 weeks; Frequency – Supervised sessions - once per week, HEP – Twice per day; Intensity – Stage 1 (1-2) – Unclear, Stage 2 (3-4) 1x15, 3 second holds* ***Tailoring & Modifications:*** *Unclear whether exercise dosage individualised using preselected level between 22 – 30 mmHg; Modifications – physiotherapists progressed exercise difficulty as able*<br><br>Control Group - (23) - ***No Treatment***	Pain:<br>*VAS*<br><br>Disability:<br>*NDI†*<br>*PSFS*<br><br>Follow Up Period<br>*8 weeks‡*<br><br>Summary Results<br>Pain:<br>*Significant difference in mean change in favour of Gp1 (p< 0.05)*<br><br>Disability:<br>*Significant difference in mean change in favour of Gp1 (p< 0.05)*

**Author., Date**<br><br>Country<br>*Setting*	**Study Characteristics**	**Participant Information**	**Intervention Information**<br><br>Name - (n) - ***Treatment Category***<br>*Intervention Description*	**Outcome Measures and Reported Results**
Li et al., 2017 [65]<br><br>China<br><br>*Industry*<br><br>Authors contacted. No Response<br><br>Funding: Received‖	Single Blind Parallel RCT<br><br>Power Calculations: *Yes – 20 in each group required*<br><br>Adherence: *Measured with an exercise diary. No statistically significant difference between progressive and fixed resistance training groups (p < 0.05)*<br><br>Treatment Fidelity: *Participants had supervised sessions. Not reported whether the individuals delivering the interventions were audited*	*Description:*<br>*Office Based Workers*<br><br>Age (mean ± SD):<br>*Gp1. 35.6* ± 7.9; Gp2. *33.7* ± 9.0; Gp3. *34.1* ± 8.2<br><br>Sample Size (% F):<br>*109 (100%)*<br><br>Inclusion Criteria:<br>• Female<br>• Aged 20-55<br>• Daily computer use with neck pain for more than 1 year<br>• Worked on a computer for at least 3 years<br>• Employed, motivated to continue working and rehabilitation<br>• Not having been on sick leave for more than 1 month during the last year<br>• Working at least 20 hours per week<br>• Experienced neck pain in the last 7 days at least 2/10 VAS	***1.*** Progressive Resistance Training Group - (38) - ***Pillar Exercises***<br><br>***Materials:*** *Standard instruction booklet about office ergonomics; Theraband, Hygiene Corp, Akron, Ohio, USA* ***Who:*** *Physiotherapist* ***How:*** *One to one supervised session's* ***Where:*** *Not reported*<br>***What:*** *(1) Standard instruction booklet about office ergonomics (2) Warm Up – General cervical and shoulder active range of movement exercises (3) Cervical isometric flexion/extension/lateral flexion using theraband attached to fixed stable object (Trunk movement was not performed to create resistance against the band)*<br>***When and How Much:*** *Duration – 6 weeks; Frequency – three per week; Intensity – (3) Weeks 1-2 – 8-12 @ 30% maximal strength, 5 seconds rest; Weeks 3-4 – 8-12 @ 50% maximal strength measured at week 2, 5 seconds rest; Weeks 5-6 – 8-12 @ 70% maximal strength measured at week 4, 5 seconds rest* ***Tailoring & Modifications:*** *Exercise dosage individualised using % of maximal isometric strength measured at baseline, week 2 and week 4. Perceived exertion scale also used; Modifications – load increased throughout weeks relative to strength measures*<br><br>*2.* Fixed Resistance Training Group - (35) - ***Pillar Exercises***<br><br>***Materials:*** *Standard instruction booklet about office ergonomics; Theraband, Hygiene Corp, Akron, Ohio, USA* ***Who:*** *Physiotherapist* ***How:*** *One to one supervised session's* ***Where:*** *Not reported*<br>***What:*** *(1) Standard instruction booklet about office ergonomics (2) Warm Up – General cervical and shoulder active range of movement exercises (3) Cervical isometric flexion/extension/lateral flexion using theraband attached to fixed stable object (Trunk movement was not performed to create resistance against the band)*<br>***When and How Much:*** *Duration – 6 weeks; Frequency – three per week; Intensity – (3) 8-12 @ 70% maximal strength, 5 seconds rest*<br>***Tailoring & Modifications:*** *Exercise dosage individualised using % of maximal isometric strength measured at baseline; Modifications – Load remained constant throughout all 6 weeks*<br><br>***3.*** Control Group - (36) – ***Education***<br><br>***Materials:*** *Standard instruction booklet about office ergonomics* ***Who:*** *Physiotherapist* ***How:*** *One to one supervised session's* ***Where:*** *Not reported*<br>***What:*** *(1) Standard instruction booklet about office ergonomics (2) Weekly discussions about workplace ergonomics, stress management, relaxation meditation and diet*<br>***When and How Much:*** *Duration – 6 weeks; Frequency – once per week; Intensity – NA* ***Tailoring & Modifications:*** *NA*	Pain:<br>*VAS†*<br><br>Disability:<br>*NDI*<br><br>Follow Up Periods<br>*2 weeks, 4 weeks, 6 weeks, 3 months*<br><br>*Primary End Point not reported*<br><br>Summary Results<br>Pain:<br>*Significant difference in means between Gp1 and Gp2/3, in favour of Gp1 (p< 0.05) @ 6 weeks and 3 months. Significant difference in means between Gp2 and Gp3 in favour of Gp2 (p < 0.05) @ 6 weeks and 3 months*<br><br>Disability:<br>*No difference in means between Gp1 and Gp2 at any time point 9p > 0.05). Significant difference in means between Gp1/2 and Gp3, in favour of Gp1/2 @ 6 weeks and 3 months (p< 0.05)*

**Author., Date**<br><br><u>Country</u><br>*Setting*	**Study Characteristics**	**Participant Information**	**Intervention Information**<br><br>Name - (n) - ***<u>Treatment Category</u>***<br>*Intervention Description*	**Outcome Measures and Reported Results**
Viljanen et al., 2003 [66]<br><br><u>Finland</u><br><br>*Occupational Health Centre*<br><br>Authors contacted. No Response<br><br>Funding: Received‖	Parallel RCT<br><br><u>Power Calculations:</u> *Yes – 80 in each group required*<br><br><u>Adherence:</u> *Measured by session attendance and exercise diary – Gp1. Average 13.6 sessions completed over 12 weeks (39%). Average of 31 mins per week completing HEP at 12 months. Gp2. Average 14.6 sessions completed over 12 weeks (42%). Average of 20 mins per week completing HEP at 12 months.*<br><br><u>Treatment Fidelity:</u> *Participants had supervised sessions and a 1-week re-enforcement training session at 6 months. Not reported whether the individuals delivering the interventions were audited*	*<u>Description:</u>*<br>*Office Based Workers*<br><br><u>Age (mean ± SD):</u><br>*Gp1. 45* ± 6.6; Gp2. *43* ± 7.3; Gp3. *44* ± 7.4<br><br><u>Sample Size (% F):</u><br>*393 (100%)*<br><br><u>Inclusion Criteria:</u><br>• > 3 months neck pain	1. Dynamic Muscle Training Group - (135) - ***<u>POOR DESCRIPTION + Upper Limb Resistance Training Exercises</u>***<br><br>***<u>Materials:</u>*** *Dumbbells** ***<u>Who:</u>*** *Physiotherapist* ***<u>How:</u>*** *Supervised group class + HEP* ***<u>Where:</u>*** *Occupational Health Centre + Home*<br>***<u>What:</u>*** *(1) Neck and shoulder exercises using dumbbells**<br>***<u>When and How Much:</u>*** *Duration – 12 weeks; Frequency – three per week; Intensity – (1) not reported* ***<u>Tailoring & Modifications:</u>*** *Unclear whether exercise dosage individualised; Modifications – not reported*<br><br>***2.*** Relaxation Training Group - (128) - ***<u>Relaxation Techniques</u>***<br><br>***<u>Materials:</u>*** *None reported* ***<u>Who:</u>*** *Physiotherapist* ***<u>How:</u>*** *Supervised group class + HEP* ***<u>Where:</u>*** *Occupational Health Centre + Home*<br>***<u>What:</u>*** *(1) Relaxation training using progressive relaxation methods, autogenic training, functional relaxation and systematic desensitisation*<br>***<u>When and How Much:</u>*** *Duration – 12 weeks; Frequency – three per week; Intensity – NA* ***<u>Tailoring & Modifications:</u>*** *NA*<br><br>Control Group - (130) - ***<u>No Treatment</u>***	<u>Pain:</u><br>*NRS†*<br><br><u>Disability:</u><br>*Authors own measure of disability*<br><br><u>Follow Up Periods</u><br>*3 months, 6 months, 12 months‡*<br><br><u>Summary Results</u><br><u>Pain:</u><br>*No difference in mean change between groups (p > 0.05) at any time point*<br><br><u>Disability:</u><br>*No difference in mean change between groups (p > 0.05) at any time point*
Suvrannato et al., 2019 [67]<br><br><u>Thailand</u><br><br>*Hospital*<br><br>Authors replied to queries and provided raw data<br><br>Funding: Not reported	Single Blind Parallel RCT<br><br><u>Power Calculations:</u><br>*Yes – 18 in each group required*<br><br><u>Adherence:</u> *Not reported*<br><br><u>Treatment Fidelity:</u> *Participants had supervised sessions. Not reported whether the individuals delivering the interventions were audited*	<u>Description:</u> *General Population*<br><br><u>Age (mean ± SD):</u><br>*Gp1. 43.27 ± 9.68*<br>*Gp2. 43.5 ± 12*<br>*Gp3. 42.05 ± 8.48*<br><br><u>Sample Size (%F)</u><br>*54 (89%)*<br><br><u>Inclusion Criteria:</u><br>• Aged 18-60<br>• Mechanical non-specific neck pain<br>• >3 months pain<br>• NDI-TH ≥10/100	1. Semispinalis cervicis-training group – (18) – ***<u>Pillar Exercises (Therapist Assisted)</u>***<br><br>***<u>Materials:</u>*** *None reported* ***<u>Who:</u>*** *Physiotherapist* ***<u>How:</u>*** *One to one supervised session's* ***<u>Where:</u>*** *Hospital*<br>***<u>What:</u>*** *(1) Isometric cervical extension isolating semispinalis cervicis with therapist assistance*<br>***<u>When and How Much:</u>*** *Duration – 6 weeks; Frequency – Supervised sessions – twice per week; Intensity – (1) 3x10, 10 second holds, 30 seconds rest between sets* ***<u>Tailoring & Modifications:</u>*** *Unclear – not reported*<br><br>2. Deep cervical flexor-training group – (18) – ***<u>Motor Control Exercises</u>***<br><br>***<u>Materials:</u>*** *Biofeedback Pressure Unit** ***<u>Who:</u>*** *Physiotherapist* ***<u>How:</u>*** *One to one supervised session's +HEP* ***<u>Where:</u>*** *Hospital + Home*<br>***<u>What:</u>*** *(1) Craniocervical flexion in supine*<br>***<u>When and How Much:</u>*** *Duration – 6 weeks; Frequency – Supervised sessions – twice per week, HEP – twice daily; Intensity – (1) 3x10, 10 second holds, 30 seconds rest between sets* ***<u>Tailoring & Modifications:</u>*** *Exercise dosage individualised based on CCF Test performance; Modifications – craniocervical flexion level increased by 2mmHg once 10x10 achieved without abnormal movement patterns*<br><br>***3.*** Usual Care – (18) – ***<u>Usual Care</u>***<br><br>***<u>Materials:</u>*** *None reported* ***<u>Who:</u>*** *Physiotherapist* ***<u>How:</u>*** *One to one supervised session's* ***<u>Where:</u>*** *Hospital*<br>***<u>What:</u>*** *Any treatment deemed appropriate by physiotherapist including stretching, upper limb strengthening, manual therapy, electrotherapy. Intervention excluded craniocervical flexion in supine and isometric cervical extension*<br>***<u>When and How Much:</u>*** *Duration – 6 weeks; Frequency – Supervised sessions – 10-12 over duration; Intensity – not reported* ***<u>Tailoring & Modifications:</u>*** *Unclear – not reported*	<u>Pain:</u><br>*NRS*<br><br><u>Disability:</u><br>*NDI-TH†*<br><br><u>Follow Up Periods</u><br>*6 weeks, 10 weeks, 18 weeks*<br><br>*Primary End Point not reported*<br><br><u>Summary Results</u><br><u>Pain:</u><br>*No difference between Gp1 and Gp2 (p>0.05) at any time point Significant difference between Gp2 and Gp3 @ 6 weeks (p = 0.029) only.*<br><br><u>Disability</u><br>*No difference between Gp1 and Gp2 (p >0.05) at any time point. Significant difference between Gp2 and Gp3 at all time points (p < 0.05)*

**Author., Date**<br><br>Country<br>*Setting*	**Study Characteristics**	**Participant Information**	**Intervention Information**<br><br>Name - (n) - ***Treatment Category***<br>*Intervention Description*	**Outcome Measures and Reported Results**
Shiravi et al., 2019 [68]<br><br>Iran<br><br>*Hospital*<br><br>Authors not contacted. No clarification required.<br><br>Funding: Not Received	Single Blind Parallel RCT<br><br>Power Calculations:<br>*Yes – 45 in each group required*<br><br>Adherence: *Not reported*<br><br>Treatment Fidelity: *Participants had supervised sessions. Not reported whether the individuals delivering the interventions were audited*	*Description:*<br>*Forward Head Population*<br><br>Age (mean ± SD):<br>*Gp1. 27.6* ± 2.06; Gp2. 25.11 ± 1.99; Gp3. *26* ± 1.65<br><br>Sample Size (% F):<br>*135 (100%)*<br><br>Inclusion Criteria:<br>• > 3 months neck pain<br>• VAS 3-8/10 at worst past 24 hours<br>• Forwarded head posture of >46°<br>• Rounded shoulder angle of >52°<br>•	1. Scapular Stabilization Exercise Group - (45) – ***Upper Limb Resistance Training Exercises***<br><br>***Materials:*** *None reported* ***Who:*** *Not reported* ***How:*** *One to one supervised session's* ***Where:*** *Hospital*<br>***What:*** *(1) Overhead press (2) Horizontal pull aparts (3) Chest Press (4) Serratus anterior punches (5) Retraction + external rotation (6) Scapular protraction (7) XY (8) TYW*<br>***When and How Much:*** *Duration – 6 weeks; Frequency – not reported; Intensity – not reported* ***Tailoring & Modifications:*** *Unclear – not reported*<br><br>2. Control Group - (45) – ***No Treatment***<br><br>3. Scapular Stabilization Exercises + Abdominal Control Feedback Group - (45) – ***Upper Limb Resistance Training Exercises + Another Intervention***§<br><br>***Materials:*** *None reported* ***Who:*** *Not reported* ***How:*** *One to one supervised session's* ***Where:*** *Hospital*<br>***What:*** *(1) Overhead press (2) Horizontal pull aparts (3) Chest Press (4) Serratus anterior punches (5) Retraction + external rotation (6) Scapular protraction (7) XY (8) TYW (9) Abdominal controlled feedback with inferior glides, isometric low row, dynamic knee push ups, wall press and wall slides*<br>***When and How Much:*** *Duration – 6 weeks; Frequency – not reported; Intensity – not reported* ***Tailoring & Modifications:*** *Unclear – not reported*	Pain:<br>*VAS*<br><br>Disability:<br>*None reported*<br><br>*Primary Outcome Measure not reported*<br><br>Follow Up Periods<br>*6-8 weeks*‡<br><br>Summary Results<br>Pain:<br>*Significant difference between Gp1 and Gp2 @ 6-8 weeks (p = 0.004)*
Gupta et al., 2010 [69]<br><br>India<br><br>*Rehabilitation Centre*<br><br>Unable to contact authors due to incorrect contact details.<br><br>Funding: Not reported	Parallel RCT<br><br>Power Calculations: *Not reported*<br><br>Adherence: *Measured with an exercise diary. Results not reported*<br><br>Treatment Fidelity: *Single session to teach exercises only*	*Description:*<br>*Early cervical spondylosis*<br><br>Age (mean ± SD):<br>*Gp1. 43.97 ± 9.93; Gp2. 50.83 ± 10.7*<br><br>Sample Size (% F):<br>*24 (Not reported)*<br><br>Inclusion Criteria:<br>• > 3 months neck pain<br>• Early spondylosis on X-ray<br>• NDI 5 – 24/50<br>• 24 mmHg or less on CCF Test<br>• Palpable cervical joint tenderness	***1.*** Experimental Group - (12) - ***Motor Control + Segmental + Pillar Exercises***<br><br>***Materials:*** *Air-filled pressure sensor, Stabilizer*; Photographic illustrations of exercises* ***Who:*** *Not reported* ***How:*** *HEP Only, single supervised session to teach exercise* ***Where:*** *Home*<br>***What:*** *Stage 1 (1) Craniocervical flexion in supine; Stage 2 (2) Cervical extension and return to neutral while maintaining craniocervical flexion; Stage 3 (3) Cervical extension and return to neutral with isometric holds throughout range, while maintaining craniocervical flexion*<br>***When and How Much:*** *Duration – 6 weeks; Frequency – Once per day; Intensity – (1) (3) 10x10 second holds at target level; (2) - Unclear*<br>***Tailoring & Modifications:*** *Exercise dosage individualised based on CCF Test performance; Modifications – exercises progressed once 30 mmHg achieved*<br><br>2. Conventional Group - (12) - ***Pillar Exercises***<br><br>***Materials:*** *Photographic illustrations of exercises* ***Who:*** *Not reported* ***How:*** *HEP Only, single supervised session to teach exercise* ***Where:*** *Home*<br>***What:*** *(1) Cervical flexion/extension/lateral flexion isometrics – not clear how isometrics were performed*<br>***When and How Much:*** *Duration – 6 weeks; Frequency – Once per day; Intensity – 30 mins* ***Tailoring & Modifications:*** *Unclear whether exercise dosage individualised; Modifications – not reported*	Pain:<br>*VAS at Rest*<br>*VAS on Activity*<br><br>Disability:<br>*NDI*<br><br>*Primary Outcome Measure not reported*<br><br>Follow Up Periods<br>*1 week, 2 weeks, 3 weeks, 4 weeks, 5 weeks, 6 weeks*<br><br>*Primary End Point not reported*<br><br>Summary Results<br>Pain:<br>*Significant difference in means in favour of Gp1 @ 6 weeks (p< 0.05)*<br><br>Disability:<br>*Significant difference in means in favour of Gp1 @ 6 weeks (p< 0.05)*

**Author., Date**<br><br>Country<br>*Setting*	**Study Characteristics**	**Participant Information**	**Intervention Information**<br><br>Name - (n) - ***Treatment Category***<br>*Intervention Description*	**Outcome Measures and Reported Results**
Gupta et al., 2013 [70]<br><br>India<br><br>*Dental College*<br><br>Authors contacted. No Response<br><br>Funding: None received	Parallel RCT<br><br>Power Calculations: *Not reported*<br><br>Adherence: *Not reported*<br><br>Treatment Fidelity: *Participants had supervised sessions. Not reported whether the individuals delivering the interventions were audited*	*Description:*<br>*Dental Surgeons*<br><br>Age (mean ± SD):<br>*26 ± 4.28*<br><br>Sample Size (% F):<br>*30 (40%)*<br><br>Inclusion Criteria:<br>• > 3 months neck pain<br>• NDI < 24/50<br>• Palpable cervical joint tenderness	1. Deep Cervical Flexor Training Group - (15) - ***Motor Control Exercises***<br><br>***Materials:*** *Biofeedback Pressure Unit, Stabilizer Pressure Biofeedback, Chattanooga, Hixson TN, USA* ***Who:*** *Not reported* ***How:*** *One to one supervised session's* ***Where:*** *Not reported*<br>***What:*** *(1) Craniocervical flexion in supine*<br>***When and How Much:*** *Duration – 4 weeks; Frequency - not reported; Intensity – not reported* ***Tailoring & Modifications:*** *Unclear whether exercise dosage individualised; Modifications – not reported*<br><br>2. Conventional Isometric Training Group - (15) – ***Pillar Exercises***<br><br>***Materials:*** *None reported* ***Who:*** *Not reported* ***How:*** *One to one supervised session's* ***Where:*** *Not reported*<br>***What:*** *(1) Cervical isometrics in sitting using hand for resistance**<br>***When and How Much:*** *Duration – 4 weeks; Frequency - not reported; Intensity – not reported* ***Tailoring & Modifications:*** *Unclear whether exercise dosage individualised; Modifications – not reported*	Pain:<br>*VAS*<br><br>Disability:<br>*NDI*<br><br>*Primary Outcome Measure not reported*<br><br>Follow Up Periods<br>*4 weeks‡*<br><br>Summary Results<br>Pain:<br>*Significant difference in means in favour of Gp1 (p= 0.001)*<br><br>Disability:<br>*Significant difference in means in favour of Gp1 (p= 0.000)*
Hingarajia et al., 2012 [71]<br><br>India<br><br>*Physiotherapy College*<br><br>Authors contacted. No Response<br><br>Funding: Not reported	Parallel RCT<br><br>Power Calculations: *Not reported*<br><br>Adherence: *Measured by exercise diary – 100% attendance and completion of HEP*<br><br>Treatment Fidelity: *Participants had supervised sessions. Not reported whether the individuals delivering the interventions were audited*	*Description:*<br>*Physiotherapy students*<br><br>Age (mean ± SD):<br>*Gp1. 20.12 ± 2.1; Gp2. 20.2 ± 1.76*<br><br>Sample Size (% F):<br>*50 (100%)*<br><br>Inclusion Criteria:<br>• Female physiotherapy student<br>• 3 months – 5 years neck pain<br>• VAS < 7/10<br>• NDI < 15/50	***1.*** Experimental Group - (25) - ***Motor Control + Pillar Exercises***<br><br>***Materials:*** *None reported* ***Who:*** *Physiotherapist* ***How:*** *One to one supervised session's + HEP* ***Where:*** *Physiotherapy college + Home*<br>***What:*** *(1) Craniocervical flexion in supine (2) Cervical isometrics flexion/extension/lateral flexion using hand as resistance*<br>***When and How Much:*** *Duration – 4 weeks; Frequency – Supervised sessions - twice per week, HEP – Twice per day; Intensity – (1) 3x10, 10 second holds, 1 min rest between sets (2) 1x15, 10 second holds* ***Tailoring & Modifications:*** *Exercise dosage individualised based on CCF Test performance. Not reported whether isometrics were individualised; Modifications – craniocervical flexion level increased by 2mmHg once 3x10 achieved without abnormal movement patterns*<br><br>2. Control Group - (25) - ***Pillar Exercises***<br><br>***Materials:*** *None reported* ***Who:*** *Physiotherapist* ***How:*** *One to one supervised session's + HEP* ***Where:*** *Physiotherapy college + Home*<br>***What:*** *(1) Cervical isometrics flexion/extension/lateral flexion using hand as resistance*<br>***When and How Much:*** *Duration – 4 weeks; Frequency – Supervised sessions - twice per week, HEP – Twice per day; Intensity – (1) 1x15, 10 second holds* ***Tailoring & Modifications:*** *Unclear whether exercise dosage individualised; Modifications – not reported*	Pain:<br>*VAS*<br><br>Disability:<br>*NDI*<br><br>*Primary Outcome Measure not reported*<br><br>Follow Up Periods<br>*4 weeks‡*<br><br>Summary Results<br>Pain:<br>*No difference in mean change between groups (p > 0.05)*<br><br>Disability:<br>*No difference in mean change between groups (p > 0.05)*

**Author., Date**<br><br>Country<br>*Setting*	**Study Characteristics**	**Participant Information**	**Intervention Information**<br><br>Name - (n) - ***Treatment Category***<br>*Intervention Description*	**Outcome Measures and Reported Results**
Izquierdo et al., 2016 [72]<br><br>Spain<br><br>*Not reported*<br><br>Authors contacted. No Response<br><br>Funding: Not reported	Single Blind Parallel RCT<br><br>Power Calculations: *Yes – 14 in each group required to account for dropouts*<br><br>Adherence: *Measured with an exercise diary. Results not reported*<br><br>Treatment Fidelity: *Participants had supervised sessions. Not reported whether the individuals delivering the interventions were audited*	*Description:*<br>*General Population*<br><br>Age (mean ± SD):<br>*Gp1. 28.43 ± 6.16; Gp2. 29.93 ± 7.34*<br><br>Sample Size (% F):<br>*28 (64%)*<br><br>Inclusion Criteria:<br>• Aged 18 – 55<br>• > 3 months neck pain<br>• NDI < 15/50<br>• 24 mmHg or less on CCF Test<br>Palpable cervical musculature tenderness	***1.*** Craniocervical Flexion Group - (14) - ***Motor Control Exercises***<br><br>***Materials:*** *Biofeedback Pressure Unit, Stabilizer Pressure Biofeedback, Chattanooga, Hixson TN, USA* ***Who:*** *Physiotherapist with 5 years' experience* ***How:*** *One to one supervised session's + HEP* ***Where:*** *University Laboratory + Home*<br>***What:*** *(1) Craniocervical flexion in supine*<br>***When and How Much:*** *Duration – 2 months; Frequency – Supervised sessions – once per week first 3 weeks then once every 2 weeks for 6 weeks, HEP – Twice per day; Intensity – (1) 10x10 second holds, 3-5 seconds rest* ***Tailoring & Modifications:*** *Exercise dosage individualised based on CCF Test performance; Modifications – craniocervical flexion level increased by 2mmHg once 10x10 achieved without abnormal movement patterns*<br><br>2. Proprioception Group - (14) - ***Proprioception Exercises***<br><br>***Materials:*** *Helmet with laser attachment*; Pupillary glasses** ***Who:*** *Physiotherapist with 5 years' experience* ***How:*** *One to one supervised session's + HEP* ***Where:*** *University Laboratory + Home*<br>***What:*** *(1) Cervical proprioception exercises including head relocation, eye-follow, gaze stability, eye-head-co-ordination*<br>***When and How Much:*** *Duration – 2 months; Frequency – Supervised sessions – once per week first 3 weeks then once every 2 weeks for 6 weeks, HEP – Twice per day; Intensity – (1) 30 mins* ***Tailoring & Modifications:*** *Unclear whether exercise dosage individualised; Modifications – not reported*	Pain:<br>*VAS – Minimum*<br>*VAS – Maximum*<br>*VAS – Rest*<br>*(Secondary Outcome Measures)*<br><br>Disability:<br>*NDI (Secondary Outcome Measure)*<br><br>*CCF Test Primary Outcome Measures*<br><br>Follow Up Periods<br>*Immediately after 1st treatment sessions, 1 month, 2 months*<br><br>*Primary End Point not reported*<br><br>Summary Results¶<br>Pain:<br>*Significant difference in mean change in favour of Gp1 @ 2 months only (p= 0.044). No other differences found*<br><br>Disability:<br>*No difference in mean change between groups (p > 0.05) at any time point*
Kaur et al., 2018 [73]<br><br>India<br><br>*Hospital and Research Centre and Physiotherapy Clinic*<br><br>Authors contacted. No Response<br><br>Funding: None received	Parallel RCT<br><br>Power Calculations: *Not reported*<br><br>Adherence: *NA - single session*<br><br>Treatment Fidelity:<br>*Participants had a single supervised session. Not reported whether the individuals delivering the interventions were audited*	*Description:*<br>*General population*<br><br>Age (mean ± SD):<br>*Not reported*<br><br>Sample Size (% F):<br>*34 (not reported)*<br><br>Inclusion Criteria:<br>• Aged 20 – 50<br>• > 3 months neck pain<br>• NRS > 3/10<br>• 24 mmHg or less on CCF Test	***1.*** Exercise Group - (17) -***Motor Control Exercises***<br><br>***Materials:*** *Sphygmomanometer** ***Who:*** *Not reported* ***How:*** *Single one to one supervised session* ***Where:*** *Physiotherapy Clinic*<br>***What:*** *(1) Craniocervical flexion in supine*<br>***When and How Much:*** *Duration – single session; Frequency – NA; Intensity – (1) 1 rep per 2 seconds for 1 min* ***Tailoring & Modifications:*** *Unclear whether exercise dosage individualised; Modifications – not reported*<br><br>2. Mobilisation Group - (17) -***Manual Therapy***<br><br>***Materials:*** *None reported* ***Who:*** *Not reported* ***How:*** *Single one to one supervised session* ***Where:*** *Physiotherapy Clinic*<br>***What:*** *(1) Passive grade III craniocervical flexion mobilisations*<br>***When and How Much:*** *Duration – single session; Frequency – NA; Intensity – (1) 1 rep per 2 seconds for 1 min* ***Tailoring & Modifications:*** *NA*	Pain:<br>*VAS*<br><br>Disability:<br>*None Reported*<br><br>*Primary Outcome Measure not reported*<br><br>Follow Up Periods<br>*Immediately after 1st treatment session‡*<br><br>Summary Results<br>Pain:<br>*Significant difference in means in favour of Gp1 (p = 0.0028)*

**Author., Date**<br><br>Country<br>*Setting*	**Study Characteristics**	**Participant Information**	**Intervention Information**<br><br>Name - (n) - ***Treatment Category***<br>*Intervention Description*	**Outcome Measures and Reported Results**
Kim et al., 2016 [74]<br><br>South Korea<br><br>*Rehabilitation Centre*<br><br>Authors contacted. No Response<br><br>Funding: Not reported	Single Blind Parallel RCT<br><br>Power Calculations: *Not reported*<br><br>Adherence: *Not reported*<br><br>Treatment Fidelity: *Single session to teach exercises only*	*Description:*<br>*General Population*<br><br>Age (mean ± SD):<br>*Gp1. 46.7* ± 4.2; Gp2. *45.4* ± 5.1<br><br>Sample Size (% F):<br>*30 (50%)*<br><br>Inclusion Criteria:<br>• > 3 months neck pain<br>• NDI < 15/50<br>• 22 mmHg or less on CCF Test<br>•	***1.*** Deep Cervical Flexor Group - (14) - ***Motor Control Exercises***<br><br>***Materials:*** *Biofeedback Pressure Unit, Stabilizer Pressure Biofeedback, Chattanooga, Hixson TN, USA* ***Who:*** *Not reported* ***How:*** *HEP Only, single supervised session to teach exercise* ***Where:*** *Home*<br>***What:*** *(1) Craniocervical flexion in supine*<br>***When and How Much:*** *Duration – 4 weeks; Frequency – three per week; Intensity – (1) 10x10-15 second holds, 3-5 seconds rest* ***Tailoring & Modifications:*** *Unclear whether exercise dosage individualised using preselected level between 22 – 30 mmHg; Modifications - not reported*<br><br>2. General Strengthening Exercises Group - (14) - ***Pillar Exercises + Another Intervention***<br><br>***Materials:*** *Elastic band*, Ball** ***Who:*** *Not reported* ***How:*** *HEP Only, single supervised session to teach exercise* ***Where:*** *Home*<br>***What:*** *(1) Cervical isometric flexion/extension/lateral flexion (unclear how isometrics performed) (2) Cervical isometric flexion/extension/lateral flexion pushing head against ball that's on the wall (3) Cervical stretching*<br>***When and How Much:*** *Duration – 4 weeks; Frequency – three per week; Intensity – (1)(2) 10x10 second holds (3) 3x3-5, 10 second holds*<br>***Tailoring & Modifications:*** *Unclear whether exercise dosage individualised; Modifications - not reported*	Pain:<br>*NRS*<br><br>Disability:<br>*NDI*<br><br>*Primary Outcome Measure not reported*<br><br>Follow Up Periods<br>*4 weeks, 12 weeks*<br><br>*Primary End Point not reported*<br><br>Summary Results<br>Pain:<br>*Significant difference in means in favour of Gp1 @ 12 weeks (p< 0.05)*<br><br>Disability:<br>*Significant difference in means in favour of Gp1 @ 12 weeks (p< 0.05)*
Kwan-Woo et al., 2016 [75]<br><br>South Korea<br><br>*Not reported*<br><br>Authors contacted. No Response<br><br>Funding: Not reported	Single Blind Parallel RCT<br><br>Power Calculations: *Not reported*<br><br>Adherence: *Not reported*<br><br>Treatment Fidelity: *Participants had supervised sessions. Not reported whether the individuals delivering the interventions were audited*	*Description:*<br>*General Population*<br><br>Age (mean ± SD):<br>*Not reported*<br><br>Sample Size (% F):<br>*51 (not reported)*<br><br>Inclusion Criteria:<br>• > 3 months neck pain<br>• Aged 18 – 60<br>• NDI > 20%<br>• Limited craniocervical and Thoracic flexion and extension ROM	***1.*** Thoracic Manipulation + Deep Craniocervical Flexor Group - (16) - ***Thoracic Manipulation + Motor Control Exercises***§<br><br>***Materials:*** *Biofeedback Pressure Unit, Stabilizer Pressure Biofeedback, Chattanooga, Hixson TN, USA* ***Who:*** *Not reported* ***How:*** *One to one supervised session's* ***Where:*** *Not reported*<br>***What:*** *(1) Thoracic manipulation (2) Craniocervical flexion in supine*<br>***When and How Much:*** *Duration – 10 weeks; Frequency – three per week; Intensity – (2) 10x10 second holds, 5 seconds rest* ***Tailoring & Modifications:*** *Exercise dosage individualised based on CCF Test performance; Modifications – craniocervical flexion level increased by 2mmHg once 10x10 achieved without abnormal movement patterns*<br><br>***2.*** Deep Craniocervical Flexor Group - (15) -***Motor Control Exercises***<br><br>***Materials:*** *Biofeedback Pressure Unit, Stabilizer Pressure Biofeedback, Chattanooga, Hixson TN, USA* ***Who:*** *Not reported* ***How:*** *One to one supervised session's* ***Where:*** *Not reported*<br>***What:*** *(1) Craniocervical flexion in supine (2) Cool down – cervical stretches*<br>***When and How Much:*** *Duration – 10 weeks; Frequency – three per week; Intensity – (1) 10x10 second holds, 5 seconds rest* ***Tailoring & Modifications:*** *Exercise dosage individualised based on CCF Test performance; Modifications – craniocervical flexion level increased by 2mmHg once 10x10 achieved without abnormal movement patterns*<br><br>3. Control Group - (15) - ***General Active Range of Movement Exercises***<br><br>***Materials:*** *None reported* ***Who:*** *Not reported* ***How:*** *One to one supervised session's* ***Where:*** *Not reported*<br>***What:*** *(1) General cervical active range of movement exercises*<br>3. ***When and How Much:*** *Duration – 10 weeks; Frequency – three per week; Intensity – (1) 35 mins* ***Tailoring & Modifications:*** *Unclear whether exercise dosage individualised; Modifications – not reported*	Pain:<br>*VAS*<br><br>Disability:<br>*NDI*<br><br>*Primary Outcome Measure not reported*<br><br>Follow Up Periods<br>*10 weeks‡*<br><br>Summary Results<br>Pain:<br>*Significant difference in mean change between Gp2 and Gp3, in favour of Gp2 (p< 0.05)*<br><br>Disability:<br>*Significant difference in mean change between Gp2 and Gp3, in favour of Gp2 (p< 0.05)*

**Author., Date**<br><br><u>Country</u><br>*Setting*	**Study Characteristics**	**Participant Information**	**Intervention Information**<br><br>Name - (n) - ***<u>Treatment Category</u>***<br>*Intervention Description*	**Outcome Measures and Reported Results**
Randlov et al., 1998 [76]<br><br><u>Denmark</u><br><br>*Hospital*<br><br>Authors contacted. No Response<br><br>Funding: Received[‖]	Parallel RCT<br><br><u>Power Calculations:</u> *Not reported*<br><br><u>Adherence:</u> *Not reported*<br><br><u>Treatment Fidelity:</u> *Participants had supervised sessions. Not reported whether the individuals delivering the interventions were audited*	<u>*Description:*</u><br>*General Population*<br><br><u>Age (mean ± SD):</u><br>*39 (range - 18 – 61)*<br><br><u>Sample Size (% F):</u><br>*77 (100%)*<br><br><u>Inclusion Criteria:</u><br>• Female<br>• Aged 18 – 65<br>• > 6 months neck/shoulder pain<br>• Lived short distance from hospital	***1.*** Light Training Group - (41) – ***<u>Segmental + Upper Limb Resistance Training Exercises</u>***<br><br>***<u>Materials:</u>*** *Not reported* ***<u>Who:</u>*** *Not reported* ***<u>How:</u>*** *Supervised group class* ***<u>Where:</u>*** *Hospital*<br>***<u>What:</u>*** *(1) Warm Up – hot pack, static bike and stretching (2) Cervical flexion/extension in supine/prone (Craniocervical neutral not maintained throughout movement) (3) Arm abduction, scapular retraction and shoulder extension, supine shoulder flexion, wall push ups*<br>***<u>When and How Much:</u>*** *Duration – 12 weeks; Frequency – three per week; Intensity – (2)(3) 1x20* ***<u>Tailoring & Modifications:</u>*** *Unclear whether exercise dosage individualised; Modifications – not reported*<br><br>2. Intensive Training Group - (36) – ***<u>Segmental + Upper Limb Resistance Training Exercises</u>***<br><br>***<u>Materials:</u>*** *Not reported* ***<u>Who:</u>*** *Not reported* ***<u>How:</u>*** *Supervised group class* ***<u>Where:</u>*** *Hospital*<br>***<u>What:</u>*** *(1) Warm Up – hot pack, static bike and stretching (2) Cervical flexion/extension in supine/prone (Craniocervical neutral not maintained throughout movement) (3) Arm abduction, scapular retraction and shoulder extension, supine shoulder flexion, wall push ups*<br>***<u>When and How Much:</u>*** *Duration – 12 weeks; Frequency – three per week; Intensity – (2)(3) 5x20* ***<u>Tailoring & Modifications:</u>*** *Unclear whether exercise dosage individualised; Modifications – weight increased for upper limb exercises throughout programme**	<u>Pain:</u><br>*11 Point Box Scale*<br><br><u>Disability:</u><br>*ADL Questionnaire*<br><br>*Primary Outcome Measure not reported*<br><br><u>Follow Up Periods</u><br>*12 weeks, 6 months, 12 months*<br><br>*Primary End Point not reported*<br><br><u>Summary Results[#]</u><br><u>Pain:</u><br>*No difference in median values between groups (p > 0.05) at any time point*<br><br><u>Disability:</u><br>*No difference in median values between groups (p > 0.05) at any time point*
Khan et al., 2014 [77]<br><br><u>Pakistan</u><br><br>*Private Practice & University Clinic*<br><br>Authors contacted. No Response<br><br>Funding: Not reported	Parallel RCT<br><br><u>Power Calculations:</u> *Reported as completed but calculations and target sample size not provided*<br><br><u>Adherence:</u> *Not reported*<br><br><u>Treatment Fidelity:</u><br>*Participants had supervised sessions. Not reported whether the individuals delivering the interventions were audited*	<u>*Description:*</u><br>*General Population*<br><br><u>Age (mean ± SD):</u><br>*34.43 ± 2.7*<br><br><u>Sample Size (% F):</u><br>*68 (60%)*<br><br><u>Inclusion Criteria:</u><br>• Aged 20 – 50<br>• 3 months - 3 years neck pain<br>MRI excluding major pathology	1. Isometric Neck Exercise Group - (34) – ***<u>Pillar Exercises + Another Intervention</u>***<br><br>***<u>Materials:</u>*** *Theraband** ***<u>Who:</u>*** *Physiotherapist with 2 years' experience* ***<u>How:</u>*** *One to one supervised session's + HEP* ***<u>Where:</u>*** *Not reported + Home*<br>***<u>What:</u>*** *(1) Cervical isometric flexion/extension/lateral flexion/rotation using theraband (Unclear how theraband was used); HEP (2) General cervical range of movement*<br>***<u>When and How Much:</u>*** *Duration – 12 weeks; Frequency – Supervised sessions – three per week, HEP – twice per day, 5 times per week; Intensity – (1)(2) 1x20* ***<u>Tailoring & Modifications:</u>*** *Unclear whether exercise dosage individualised; Modifications – not reported*<br><br>2. General Neck Exercise Group - (34) – ***<u>Another Intervention</u>***<br><br>***<u>Materials:</u>*** *Theraband** ***<u>Who:</u>*** *Physiotherapist with 2 years' experience* ***<u>How:</u>*** *One to one supervised session's + HEP* ***<u>Where:</u>*** *Not reported + Home*<br>***<u>What:</u>*** *(1) General cervical range of movement; HEP (1)*<br>***<u>When and How Much:</u>*** *Duration – 12 weeks; Frequency – Supervised sessions – three per week, HEP – twice per day, 5 times per week; Intensity – (1) 1x20* ***<u>Tailoring & Modifications:</u>*** *Unclear whether exercise dosage individualised; Modifications – not reported*	<u>Pain:</u><br>*VAS*<br><br><u>Disability:</u><br>*Northwick Park Neck Pain Questionnaire*<br><br>*Primary Outcome Measure not reported*<br><br><u>Follow Up Periods</u><br>*12 weeks‡*<br><br><u>Summary Results</u><br>*Between group analysis not completed by study authors*

Author., Date<br><br>Country<br>*Setting*	Study Characteristics	Participant Information	Intervention Information<br><br>Name - (n) - ***Treatment Category***<br>*Intervention Description*	Outcome Measures and Reported Results
Ulug et al., 2018 [78]<br><br>Turkey<br><br>*Not reported*<br><br>Authors contacted. No Response<br><br>Funding: Not received	Single Blind Parallel RCT<br><br>Power Calculations: *Yes – 20 in each group required to account for dropouts*<br><br>Adherence: *Not reported*<br><br>Treatment Fidelity: *Participants had supervised sessions. Not reported whether the individuals delivering the interventions were audited*	*Description:*<br>*General Population*<br><br>Age (mean ± SD):<br>*Gp1. 38.7* ± 7.9; *Gp2. 35.9* ± 9.8; Gp3. *44.6* ± 4.3<br><br>Sample Size (% F):<br>*60 (not reported)*<br><br>Inclusion Criteria:<br>• Aged 18 – 50<br>> 3 months neck pain	***1.*** Pilates Group - (20) - ***Pilates + Another Intervention***<br><br>***Materials:*** *Written information leaflet with exercises, information about chronic neck pain, anatomy of the spine and postural advice*<br>***Who:*** *Not reported* ***How:*** *One to one supervised session's + HEP* ***Where:*** *Unclear + Home*<br>***What:*** *(1) Hot pack, ultrasound, TEN's (2) Pilates*<br>***When and How Much:*** *Duration Supervised sessions – 3 weeks, HEP – 6 weeks; Frequency – Supervised sessions - five per week, HEP – once per day; Intensity – (2) 2x10* ***Tailoring & Modifications:*** *Unclear whether exercise dosage individualised; Modifications – not reported*<br><br>***2.*** Yoga Group - (20) - ***Yoga + Another Intervention***<br><br>***Materials:*** *Written information leaflet with exercises, information about chronic neck pain, anatomy of the spine and postural advice*<br>***Who:*** *Not reported* ***How:*** *One to one supervised session's + HEP* ***Where:*** *Unclear + Home*<br>***What:*** *(1) Hot pack, ultrasound, TEN's (2) Yoga*<br>***When and How Much:*** *Duration Supervised sessions – 3 weeks, HEP – 6 weeks; Frequency – Supervised sessions - five per week, HEP – once per day; Intensity – (2) 2x10* ***Tailoring & Modifications:*** *Unclear whether exercise dosage individualised; Modifications – not reported*<br><br>***3.*** Isometrics Group - (20) - ***Pillar Exercises + Another Intervention***<br><br>***Materials:*** *Written information leaflet with exercises, information about chronic neck pain, anatomy of the spine and postural advice*<br>***Who:*** *Not reported* ***How:*** *One to one supervised session's + HEP* ***Where:*** *Unclear + Home*<br>***What:*** *(1) Hot pack, ultrasound, TEN's (2) Cervical isometric flexion/lateral flexion in sitting using hand as resistance*<br>***When and How Much:*** *Duration Supervised sessions – 3 weeks, HEP – 6 weeks; Frequency – Supervised sessions - five per week, HEP – once per day; Intensity – (2) 2x30, 5 second holds* ***Tailoring & Modifications:*** *Unclear whether exercise dosage individualised; Modifications – not reported*	Pain:<br>*SF-MPQ*<br>*VAS*<br><br>Disability:<br>*NDI*<br><br>*Change in semispinalis capitis muscle thickness Primary Outcome Measure*<br><br>Follow Up Periods<br>*6 weeks‡*<br><br>Summary Results<br>Pain:<br>*No difference in improvement ratios between Gp3 and Gp1/2 (p > 0.05)*<br><br>Disability:<br>*No difference in improvement ratios between Gp3 and Gp1/2 (p > 0.05)*

Abbreviations:

RCT – Randomised Controlled Trial, SD- Standard Deviation, F – Female, HEP – Home Exercise Programme, NRS – Numeric Rating Scale, NDI – Neck Disability Index, MTrP – Myofascial Trigger POINTS, RM – Repetition Maximum, VAS – Visual Analogue Scale, mmHg – Millimeter of Mercury, Gp – Group, TENs – Transcutaneous Electrical Nerve Stimulation, CCF – Craniocervical Flexion, MVC – Maximal Voluntary Contraction, PSFS – Patient Specific Function Scale, MSK – Musculoskeletal, RPE – Rated of Perceived Exertion, BPM – Beats per Minute

Footnotes:

*No other details provided by authors, † Primary Outcome Measure, ‡ Primary End Point, § Comparator does not meet inclusion criteria therefore treatment arm excluded from synthesis,¶ imputation method for mean and variance values, # insufficient information for imputation methods, ‖ **Chiu., 2005** - Area of Strategic Development Fund of the Hong Kong Polytechnic University, and the Health Services Research fund of the Hong Kong Government (HSRF 821017); **Falla., 2013** - Danish Medical Research Council and Gigtforeningen Denmark; **Jordan., 1998** - Danish Medical Research Council, the Danish Arthritic Association, the Medical Research Fund for Copenhagen, the Faroe Islands and Greenland, the Foundation for Chiropractic Research and Education, and The Fund to Promote Chiropractic Research and Postgraduate Education; **Li., 2017** - Grant of National Science Foundation of China (81171469 and 81671088); **O'Leary., 2007** - Physiotherapy Research Foundation (No. 009/02) and the National Health and Medical Research Council (NHMRC) (ID 252771) of Australia. Deborah Falla was supported by a Fellowship awarded by the NHMRC of Australia (ID 351678) and Paul Hodges was supported by an NHMRC Principal Research Fellowship (ID 401599); **Randlov., 1998** - Danish Rheumatism Association; **Rudolfsson., 2014** - Alfta Research Foundation, grants from the Swedish Council for Working Life and Social Research (2006-1162) and Länsförsäkringar Forskning och Framtid (51-1010/06); **Viljanen., 2003** - Finnish work environment fund (project No 96243); **Waling., 2000** - The Swedish Council for Work Life Research, Dnr 94-0315; **Ylinen., 2003** - Social Insurance Institution, Helsinki, Finland.

References:

13. Chung S, Jeong YG. Effects of the craniocervical flexion and isometric neck exercise compared in patients with chronic neck pain: A randomized controlled trial. *Physiother Theory Pract* 2018;34(12):916-25. doi: 10.1080/09593985.2018.1430876 [published Online First: 2018/01/25]
14. Jordan A, Bendix T, Nielsen H, et al. Intensive training, physiotherapy, or manipulation for patients with chronic neck pain. A prospective, single-blinded, randomized clinical trial. *Spine (Phila Pa 1976)* 1998;23(3):311-8; discussion 19. doi: 10.1097/00007632-199802010-00005 [published Online First: 1998/03/21]
49. Waling K, Jarvholm B, Sundelin G. Effects of training on female trapezius Myalgia: An intervention study with a 3-year follow-up period. *Spine (Phila Pa 1976)* 2002;27(8):789-96. doi: 10.1097/00007632-200204150-00002 [published Online First: 2002/04/06]
51. Bobos P, Billis E, Papanikolaou DT, et al. Does Deep Cervical Flexor Muscle Training Affect Pain Pressure Thresholds of Myofascial Trigger Points in Patients with Chronic Neck Pain? A Prospective Randomized Controlled Trial. *Rehabil Res Pract* 2016;2016:6480826. doi: 10.1155/2016/6480826 [published Online First: 2016/12/19]
54. Ylinen J, Hakkinen A, Nykanen M, et al. Neck muscle training in the treatment of chronic neck pain: a three-year follow-up study. *Eura Medicophys* 2007;43(2):161-9. [published Online First: 2007/05/26]

57. Chiu TT, Hui-Chan CW, Chein G. A randomized clinical trial of TENS and exercise for patients with chronic neck pain. *Clin Rehabil* 2005;19(8):850-60. doi: 10.1191/0269215505cr920oa [published Online First: 2005/12/06]
59. Javanshir K, Amiri M, Mohseni Bandpei MA, et al. The effect of different exercise programs on cervical flexor muscles dimensions in patients with chronic neck pain. *J Back Musculoskelet Rehabil* 2015;28(4):833-40. doi: 10.3233/BMR-150593 [published Online First: 2015/03/31]
60. O'Leary S, Falla D, Hodges PW, et al. Specific therapeutic exercise of the neck induces immediate local hypoalgesia. *J Pain* 2007;8(11):832-9. doi: 10.1016/j.jpain.2007.05.014 [published Online First: 2007/07/24]
61. Rudolfsson T, Djupsjobacka M, Hager C, et al. Effects of neck coordination exercise on sensorimotor function in chronic neck pain: a randomized controlled trial. *J Rehabil Med* 2014;46(9):908-14. doi: 10.2340/16501977-1869 [published Online First: 2014/09/04]
62. Yildiz TI, Turgut E, Duzgun I. Neck and Scapula-Focused Exercise Training on Patients With Nonspecific Neck Pain: A Randomized Controlled Trial. *J Sport Rehabil* 2018;27(5):403-12. doi: 10.1123/jsr.2017-0024 [published Online First: 2017/06/13]
63. Borisut S, Vongsirinavarat M, Vachalathiti R, et al. Effects of strength and endurance training of superficial and deep neck muscles on muscle activities and pain levels of females with chronic neck pain. *J Phys Ther Sci* 2013;25(9):1157-62. doi: 10.1589/jpts.25.1157 [published Online First: 2013/11/22]
64. Falla D, Lindstrom R, Rechter L, et al. Effectiveness of an 8-week exercise programme on pain and specificity of neck muscle activity in patients with chronic neck pain: a randomized controlled study. *European journal of pain (London, England)* 2013;17(10):1517-28. doi: 10.1002/j.1532-2149.2013.00321.x [published Online First: 2013/05/08]
65. Li X, Lin C, Liu C, et al. Comparison of the effectiveness of resistance training in women with chronic computer-related neck pain: a randomized controlled study. *Int Arch Occup Environ Health* 2017;90(7):673-83. doi: 10.1007/s00420-017-1230-2 [published Online First: 2017/05/22]
66. Viljanen M, Malmivaara A, Uitti J, et al. Effectiveness of dynamic muscle training, relaxation training, or ordinary activity for chronic neck pain: randomised controlled trial. *BMJ* 2003;327(7413):475. doi: 10.1136/bmj.327.7413.475 [published Online First: 2003/08/30]
67. Suvarnnato T, Puntumetakul R, Uthaikhup S, et al. Effect of specific deep cervical muscle exercises on functional disability, pain intensity, craniovertebral angle, and neck-muscle strength in chronic mechanical neck pain: a randomized controlled trial. *J Pain Res* 2019;12:915-25. doi: 10.2147/JPR.S190125 [published Online First: 2019/03/19]
68. Shiravi S, Letafatkar A, Bertozzi L, et al. Efficacy of Abdominal Control Feedback and Scapula Stabilization Exercises in Participants With Forward Head, Round Shoulder Postures and Neck Movement Impairment. *Sports Health* 2019;11(3):272-79. doi: 10.1177/1941738119835223 [published Online First: 2019/04/24]
69. Gupta S, Sharma S, Kataria C. Craniocervical Flexors Endurance Training: Treatment Approach for Cervical Spondylosis. *Indian Journal of Physiotherapy & Occupational Therapy* 2010;4(2):76-81.
70. Gupta BD, Aggarwal S, Gupta B, et al. Effect of Deep Cervical Flexor Training vs. Conventional Isometric Training on Forward Head Posture, Pain, Neck Disability Index In Dentists Suffering from Chronic Neck Pain. *J Clin Diagn Res* 2013;7(10):2261-4. doi: 10.7860/JCDR/2013/6072.3487 [published Online First: 2013/12/04]
71. Hingarajia DD, Sushant; Tejwani, Nishant. Effect of Deep Cranio-cervical Flexors Training Over Isometric Neck Exercises in Chronic Neck Pain and Disability. *Indian Journal of Physiotherapy & Occupational Therapy* 2012;6(4):48-53.
72. Gallego Izquierdo T, Pecos-Martin D, Lluch Girbes E, et al. Comparison of cranio-cervical flexion training versus cervical proprioception training in patients with chronic neck pain: A randomized controlled clinical trial. *J Rehabil Med* 2016;48(1):48-55. doi: 10.2340/16501977-2034 [published Online First: 2015/12/15]
73. Kaur A, Mali K, Mitra M. To Compare the Immediate Effects of Active Cranio Cervical Flexion Exercise Versus Passive Mobilization of Upper Cervical Spine on Pain, Range of Motion and Cranio Cervical Flexion Test in Patients with Chronic Neck Pain. *Indian Journal of Physiotherapy & Occupational Therapy* 2018;12(3):22-27.
74. Kim JY, Kwag KI. Clinical effects of deep cervical flexor muscle activation in patients with chronic neck pain. *J Phys Ther Sci* 2016;28(1):269-73. doi: 10.1589/jpts.28.269 [published Online First: 2016/03/10]
75. Kwan-Woo L, Won-Ho K. Effect of thoracic manipulation and deep craniocervical flexor training on pain, mobility, strength, and disability of the neck of patients with chronic nonspecific neck pain: a randomized clinical trial. *J Phys Ther Sci* 2016;28(1):175-80. doi: 10.1589/jpts.28.175 [published Online First: 2016/03/10]
76. Randlov A, Ostergaard M, Manniche C, et al. Intensive dynamic training for females with chronic neck/shoulder pain. A randomized controlled trial. *Clin Rehabil* 1998;12(3):200-10. doi: 10.1191/026921598666881319 [published Online First: 1998/08/04]
77. Khan M, Soomro RR, Ali SS. The effectiveness of isometric exercises as compared to general exercises in the management of chronic non-specific neck pain. *Pak J Pharm Sci* 2014;27(5 Suppl):1719-22. [published Online First: 2014/09/30]
78. Ulug N, Yilmaz OT, Kara M, et al. Effects of Pilates and yoga in patients with chronic neck pain: A sonographic study. *J Rehabil Med* 2018;50(1):80-85. doi: 10.2340/16501977-2288 [published Online First: 2017/11/22]
