# Supplementary material for: Effectiveness and optimal dosage of exercise training for chronic non-specific neck pain: A systematic review with a narrative synthesis
Source: PLoS One. 2020 Jun 10;15(6):e0234511. doi: 10.1371/journal.pone.0234511 (PMC7286530; doi:10.1371/journal.pone.0234511)
Supplement: S8 Appendix — (PDF) [file pone.0234511.s008.pdf]

S8 Appendix Means, Standard Deviations and Standardised Mean Differences for all exercise training programmes

	Comparator	Exercise Training Programme			Comparator			Effect Estimate (Std. Mean Difference IV, Random) [95%CIs]
		Mean	SD	Total	Mean	SD	Total	
**Motor Control**								
Pain								
Immediate Term: VAS								
Kaur et al., 2018 [73]	Manual Therapy	3.41	0.94	17	4.53	1.07	17	-1.09 [-1.81,-0.36]
Immediate Term: Max VAS								
Izquierdo et al., 2016 [72]	Proprioceptive Training	5.84	2.12	14	4.65	1.48	14	0.63 [-0.13, 1.39]
Immediate Term: Min VAS								
Izquierdo et al., 2016 [72]	Proprioceptive Training	2.58	2.47	14	3.35	1.65	14	-0.36 [-1.10, 0.39]
Immediate Term: VAS at Rest								
Izquierdo et al., 2016 [72]	Proprioceptive Training	0.33	0.58	14	1.34	2.41	14	-0.56 [-1.32, 0.20]
O'Leary et al., 2007 [60]	Segmental	0.57	1.01	24	0.85	1.43	24	-0.22 [-0.79, 0.35]
Immediate Term: VAS on Active Movements								
O'Leary et al., 2007 [60]	Segmental	0.98	0.92	24	1.42	1.07	24	-0.43 [-1.01, 0.14]
Short Term: VAS/NRS								
Borisut et al., 2013 [63]	No Treatment	43.04	18.56	25	61.32	11.29	25	-1.17 [-1.78,-0.57]
Kwan-Woo et al., 2016 [75]	General AROM	2.00	0.50	15	3.80	0.40	15	-3.87 [-5.14,-2.60]
Chung et al., 2018 [13]	Pillar	2.72	1.28	22	3.97	0.87	19	-1.10 [-1.77,-0.44]
Gupta et al., 2013 [70]	Pillar	3.80	0.68	15	4.73	0.70	15	-1.31 [-2.11,-0.51]
Borisut et al., 2013 [63]	Segmental	43.04	18.56	25	38.68	9.49	25	0.29 [-0.27, 0.85]
Javanshir et al., 2015 [59]	Segmental	2.27	1.51	30	2.75	1.41	30	-0.32 [-0.83, 0.19]
Borisut et al., 2013 [63]	Motor Control + Segmental	43.04	18.56	25	16.88	7.75	25	1.81 [1.14, 2.48]
Kim 2016	Pillar + Another Intervention	1.70	1.80	14	3.10	1.90	14	-0.73 [-1.50, 0.03]
Suvrannato et al., 2019 [67]	Usual Care	2.93	0.16	18	3.06	0.18	18	-0.75 [-1.42,-0.07]
Suvrannato et al., 2019 [67]	Pillar (Therapist Assisted)	2.93	0.16	18	2.53	0.17	18	2.37 [1.50, 3.24]
Short Term: Max VAS								
Izquierdo et al., 2016 [72]	Proprioceptive Training	2.19	1.98	14	1.93	2.02	14	0.13 [-0.62, 0.87]
Short Term: Min VAS								
Izquierdo et al., 2016 [72]	Proprioceptive Training	0.40	0.83	14	1.09	1.66	14	-0.51 [-1.27, 0.24]
Short Term: VAS at Rest								
Izquierdo et al., 2016 [72]	Proprioceptive Training	0.09	0.21	14	0.23	0.58	14	-0.31 [-1.06, 0.43]
Intermediate Term: NRS								
Suvrannato et al., 2019 [67]	Usual Care	3.27	0.19	18	3.37	0.21	18	-0.49 [-1.15, 0.18]
Suvrannato et al., 2019 [67]	Pillar (Therapist Assisted)	3.27	0.19	18	2.97	0.19	18	2.47 [1.58, 3.36]
Disability								
Immediate Term: NDI								
Izquierdo et al., 2016 [72]	Proprioceptive Training	6.64	2.61	14	6.21	2.35	14	0.17 [-0.57, 0.91]
Short Term: NDI								
Borisut et al., 2013 [63]	No Treatment	14.41	4.94	25	33.86	5.04	25	-3.84 [-4.80,-2.88]
Kwan-Woo et al., 2016 [75]	General AROM	10.70	1.80	15	20.40	2.50	15	-4.33 [-5.71,-2.96]
Chung et al., 2018 [13]	Pillar	9.05	5.09	22	14.21	5.09	19	-0.99 [-1.65,-0.34]
Gupta et al., 2013 [70]	Pillar	14.33	1.23	15	16.33	1.05	15	-1.70 [-2.55,-0.85]

	Comparator	Exercise Training Programme			Comparator			Effect Estimate (Std. Mean Difference IV, Random) [95%CIs]
		Mean	SD	Total	Mean	SD	Total	
Borisut et al., 2013 [63]	Segmental	14.41	4.94	25	14.69	4.64	25	-0.06 [-0.61, 0.50]
Javanshir et al., 2015 [59]	Segmental	15.20	9.16	30	19.01	10.10	30	-0.39 [-0.90, 0.12]
Izquierdo et al., 2016 [72]	Proprioceptive Training	4.46	2.02	14	4.14	2.62	14	0.13 [-0.61, 0.87]
Borisut et al., 2013 [63]	Motor Control + Segmental	14.41	4.94	25	15.71	3.01	25	-0.31 [-0.87, 0.25]
Kim 2016	Pillar + Another Intervention	15.40	2.80	14	17.40	3.90	14	-0.57 [-1.33, 0.19]
Suvrannato et al., 2019 [67]	Usual Care	15.68	0.90	18	21.12	0.92	18	-5.84 [-7.74,-4.27]
Suvrannato et al., 2019 [67]	Pillar (Therapist Assisted)	15.68	0.90	18	13.07	0.91	18	2.82 [1.87, 3.77]
Intermediate Term: NDI								
Suvrannato et al., 2019 [67]	Usual Care	16.62	1.00	18	21.69	1.10	18	-4.72 [-6.04,-3.39]
Suvrannato et al., 2019 [67]	Pillar (Therapist Assisted)	16.62	1.00	18	12.97	1.03	18	3.53 [2.44, 4.60]
**Pillar**								
Pain								
Short Term: VAS								
Li et al., 2017 [65] (Progressive Load)	Education	2.39	0.82	38	4.87	0.88	36	-2.89 [-3.55,-2.23]
Li et al., 2017 [65] (Fixed Load)	Education	2.96	0.70	35	4.87	0.88	36	-2.37 [-2.99,-1.76]
Chung et al., 2018 [13]	Motor Control	3.97	0.87	19	2.72	1.28	22	1.10 [0.44, 1.77]
Gupta et al., 2013 [70]	Motor Control	4.73	0.70	15	3.80	0.68	15	1.31 [0.51, 2.11]
Li et al., 2017 [65] (Progressive Load)	Pillar (Fixed Load)	2.39	0.82	38	2.96	0.70	35	-0.74 [-1.21,-0.26]
Li et al., 2017 [65] (Fixed Load)	Pillar (Progressive Load)	2.96	0.70	35	2.39	0.82	38	0.74 [0.26, 1.21]
Hingarajia et al., 2012 [71]	Motor Control + Pillar	2.03	1.00	25	1.39	0.90	25	0.66 [0.09, 1.23]
Short Term: VAS at Rest								
Gupta et al., 2010 [69]	Motor Control + Segmental + Pillar	2.00	0.83	12	0.08	0.29	12	2.98 [1.76, 4.20]
Short Term: VAS on Activity								
Gupta et al., 2010 [69]	Motor Control + Segmental + Pillar	3.75	0.87	12	1.42	0.79	12	2.71 [1.55, 3.87]
Intermediate Term: VAS								
Li et al., 2017 [65] (Progressive Load)	Education	1.92	0.90	38	5.10	0.95	36	-3.40 [-4.13,-2.68]
Li et al., 2017 [65] (Fixed Load)	Education	2.51	0.88	35	5.10	0.95	36	-2.80 [-3.46,-2.13]
Li et al., 2017 [65] (Progressive Load)	Pillar (Fixed Load)	1.92	0.90	38	2.51	0.88	35	-0.66 [-1.13,-0.18]
Li et al., 2017 [65] (Fixed Load)	Pillar (Progressive Load)	2.51	0.88	35	1.92	0.90	38	0.66 [0.18, 1.13]
Disability								
Short Term: NDI								
Li et al., 2017 [65] (Progressive Load)	Education	15.72	4.83	38	27.42	6.37	36	-2.06 [-2.62,-1.49]
Li et al., 2017 [65] (Fixed Load)	Education	16.87	5.10	35	27.42	6.37	36	-1.81 [-2.36,-1.25]
Chung et al., 2018 [13]	Motor Control	14.21	5.09	19	9.05	5.09	22	0.99 [0.34, 1.65]
Gupta et al., 2013 [70]	Motor Control	16.33	1.05	15	14.33	1.23	15	1.70 [0.85, 2.55]
Li et al., 2017 [65] (Progressive Load)	Pillar (Fixed Load)	15.72	4.83	38	16.87	5.10	35	-0.23 [-0.69, 0.23]
Li et al., 2017 [65] (Fixed Load)	Pillar (Progressive Load)	16.87	5.10	35	15.72	4.83	38	0.23 [-0.23, 0.69]
Hingarajia et al., 2012 [71]	Motor Control + Pillar	5.32	2.50	25	2.64	1.70	25	1.23 [0.63, 1.84]
Gupta et al., 2010 [69]	Motor Control + Segmental + Pillar	20.35	8.21	12	6.28	5.92	12	1.90 [0.91, 2.89]
Intermediate Term: NDI								
Li et al., 2017 [65] (Progressive Load)	Education	14.93	4.85	38	26.55	5.35	36	-2.25 [-2.84,-1.67]
Li et al., 2017 [65] (Fixed Load)	Education	15.80	4.77	35	26.55	5.35	36	-2.10 [-2.68,-1.51]
Li et al., 2017 [65] (Progressive Load)	Pillar (Fixed Load)	14.93	4.85	38	15.80	4.77	35	-0.18 [-0.64, 0.28]

	Comparator	Exercise Training Programme			Comparator			Effect Estimate (Std. Mean Difference IV, Random) [95%CIs]
		Mean	SD	Total	Mean	SD	Total	
Li et al., 2017 [65] (Fixed Load)	Pillar (Progressive Load)	15.80	4.77	35	14.93	4.85	38	0.18 [-0.28, 0.64]
**Segmental**								
Pain								
Immediate Term: VAS at Rest								
O'Leary et al., 2007 [60]	Motor Control	0.85	1.43	24	0.57	1.01	24	0.22 [-0.35, 0.79]
Immediate Term: VAS on Active Movements								
O'Leary et al., 2007 [60]	Motor Control	1.42	1.07	24	0.98	0.92	24	0.43 [-0.14, 1.01]
Short Term: VAS/NRS								
Borisut et al., 2013 [63]	No Treatment	38.68	9.49	25	61.32	11.29	25	-2.14 [-2.84,-1.43]
Borisut et al., 2013 [63]	Motor Control	38.68	9.49	25	43.04	18.56	25	-0.29 [-0.85, 0.27]
Javanshir et al., 2015 [59]	Motor Control	2.75	1.41	30	2.27	1.51	30	0.32 [-0.19, 0.83]
Borisut et al., 2013 [63]	Motor Control + Segmental	38.68	9.49	25	16.88	7.75	25	2.48 [1.73, 3.23]
Disability								
Short Term: NDI								
Borisut et al., 2013 [63]	No Treatment	14.69	4.64	25	33.86	5.04	25	-3.90 [-4.86,-2.93]
Borisut et al., 2013 [63]	Motor Control	14.69	4.64	25	14.41	4.94	25	0.06 [-0.50, 0.61]
Javanshir et al., 2015 [59]	Motor Control	19.01	10.1	30	15.20	9.16	30	0.39 [-0.12, 0.90]
Borisut et al., 2013 [63]	Motor Control + Segmental	14.69	4.64	25	15.71	3.01	25	-0.26 [-0.81, 0.30]
**Upper Limb**								
Pain								
Short Term: VAS General								
Shiravi et al., 2019 [68]	No Treatment	3.10	1.04	44	6.00	1.23	44	-2.52 [-3.09,-1.96]
Waling et al., 2002 [49]	Aerobic + Upper Limb	22.00	18.00	29	31.00	17.00	28	-0.51 [-1.03, 0.02]
Waling et al., 2002 [49]	Body Awareness Training	22.00	18.00	29	30.00	17.00	25	-0.45 [-0.99, 0.09]
Waling et al., 2002 [49]	Education and Stress Reduction	22.00	18.00	29	38.00	24.00	20	-0.76 [-1.35,-0.17]
Short Term: VAS Present								
Waling et al., 2002 [49]	Aerobic + Upper Limb	11.00	16.00	29	19.00	14.00	28	-0.52 [-1.05, 0.00]
Waling et al., 2002 [49]	Body Awareness Training	11.00	16.00	29	24.00	25.00	25	-0.62 [-1.17,-0.07]
Waling et al., 2002 [49]	Education and Stress Reduction	11.00	16.00	29	30.00	21.00	20	-1.03 [-1.64,-0.42]
Short Term: VAS Worst								
Waling et al., 2002 [49]	Aerobic + Upper Limb	54.00	27.00	29	59.00	21.00	28	-0.20 [-0.72, 0.32]
Waling et al., 2002 [49]	Body Awareness Training	54.00	27.00	29	67.00	19.00	25	-0.54 [-1.09, 0.00]
Waling et al., 2002 [49]	Education and Stress Reduction	54.00	27.00	29	74.00	19.00	20	-0.82 [-1.41,-0.22]
Intermediate Term: VAS Worst								
Waling et al., 2002 [49]	Aerobic + Upper Limb	60.00	28.00	24	57.00	19.00	24	0.12 [-0.44, 0.69]
Waling et al., 2002 [49]	Body Awareness Training	60.00	28.00	24	70.00	19.00	24	-0.41 [-0.98, 0.16]
Waling et al., 2002 [49]	Education and Stress Reduction	60.00	28.00	24	63.00	18.00	15	-0.12 [-0.76, 0.53]
Intermediate Term: VAS Present								
Waling et al., 2002 [49]	Aerobic + Upper Limb	20.00	21.00	24	20.00	18.00	24	0.00 [-0.57, 0.57]
Waling et al., 2002 [49]	Body Awareness Training	20.00	21.00	24	35.00	29.00	24	-0.58 [-1.16, 0.00]
Waling et al., 2002 [49]	Education and Stress Reduction	20.00	21.00	24	25.00	19.00	15	-0.24 [-0.89, 0.41]
Intermediate Term: VAS General								
Waling et al., 2002 [49]	Aerobic + Upper Limb	28.00	19.00	24	26.00	16.00	24	0.11 [-0.45, 0.68]

	Comparator	Exercise Training Programme			Comparator			Effect Estimate (Std. Mean Difference IV, Random) [95%CIs]
		Mean	SD	Total	Mean	SD	Total	
Waling et al., 2002 [49]	Body Awareness Training	28.00	19.00	24	31.00	22.00	24	-0.14 [-0.71, 0.42]
Waling et al., 2002 [49]	Education and Stress Reduction	28.00	19.00	24	29.00	18.00	15	-0.05 [-0.70, 0.59]
Long Term: VAS Worst								
Waling et al., 2002 [49]	Aerobic + Upper Limb	61.00	27.00	34	58.00	27.00	34	0.11 [-0.37, 0.59]
Waling et al., 2002 [49]	Body Awareness Training	61.00	27.00	34	57.00	28.00	31	0.14 [-0.34, 0.63]
Waling et al., 2002 [49]	Education and Stress Reduction	61.00	27.00	34	58.00	29.00	27	0.11 [-0.40, 0.61]
Long Term: VAS Present								
Waling et al., 2002 [49]	Aerobic + Upper Limb	31.00	27.00	34	22.00	26.00	34	0.34 [-0.14, 0.81]
Waling et al., 2002 [49]	Body Awareness Training	31.00	27.00	34	27.00	27.00	31	0.15 [-0.34, 0.63]
Waling et al., 2002 [49]	Education and Stress Reduction	31.00	27.00	34	16.00	19.00	27	0.62 [0.10, 1.14]
Long Term: VAS General								
Waling et al., 2002 [49]	Aerobic + Upper Limb	32.00	22.00	34	29.00	19.00	34	0.14 [-0.33, 0.62]
Waling et al., 2002 [49]	Body Awareness Training	32.00	22.00	34	29.00	21.00	31	0.14 [-0.35, 0.63]
Waling et al., 2002 [49]	Education and Stress Reduction	32.00	22.00	34	20.00	18.00	27	0.58 [0.07, 1.10]
**Motor Control + Pillar**								
Pain								
Short Term: VAS								
Hingarajia et al., 2012 [71]	Pillar	1.39	0.90	25	2.03	1.00	25	-0.66 [-1.23,-0.09]
Disability								
Short Term: NDI								
Hingarajia et al., 2012 [71]	Pillar	2.64	1.70	25	5.32	2.50	25	-1.23 [-1.84,-0.63]
**Motor Control + Segmental**								
Pain								
Short Term: VAS								
Borisut et al., 2013 [63]	No Treatment	16.88	7.75	25	61.32	11.29	25	-4.52 [-5.59,-3.44]
Falla et al., 2013 [64]	No Treatment	3.60	2.40	22	4.90	2.30	20	-0.54 [-1.16, 0.08]
Borisut et al., 2013 [63]	Motor Control	16.88	7.75	25	43.04	18.56	25	-1.81 [-2.48,-1.14]
Borisut et al., 2013 [63]	Segmental	16.88	7.75	25	38.68	9.49	25	-2.48 [-3.23,-1.73]
Disability								
Short Term: NDI								
Borisut et al., 2013 [63]	No Treatment	15.71	3.01	25	33.86	5.04	25	-4.30 [-5.34,-3.26]
Falla et al., 2013 [64]	No Treatment	14.10	6.60	22	16.60	7.40	20	-0.35 [-0.96, 0.26]
Borisut et al., 2013 [63]	Motor Control	15.71	3.01	25	14.41	4.94	25	0.31 [-0.25, 0.87]
Borisut et al., 2013 [63]	Segmental	15.71	3.01	25	14.69	4.64	25	0.26 [-0.30, 0.81]
Short Term: PSFS								
Falla et al., 2013 [64]	No Treatment	-5.60	2.20	22	-3.9	1.70	20	-0.84 [-1.48,-0.21]
**Motor Control + Segmental + Pillar**								
Pain								
Short Term: VAS on Activity								
Gupta et al., 2010 [69]	Pillar	1.42	0.79	12	3.75	0.87	12	-2.71 [-3.87,-1.55]
Short Term: VAS at Rest								
Gupta et al., 2010 [69]	Pillar	0.08	0.29	12	2.00	0.83	12	-2.98 [-4.20,-1.76]
Disability								

	Comparator	Exercise Training Programme Mean	SD	Total	Comparator Mean	SD	Total	Effect Estimate (Std. Mean Difference IV, Random) [95%CIs]
Short Term: NDI								
Gupta et al., 2010 [69]	Pillar	6.28	5.92	12	20.35	8.21	12	-1.9 [-2.89,-0.91]
**Motor Control + Segmental + Another Intervention**								
Pain								
Short Term: NRS								
Chiu et al., 2005 [57]	Another Intervention	3.04	1.87	67	3.96	2.18	78	-0.45 [-0.78,-0.12]
Chiu et al., 2005 [57]	TENs + Another Intervention	3.04	1.87	67	4.36	2.04	73	-0.67 [-1.01,-0.33]
Short Term: NRS at Best								
Bobos et al., 2016 [51]	Another Intervention	0.20	0.41	20	0.75	0.91	20	-0.76 [-1.41,-0.12]
Bobos et al., 2016 [51]	General AROM + Another Intervention	0.20	0.41	20	0.85	0.93	20	-0.89 [-1.54,-0.23]
Short Term: NRS at Worst								
Bobos et al., 2016 [51]	Another Intervention	3.60	1.19	20	3.90	1.80	20	-0.19 [-0.81, 0.43]
Bobos et al., 2016 [51]	General AROM + Another Intervention	3.60	1.19	20	4.30	2.41	20	-0.36 [-0.99, 0.26]
Short Term: NRS Now								
Bobos et al., 2016 [51]	Another Intervention	1.55	1.19	20	2.80	1.64	20	-0.86 [-1.51,-0.20]
Bobos et al., 2016 [51]	General AROM + Another Intervention	1.55	1.19	20	2.10	1.94	20	-0.33 [-0.96, 0.29]
Intermediate Term: NRS								
Chiu et al., 2005 [57]	Another Intervention	3.06	2.09	67	3.61	2.06	78	-0.26 [-0.59, 0.06]
Chiu et al., 2005 [57]	TENs + Another Intervention	3.06	2.09	67	3.40	2.35	73	-0.15 [-0.48, 0.18]
Disability								
Short Term: NDI								
Bobos et al., 2016 [51]	Another Intervention	4.95	2.70	20	8.00	2.66	20	-1.12 [-1.79,-0.44]
Bobos et al., 2016 [51]	General AROM + Another Intervention	4.95	2.70	20	8.85	4.50	20	-1.03 [-1.69,-0.37]
Short Term: Northwick Park Neck Pain Questionnaire								
Chiu et al., 2005 [57]	Another Intervention	1.00	0.42	67	1.13	0.56	78	-0.26 [-0.59, 0.07]
Chiu et al., 2005 [57]	TENs + Another Intervention	1.00	0.42	67	1.17	0.51	73	-0.36 [-0.69,-0.03]
Intermediate Term: Northwick Park Neck Pain Questionnaire								
Chiu et al., 2005 [57]	Another Intervention	1.02	0.58	67	1.16	0.56	78	-0.24 [-0.57, 0.08]
Chiu et al., 2005 [57]	TENs + Another Intervention	1.02	0.58	67	1.19	0.53	73	-0.30 [-0.64, 0.03]
**Segmental + Upper Limb**								
Pain								
Short Term: 11 Point Box Scale								
Randlov et al., 1998 [76] (Intense)	Segmental + Upper Limb Exercise (Light)	10.00*	NA	27	9.00*	NA	25	p > 0.05
Randlov et al., 1998 [76] (Light)	Segmental + Upper Limb Exercise (Intense)	9.00*	NA	25	10.00*	NA	27	p > 0.05
Intermediate Term: 11 Point Box Scale								
Randlov et al., 1998 [76] (Intense)	Segmental + Upper Limb Exercise (Light)	8.00*	NA	23	12.00*	NA	20	p > 0.05
Randlov et al., 1998 [76] (Light)	Segmental + Upper Limb Exercise (Intense)	12.00*	NA	20	8.00*	NA	23	p > 0.05
Long Term: 11 Point Box Scale								
Randlov et al., 1998 [76] (Intense)	Segmental + Upper Limb Exercise (Light)	9.00*	NA	21	12.00*	NA	20	p > 0.05
Randlov et al., 1998 [76] (Light)	Segmental + Upper Limb Exercise (Intense)	12.00*	NA	20	9.00*	NA	21	p > 0.05
Disability								
Short Term: ADL Questionnaire								
Randlov et al., 1998 [76] (Intense)	Segmental + Upper Limb Exercise (Light)	10.00*	NA	27	12.00*	NA	25	p = 0.99

	Comparator	Exercise Training Programme			Comparator			Effect Estimate (Std. Mean Difference IV, Random) [95%CIs]
		Mean	SD	Total	Mean	SD	Total	
Randlov et al., 1998 [76] (Light)	Segmental + Upper Limb Exercise (Intense)	12.00*	NA	25	10.00*	NA	27	p = 0.99
<u>Intermediate Term: ADL Questionnaire</u>								
Randlov et al., 1998 [76] (Intense)	Segmental + Upper Limb Exercise (Light)	10.00*	NA	23	12.00*	NA	20	p = 0.43
Randlov et al., 1998 [76] (Light)	Segmental + Upper Limb Exercise (Intense)	12.00*	NA	20	10.00*	NA	23	p = 0.43
<u>Long Term: ADL Questionnaire</u>								
Randlov et al., 1998 [76] (Intense)	Segmental + Upper Limb Exercise (Light)	10.00*	NA	21	12.00*	NA	20	p = 0.26
Randlov et al., 1998 [76] (Light)	Segmental + Upper Limb Exercise (Intense)	12.00*	NA	20	10.00*	NA	21	p = 0.26
**Pillar + Another Intervention**								
Pain								
<u>Short Term: VAS</u>								
Khan et al., 2014 [77]	Another Intervention	5.96	2.23	34	6.23	1.31	34	-0.15 [-0.62, 0.33]
Ulug et al., 2018 [78]	Pilates + Another Intervention	2.50	2.30	18	1.70	1.80	20	0.38 [-0.26, 1.03]
Ulug et al., 2018 [78]	Yoga + Another Intervention	2.50	2.30	18	1.40	2.00	18	0.50 [-0.17, 1.16]
<u>Short Term: SF-MPQ</u>								
Ulug et al., 2018 [78]	Pilates + Another Intervention	2.20	3.70	18	2.40	3.50	20	-0.05 [-0.69, 0.58]
Ulug et al., 2018 [78]	Yoga + Another Intervention	2.20	3.70	18	1.30	2.20	18	0.29 [-0.37, 0.95]
Disability								
<u>Short Term: NDI</u>								
Ulug et al., 2018 [78]	Pilates + Another Intervention	11.30	6.30	18	10.00	4.80	20	0.23 [-0.41, 0.87]
Ulug et al., 2018 [78]	Yoga + Another Intervention	11.30	6.30	18	8.20	4.80	18	0.54 [-0.13, 1.21]
<u>Short Term: Northwick Park Neck Pain Questionnaire</u>								
Khan et al., 2014 [77]	Another Intervention	11.38	2.41	34	17.54	1.43	34	-3.07 [-3.79,-2.36]
**Pillar + Upper Limb**								
Pain								
<u>Intermediate Term: NRS</u>								
Rudolfsson et al., 2014 [61]	Co-ordination Training	4.40	2.00	28	3.80	1.70	28	0.32 [-0.21, 0.85]
Rudolfsson et al., 2014 [61]	Massage	4.40	2.00	28	4.00	2.10	29	0.19 [-0.33, 0.71]
**Pillar + Upper Limb + Another Intervention**								
Pain								
<u>Intermediate Term: VAS</u>								
Ylinen et al., 2007 [54]	Segmental + Upper Limb + Another Intervention	11.00	13.68	59	15.00	15.21	57	-0.27 [-0.64, 0.09]
<u>Long Term: VAS</u>								
Ylinen et al., 2007 [54]	Segmental + Upper Limb + Another Intervention	19.00	26.60	59	20.67	23.58	57	-0.07 [-0.43, 0.30]
Disability								
<u>Short Term: Neck and Shoulder Pain and Disability Index</u>								
Ylinen et al., 2007 [54]	Segmental + Upper Limb + Another Intervention	15.67	16.72	59	20.00	15.21	57	-0.27 [-0.63, 0.10]
<u>Intermediate Term: Neck and Shoulder Pain and Disability Index</u>								
Ylinen et al., 2007 [54]	Segmental + Upper Limb + Another Intervention	13.00	18.24	59	18.67	17.49	57	-0.32 [-0.68, 0.05]
<u>Long Term: Neck and Shoulder Pain and Disability Index</u>								
Ylinen et al., 2007 [54]	Segmental + Upper Limb + Another Intervention	14.00	18.24	59	14.00	15.97	57	0.00 [-0.36, 0.36]
<u>Long Term: NDI</u>								
Ylinen et al., 2007 [54]	Segmental + Upper Limb + Another Intervention	12.67	13.68	59	13.33	10.65	57	-0.05 [-0.42, 0.31]

	Comparator	Exercise Training Programme Mean	SD	Total	Comparator Mean	SD	Total	Effect Estimate (Std. Mean Difference IV, Random) [95%CIs]
**Segmental + Upper Limb + Another Intervention**								
Pain								
Short Term: 11 Point Box Scale								
Jordan et al., 1998 [14]	Manipulation + Another Intervention	6.00*	3-9†	??	6.00*	4-7†	??	p = 0.44
Jordan et al., 1998 [14]	Passive Physiotherapy + Another Intervention	6.00*	3-9†	??	6.00*	3-8†	??	p = 0.44
Intermediate Term: 11 Point Box Scale								
Jordan et al., 1998 [14]	Manipulation + Another Intervention	4.00*	3-10†	??	6.00*	5-8†	??	p = 0.54
Jordan et al., 1998 [14]	Passive Physiotherapy + Another Intervention	4.00*	3-10†	??	4.00*	3-10†	??	p = 0.54
Intermediate Term: VAS								
Ylinen et al., 2007 [54]	Pillar + Upper Limb + Another Intervention	15.00	15.21	57	11.00	13.68	59	0.27 [-0.09, 0.64]
Long Term: 11 Point Box Scale								
Jordan et al., 1998 [14]	Manipulation + Another Intervention	6.00*	4-9†	??	6.00*	6-8†	??	p = 0.76
Jordan et al., 1998 [14]	Passive Physiotherapy + Another Intervention	6.00*	4-9†	??	8.00*	6-11†	??	p = 0.76
Long Term: VAS								
Ylinen et al., 2007 [54]	Pillar + Upper Limb + Another Intervention	20.67	23.58	57	19.00	26.60	59	0.07 [-0.30, 0.43]
Disability								
Short Term: Self Reporting Disability								
Jordan et al., 1998 [14]	Manipulation + Another Intervention	5.00*	4-7†	??	4.00*	4-5†	??	p = 0.61
Jordan et al., 1998 [14]	Passive Physiotherapy + Another Intervention	5.00*	4-7†	??	4.00*	3-6†	??	p = 0.61
Short Term: Neck and Shoulder Pain and Disability Index								
Ylinen et al., 2007 [54]	Pillar + Upper Limb + Another Intervention	20.00	15.21	57	15.67	16.72	59	0.27 [-0.10, 0.63]
Intermediate: Self Reporting Disability								
Jordan et al., 1998 [14]	Manipulation + Another Intervention	5.00*	3-7†	??	6.00*	4-7†	??	p = 0.69
Jordan et al., 1998 [14]	Passive Physiotherapy + Another Intervention	5.00*	3-7†	??	5.00*	3-8†	??	p = 0.69
Intermediate: Neck and Shoulder Pain and Disability Index								
Ylinen et al., 2007 [54]	Pillar + Upper Limb + Another Intervention	18.67	17.49	57	13.00	18.24	59	0.32 [-0.05, 0.68]
Long Term: Self Reporting Disability								
Jordan et al., 1998 [14]	Manipulation + Another Intervention	5.00*	4-7†	??	5.00*	3-6†	??	p = 0.66
Jordan et al., 1998 [14]	Passive Physiotherapy + Another Intervention	5.00*	4-7†	??	6.00*	4-7†	??	p = 0.66
Long Term: Neck and Shoulder Pain and Disability Index								
Ylinen et al., 2007 [54]	Pillar + Upper Limb + Another Intervention	14.00	15.97	57	14.00	18.24	59	0.00 [-0.36, 0.36]
Long Term: NDI								
Ylinen et al., 2007 [54]	Pillar + Upper Limb + Another Intervention	13.33	10.65	57	12.67	13.68	59	0.05 [-0.31, 0.42]
**Upper Limb + Segmental + Motor Control + Another Intervention**								
Pain								
Short Term: VAS								
Yildiz et al., 2017 [62]	Segmental + Motor Control + Another Intervention	1.20	1.40	13	1.10	1.30	12	0.07 [-0.71, 0.86]
Disability								
Short Term: NDI								
Yildiz et al., 2017 [62]	Segmental + Motor Control + Another Intervention	5.90	3.10	13	5.20	3.40	12	0.21 [-0.58, 1.00]
**XX + Upper Limb**								
Pain								
Short Term: NRS								

	Comparator	Exercise Training Programme Mean	SD	Total	Comparator Mean	SD	Total	Effect Estimate (Std. Mean Difference IV, Random) [95%CIs]
Viljanen et al., 2003 [66]	No Treatment	2.90	2.60	135	2.70	2.50	130	0.08 [-0.16, 0.32]
Viljanen et al., 2003 [66]	Relaxation Techniques	2.90	2.60	135	2.90	2.40	128	0.00 [-0.24, 0.24]
Intermediate Term: NRS								
Viljanen et al., 2003 [66]	No Treatment	2.90	2.80	135	2.90	2.80	130	0.00 [-0.24, 0.24]
Viljanen et al., 2003 [66]	Relaxation Techniques	2.90	2.80	135	3.00	2.70	128	-0.04 [-0.28, 0.21]
Long Term: NRS								
Viljanen et al., 2003 [66]	No Treatment	3.10	2.50	135	3.20	2.50	130	-0.04 [-0.28, 0.20]
Viljanen et al., 2003 [66]	Relaxation Techniques	3.10	2.50	135	3.30	2.60	128	-0.08 [-0.32, 0.16]
Disability								
Short Term: Unique Scale to Authors								
Viljanen et al., 2003 [66]	No Treatment	15.00	14.6	135	14.00	13.80	130	0.07 [-0.17, 0.31]
Viljanen et al., 2003 [66]	Relaxation Techniques	15.00	14.6	135	14.00	12.50	128	0.07 [-0.17, 0.32]
Intermediate Term: Unique Scale to Authors								
Viljanen et al., 2003 [66]	No Treatment	15.00	15.40	135	14.00	13.80	130	0.07 [-0.17, 0.31]
Viljanen et al., 2003 [66]	Relaxation Techniques	15.00	15.40	135	15.00	14.50	128	0.00 [-0.24, 0.24]
Long Term: Unique Scale to Authors								
Viljanen et al., 2003 [66]	No Treatment	19.00	15.50	135	17.00	13.70	130	0.14 [-0.10, 0.38]
Viljanen et al., 2003 [66]	Relaxation Techniques	19.00	15.50	135	19.00	14.70	128	0.00 [-0.24, 0.24]

*Median, †90%CI, ??Data Missing

References:

13. Chung S, Jeong YG. Effects of the craniocervical flexion and isometric neck exercise compared in patients with chronic neck pain: A randomized controlled trial. *Physiother Theory Pract* 2018;34(12):916-25. doi: 10.1080/09593985.2018.1430876 [published Online First: 2018/01/25]
14. Jordan A, Bendix T, Nielsen H, et al. Intensive training, physiotherapy, or manipulation for patients with chronic neck pain. A prospective, single-blinded, randomized clinical trial. *Spine (Phila Pa 1976)* 1998;23(3):311-8; discussion 19. doi: 10.1097/00007632-199802010-00005 [published Online First: 1998/03/21]
49. Waling K, Jarvholm B, Sundelin G. Effects of training on female trapezius Myalgia: An intervention study with a 3-year follow-up period. *Spine (Phila Pa 1976)* 2002;27(8):789-96. doi: 10.1097/00007632-200204150-00002 [published Online First: 2002/04/06]
51. Bobos P, Billis E, Papanikolaou DT, et al. Does Deep Cervical Flexor Muscle Training Affect Pain Pressure Thresholds of Myofascial Trigger Points in Patients with Chronic Neck Pain? A Prospective Randomized Controlled Trial. *Rehabil Res Pract* 2016;2016:6480826. doi: 10.1155/2016/6480826 [published Online First: 2016/12/19]
54. Ylinen J, Hakkinen A, Nykanen M, et al. Neck muscle training in the treatment of chronic neck pain: a three-year follow-up study. *Eura Medicophys* 2007;43(2):161-9. [published Online First: 2007/05/26]
57. Chiu TT, Hui-Chan CW, Chein G. A randomized clinical trial of TENS and exercise for patients with chronic neck pain. *Clin Rehabil* 2005;19(8):850-60. doi: 10.1191/0269215505cr920oa [published Online First: 2005/12/06]
59. Javanshir K, Amiri M, Mohseni Bandpei MA, et al. The effect of different exercise programs on cervical flexor muscles dimensions in patients with chronic neck pain. *J Back Musculoskelet Rehabil* 2015;28(4):833-40. doi: 10.3233/BMR-150593 [published Online First: 2015/03/31]
60. O'Leary S, Falla D, Hodges PW, et al. Specific therapeutic exercise of the neck induces immediate local hypoalgesia. *J Pain* 2007;8(11):832-9. doi: 10.1016/j.jpain.2007.05.014 [published Online First: 2007/07/24]
61. Rudolfsson T, Djupsjobacka M, Hager C, et al. Effects of neck coordination exercise on sensorimotor function in chronic neck pain: a randomized controlled trial. *J Rehabil Med* 2014;46(9):908-14. doi: 10.2340/16501977-1869 [published Online First: 2014/09/04]
62. Yildiz TI, Turgut E, Duzgun I. Neck and Scapula-Focused Exercise Training on Patients With Nonspecific Neck Pain: A Randomized Controlled Trial. *J Sport Rehabil* 2018;27(5):403-12. doi: 10.1123/jsr.2017-0024 [published Online First: 2017/06/13]
63. Borisut S, Vongsirinavarat M, Vachalathiti R, et al. Effects of strength and endurance training of superficial and deep neck muscles on muscle activities and pain levels of females with chronic neck pain. *J Phys Ther Sci* 2013;25(9):1157-62. doi: 10.1589/jpts.25.1157 [published Online First: 2013/11/22]
64. Falla D, Lindstrom R, Rechter L, et al. Effectiveness of an 8-week exercise programme on pain and specificity of neck muscle activity in patients with chronic neck pain: a randomized controlled study. *European journal of pain (London, England)* 2013;17(10):1517-28. doi: 10.1002/j.1532-2149.2013.00321.x [published Online First: 2013/05/08]
65. Li X, Lin C, Liu C, et al. Comparison of the effectiveness of resistance training in women with chronic computer-related neck pain: a randomized controlled study. *Int Arch Occup Environ Health* 2017;90(7):673-83. doi: 10.1007/s00420-017-1230-2 [published Online First: 2017/05/22]
66. Viljanen M, Malmivaara A, Uitti J, et al. Effectiveness of dynamic muscle training, relaxation training, or ordinary activity for chronic neck pain: randomised controlled trial. *BMJ* 2003;327(7413):475. doi: 10.1136/bmj.327.7413.475 [published Online First: 2003/08/30]
67. Suvarnnato T, Puntumetakul R, Uthaikhup S, et al. Effect of specific deep cervical muscle exercises on functional disability, pain intensity, craniovertebral angle, and neck-muscle strength in chronic mechanical neck pain: a randomized controlled trial. *J Pain Res* 2019;12:915-25. doi: 10.2147/JPR.S190125 [published Online First: 2019/03/19]
68. Shiravi S, Letafatkar A, Bertozzi L, et al. Efficacy of Abdominal Control Feedback and Scapula Stabilization Exercises in Participants With Forward Head, Round Shoulder Postures and Neck Movement Impairment. *Sports Health* 2019;11(3):272-79. doi: 10.1177/1941738119835223 [published Online First: 2019/04/24]
69. Gupta S, Sharma S, Kataria C. Craniocervical Flexors Endurance Training: Treatment Approach for Cervical Spondylosis. *Indian Journal of Physiotherapy & Occupational Therapy* 2010;4(2):76-81.
70. Gupta BD, Aggarwal S, Gupta B, et al. Effect of Deep Cervical Flexor Training vs. Conventional Isometric Training on Forward Head Posture, Pain, Neck Disability Index In Dentists Suffering from Chronic Neck Pain. *J Clin Diagn Res* 2013;7(10):2261-4. doi: 10.7860/JCDR/2013/6072.3487 [published Online First: 2013/12/04]
71. Hingarajia DD, Sushant; Tejwani, Nishant. Effect of Deep Cranio-cervical Flexors Training Over Isometric Neck Exercises in Chronic Neck Pain and Disability. *Indian Journal of Physiotherapy & Occupational Therapy* 2012;6(4):48-53.
72. Gallego Izquierdo T, Pecos-Martin D, Lluch Girbes E, et al. Comparison of cranio-cervical flexion training versus cervical proprioception training in patients with chronic neck pain: A randomized controlled clinical trial. *J Rehabil Med* 2016;48(1):48-55. doi: 10.2340/16501977-2034 [published Online First: 2015/12/15]
73. Kaur A, Mali K, Mitra M. To Compare the Immediate Effects of Active Cranio Cervical Flexion Exercise Versus Passive Mobilization of Upper Cervical Spine on Pain, Range of Motion and Cranio Cervical Flexion Test in Patients with Chronic Neck Pain. *Indian Journal of Physiotherapy & Occupational Therapy* 2018;12(3):22-27.

74. Kim JY, Kwag KI. Clinical effects of deep cervical flexor muscle activation in patients with chronic neck pain. *J Phys Ther Sci* 2016;28(1):269-73. doi: 10.1589/jpts.28.269 [published Online First: 2016/03/10]
75. Kwan-Woo L, Won-Ho K. Effect of thoracic manipulation and deep craniocervical flexor training on pain, mobility, strength, and disability of the neck of patients with chronic nonspecific neck pain: a randomized clinical trial. *J Phys Ther Sci* 2016;28(1):175-80. doi: 10.1589/jpts.28.175 [published Online First: 2016/03/10]
76. Randlov A, Ostergaard M, Manniche C, et al. Intensive dynamic training for females with chronic neck/shoulder pain. A randomized controlled trial. *Clin Rehabil* 1998;12(3):200-10. doi: 10.1191/026921598666881319 [published Online First: 1998/08/04]
77. Khan M, Soomro RR, Ali SS. The effectiveness of isometric exercises as compared to general exercises in the management of chronic non-specific neck pain. *Pak J Pharm Sci* 2014;27(5 Suppl):1719-22. [published Online First: 2014/09/30]
78. Ulug N, Yilmaz OT, Kara M, et al. Effects of Pilates and yoga in patients with chronic neck pain: A sonographic study. *J Rehabil Med* 2018;50(1):80-85. doi: 10.2340/16501977-2288 [published Online First: 2017/11/22]
